# Radical condensation between benzylic alcohols and acetamides to form 3-arylpropanamides†

**DOI:** 10.1039/d0sc02948c

**Published:** 2020-06-25

**Authors:** Kobra Azizi, Robert Madsen

**Affiliations:** Department of Chemistry, Technical University of Denmark 2800 Kgs. Lyngby Denmark rm@kemi.dtu.dk

## Abstract

A new radical condensation reaction is developed where benzylic alcohols and acetamides are coupled to generate 3-arylpropanamides with water as the only byproduct. The transformation is performed with potassium *tert*-butoxide as the only additive and gives rise to a variety of 3-arylpropanamides in good yields. The mechanism has been investigated experimentally with labelled substrates, trapping experiments and spectroscopic measurements. The findings indicate a radical pathway where potassium *tert*-butoxide is believed to serve a dual role as both base and radical initiator. The radical anion of the benzylic alcohol is proposed as the key intermediate, which undergoes coupling with the enolate of the amide to form the new C–C bond. Subsequent elimination to the corresponding cinnamamide and olefin reduction then affords the 3-arylpropanamides.

## Abstract 2

Benzylic alcohols and acetamides are coupled into 3-arylpropanamides by a new radical condensation through the radical anion of the alcohol.

## Graphical abstract

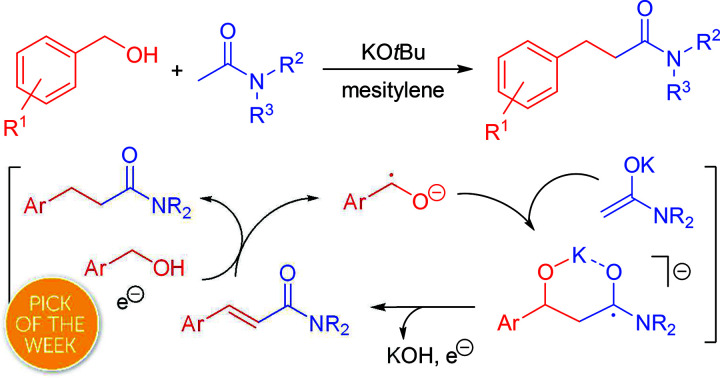

## Introduction

Reactions for the formation of C–C bonds belong to the cornerstone of organic synthesis. Since the turn of the century, an increasing interest has been devoted to C–C bond forming reactions with abundantly available alcohols as the substrates. A particular important development has been acceptorless dehydrogenative transformations^1,2^ where a transition metal catalyst removes dihydrogen from the alcohol to form the corresponding carbonyl compound, which then reacts with a C-nucleophile, such as an enolate, to form the C–C bond. Subsequently, dehydration usually occurs to generate a C=C bond, which may then be saturated by reduction with the liberated dihydrogen. The metal catalyst can be a complex with a platinum group metal^1^ or with a more abundantly available first-row transition metal.^2^ The mechanism for the alcohol dehydrogenation can involve either a metal-hydride or a metal-dihydride species,^1,2^ and in some cases, the ligand also actively participates through metal–ligand bifunctional pathways.^3^

Another and mechanistically different alcohol dehydrogenation method is the Oppenauer oxidation,^4^ which after an aldol reaction and a Meerwein–Ponndorf–Verley reduction^5^ can lead to similar products as the metal-catalyzed acceptorless dehydrogenation. In this case, a transition metal is not required since the transformation can be mediated by a main group metal hydroxide or alkoxide. The mechanism involves hydride transfer through a six-membered transition state and dihydrogen is therefore not formed during the process.^4,5^ An example is the β-alkylation of secondary alcohols and α-alkylation of ketones with primary alcohols, which can be mediated by alkali metal hydroxides and *tert*-butoxides.^6^ Another example is the Guerbet reaction where primary alcohols are dimerized into higher alcohols.^7^ Depending on the catalyst, the hydride transfer in the Guerbet reaction can occur either by acceptorless dehydrogenation or by a Meerwein–Ponndorf–Verley/Oppenauer redox process.^7^ The advantage of these transformations with alcohols is that a stoichiometric amount of waste is not produced since water is often formed as the only byproduct.

Radical mechanisms can also be involved in C–C bond forming reactions with alcohols. α-Hydroxyalkyl radicals can be generated photochemically from alcohols and dimerized into 1,2-diols or perform conjugate addition to olefins with electron-withdrawing groups.^8^ Activation of benzylic alcohols with stoichiometric titanium or phosphorus reagents in the presence of a metal catalyst has been proposed to form benzylic radicals, which can perform cross coupling reactions with aryl halides and conjugate addition to electron-deficient olefins.^9^ Recently, benzylic alcohols were coupled with arylacetylenes in the presence of potassium *tert*-butoxide to form 1,3-diarylpropan-1-ones.^10^ In this transformation, a ketyl radical is believed to be formed from the alcohol based on experimental evidence and the radical then reacts with the alkyne followed by several hydrogen transfer reactions to generate the product.^10^ Although, potassium *tert*-butoxide strictly serves as a base in this coupling, the alkoxide has also been shown to promote radical coupling reactions such as the formation of biaryls from aryl halides and arenes.^11^

Potassium *tert*-butoxide is a very commonly employed base for dehydrogenative transformations with alcohols where the mechanism either involves metal-catalyzed acceptorless dehydrogenation or Meerwein–Ponndorf–Verley/Oppenauer processes.^1,2,12^ We recently developed a manganese(iii) porphyrin-catalyzed coupling between alcohols and amines to form imines, tertiary amines and quinolines where potassium *tert*-butoxide was employed in some cases.^13^ Attempts were made to extend the reaction to α-alkylation of acetamides with alcohols to form 3-substituted propanamides. Surprisingly, the best results were obtained with potassium *tert*-butoxide as the only additive, and more surprisingly, mechanistic studies indicated the condensation to take place by a radical pathway.

Herein, we describe the radical coupling between benzylic alcohols and acetamides to form 3-arylpropanamides with water as the only stoichiometric byproduct.^14,15^ We are not aware of a similar radical condensation with the release of water and the reaction illustrates the importance of mechanistic investigations when developing new dehydrogenative transformations with alcohols.

## Results and discussion

Benzyl alcohol and *N*,*N*-dimethyl acetamide were selected as the substrates for the initial investigations. In the first experiment, a 1 : 2 molar ratio of the alcohol and the amide were treated with 5% of manganese(iii) 5,10,15,20-tetraphenylporphyrin chloride and 1 equiv. of potassium *tert*-butoxide in refluxing mesitylene (Table 1, entry 1). Under these conditions, full conversion of the alcohol occurred as determined by GC and *N*,*N*-dimethyl cinnamamide (**1**) was formed in 28% yield as measured by NMR with 1,3,5-trimethoxybenzene as the internal standard. The main product, however, was benzaldehyde resulting from dehydrogenation with the manganese catalyst. Notably, when the same experiment was performed in the absence of the manganese complex, the major product was *N*,*N*-dimethyl 3-phenylpropanamide (**2**) and only minor amounts of the corresponding cinnamamide was formed (entry 2).

**Table 1:** Optimization of base-mediated coupling between benzyl alcohol and *N*,*N*-dimethyl acetamide^a^

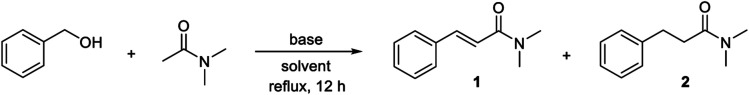					
Entry	Base	Solvent	BnOH conversion^b^ (%)	**1** : **2** ratio	Yield of **2**^c^ (%)
1^d^	KO*t*Bu (1 equiv.)	Mesitylene	100	100 : 0	0
2	KO*t*Bu (1 equiv.)	Mesitylene	85	10 : 90	68
3	KO*t*Bu (1 equiv.)	Toluene	80	50 : 50	30
4	KO*t*Bu (0.2 equiv.)	Mesitylene	65	40 : 60	20
5	KO*t*Bu (0.2 equiv.)	Toluene	60	90 : 10	<5
**6** e	**KO*t*Bu (2 equiv.)**	**Mesitylene**	**100**	**0** **:** **100**	**85**
7	KO*t*Bu (2 equiv.)	Toluene	85	10 : 90	70
8	KO*t*Bu (3 equiv.)	Toluene	90	0 : 100	62
9	KO*t*Bu (2 equiv.)	Dioxane	60	20 : 80	35
10	KO*t*Bu (2 equiv.)	Acetonitrile	50	30 : 70	20
11	KO*t*Bu (2 equiv.)	DMF	35	80 : 20	<5
12	KO*t*Bu (2 equiv.)	None	50	70 : 30	10
13	None	Mesitylene	0	—	0
14	NaO*t*Bu (2 equiv.)	Mesitylene	80	0 : 100	75
15	KOH (2 equiv.)	Mesitylene	0	—	0
16	Cs_2_CO_3_ (2 equiv.)	Mesitylene	30	100 : 0	0
17	Na_2_CO_3_ (2 equiv.)	Mesitylene	0	—	0
18^f^	K_2_CO_3_ (2 equiv.)	Mesitylene	20	—	0
19^f^	NaOH (2 equiv.)	Mesitylene	90	70 : 30	10
20	NaH (2 equiv.)	Mesitylene	15	18 : 82	<5
21	KH (2 equiv.)	Mesitylene	20	10 : 90	10

a Conditions: BnOH (1 mmol), CH_3_CONMe_2_ (2 mmol), base, 1,3,5-trimethoxybenzene (0.5 mmol, internal standard), solvent (3 mL), reflux, 12 h.

b Determined by GC using the internal standard.

c NMR yield with 1,3,5-trimethoxybenzene as the internal standard.

d With 5% of manganese(iii) 5,10,15,20-tetraphenylporphyrin chloride added and reaction time 48 h.

e Reaction time 6 h.

f With 2 mL of *N*,*N*-dimethyl acetamide.

Thus, it was decided to optimize this condensation reaction by investigating the influence of the solvent and the base. When the transformation was performed in refluxing toluene, a 1 : 1 mixture of the two amides were formed leading to a low yield of propanamide **2** (entry 3). Lowering the amount of potassium *tert*-butoxide led to even lower yields due to incomplete conversion of benzyl alcohol (entries 4 and 5). However, increasing the amount of potassium *tert*-butoxide to 2 equiv. gave full conversion of the alcohol already after 6 h in mesitylene solution and afforded **2** as the sole amide product in 85% yield (entry 6). The same experiment in toluene furnished a 9 : 1 mixture of the two amides resulting in a lower yield of **2** (entry 7). The outcome did not improve by using 3 equiv. of potassium *tert*-butoxide in toluene solution although **2** was again formed as the sole amide product (entry 8). Changing the solvent to dioxane, acetonitrile or DMF or performing the condensation under neat conditions all led to low yields of **2** due to moderate conversion of the alcohol and formation of amide mixtures (entries 9–12). No conversion of the alcohol occurred in the absence of potassium *tert*-butoxide (entry 13). Replacing the base with sodium *tert*-butoxide again gave exclusive formation of amide **2** although in a slightly lower yield due to some unreacted alcohol remaining (entry 14). A number of other bases were also included in the study, but very poor results were obtained in all cases (entries 15–21). Consequently, the optimum conditions for the condensation uses 2 equiv. of potassium *tert*-butoxide in refluxing mesitylene solution.

The optimized procedure was then applied to a variety of alcohols and amides to investigate the substrate scope of the transformation. The products were isolated by flash chromatography, which furnished compound **2** in 78% yield (Table 2). Electron-donating methyl, methoxy and benzyloxy groups in the *para* position of the benzyl alcohol were well tolerated and all led to a higher 84–86% yield of *N*,*N*-dimethyl 3-arylpropanamides **3**, **4** and **5**. 3,4-Dimethylbenzyl alcohol afforded propanamide **6** in a similar 83% yield while 2,4,6-trimethylbenzyl alcohol gave amide **7** in 75% yield. *o*-Methyl- and *o*-methoxybenzyl alcohol produced amides **8** and **9** in 85 and 72% yield, respectively, while alcohols with additional aromatic moieties such as *p*-phenylbenzyl alcohol and 1-naphthylmethanol gave rise to the corresponding amides **10** and **11** in 74–75% yield. *p*-Fluoro-, *p*-chloro- and *p*-bromobenzyl alcohol were poor substrates for the condensation under the optimized conditions since dehalogenation product **2** was obtained in 50, 70 and 100% yield, respectively, according to GC analysis. Less dehalogenation occurred when potassium *tert*-butoxide was replaced with sodium *tert*-butoxide where *p*-chlorobenzyl alcohol could be converted into amide **12** in 64% isolated yield. Several other *para*-substituted benzyl alcohols also failed to give a good yield of the condensation product under the optimized conditions. *p*-Methylthiobenzyl alcohol afforded desulfurization product **2** in 58% yield according to GC analysis while almost no conversion occurred with *p*-nitrobenzyl alcohol. *p*-Cyano- and *p*-(trifluoromethyl)benzyl alcohol gave complex mixtures of products, which were not further characterized.

**Table 2:** Condensation between benzylic alcohols and acetamides^a^

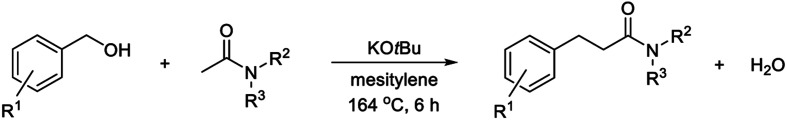
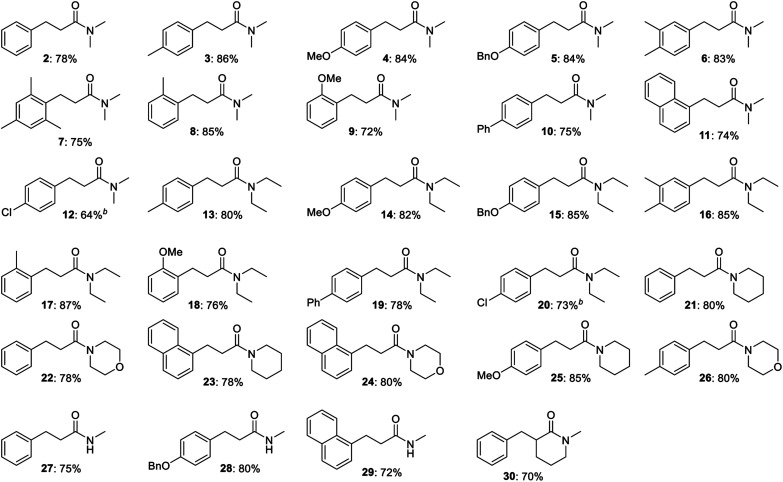

a Conditions: alcohol (1 mmol), amide (2 mmol), KO*t*Bu (2 mmol), mesitylene (3 mL), reflux, 6 h (isolated yields).

b With NaO*t*Bu (2 mmol) instead of KO*t*Bu.

Besides *N*,*N*-dimethyl acetamide, the condensation was also carried out with other *N*-substituted amides (Table 2). *N*,*N*-Diethyl acetamide was reacted with *p*-methyl-, *p*-methoxy-, *p*-benzyloxy- and 3,4-dimethylbenzyl alcohol to afford *N*,*N*-diethyl 3-arylpropanamides **13**, **14**, **15** and **16** in 80–85% yield, which closely resembles the results obtained in the same reactions with *N*,*N*-dimethyl acetamide. The *N*,*N*-diethyl-substituted amide was also reacted with the *ortho*-substituted substrates *o*-methyl- and *o*-methoxybenzyl alcohol to furnish amides **17** and **18** in 87 and 76% yield, respectively. *p*-Phenylbenzyl alcohol gave rise to amide **19** in 78% yield while the *para*-chloro counterpart with sodium *tert*-butoxide as the base afforded amide **20** in 73% yield.

*N*-Acetyl piperidine and morpholine were also subjected to the base-mediated condensation reaction (Table 2). With benzyl alcohol and 1-naphthylmethanol, the corresponding propanamides **21–24** were isolated in 78–80% yield. *p*-Methoxybenzyl alcohol gave amide **25** in the reaction with *N*-acetyl piperidine (85% yield) while *p*-methylbenzyl alcohol afforded amide **26** with *N*-acetyl morpholine (80% yield). In addition, *N*-methyl acetamide could be employed as a substrate where 72–80% yield of amides **27–29** were isolated in the reaction with benzyl alcohol, *p*-benzyloxybenzyl alcohol and 1-naphthylmethanol. α-Substituted acetamides were poor substrates for the condensation since no conversion was observed with *N*,*N*-dimethyl 2-methylpropanamide in the reaction with benzyl alcohol while *N*,*N*-dimethyl propanamide gave very little conversion in the same reaction. The cyclic amide *N*-methyl δ-valerolactam, although, did participate in the condensation and was converted in to the α-benzylated amide **30** in 70% yield. No reaction occurred when the aliphatic alcohols hexan-1-ol and heptan-1-ol were submitted to the optimized conditions in the reaction with *N*,*N*-dimethyl acetamide. Thus, a new potassium *tert*-butoxide-mediated condensation reaction between benzylic alcohols and acetamides has been developed to form a variety of 3-arylpropanamides in good yields.

**Scheme 1:**
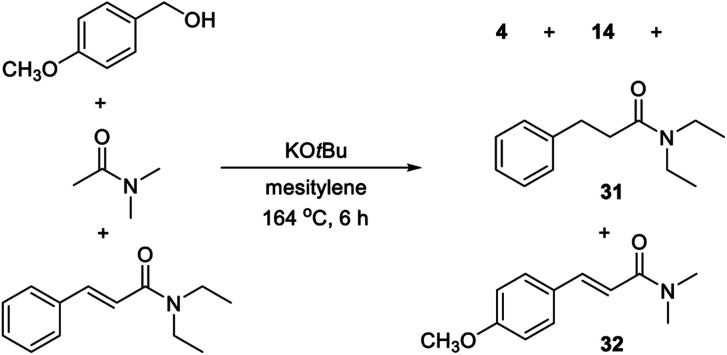
Cinnamamide as a reaction intermediate.

The substrate scope revealed significant dehalogenation and desulfurization of halogen- and sulfur-containing substrates, which are unusual side reactions for transformations proceeding by Meerwein–Ponndorf–Verley/Oppenauer redox processes.^4–6^ Therefore, a number of experiments were performed in order to obtain more detailed information about the reaction mechanism. No benzaldehyde could be detected when the optimized reaction in Table 1, entry 6 was monitored by GC. Instead, small amounts of cinnamamide **1** and *N*,*N*-dimethyl 3-hydroxy-3-phenylpropanamide were observed and both compounds appear to be reaction intermediates. The reduction of a cinnamamide during the transformation was confirmed by a control experiment where 1 equiv. of *p*-methoxybenzyl alcohol was allowed to react with a mixture of 2 equiv. of *N*,*N*-diethyl acetamide and 1 equiv. of *N*,*N*-diethyl cinnamamide (Scheme 1). GC analysis of the reaction mixture revealed a 2 : 1 : 1 : 1 mixture of compounds **4**, **14**, **31** and **32** and thus the conversion of the starting cinnamamide into **31**. Notably, the intermediate cinnamamide is generated in a reversible process since an equal mixture of propanamides **14** and **31** was formed. An experiment was also carried out where benzaldehyde was allowed to react with *N*,*N*-dimethyl acetamide, but no cinnamamide or propanamide was generated in this case and the aldehyde was mainly converted into benzyl benzoate by a Tishchenko reaction. No reaction occurred when the same experiment was performed with benzyl methyl ether revealing the importance of the hydroxy group in the condensation.

To probe the influence of traces of dioxygen, an experiment was carried out with mesitylene that had not been subjected to degassing, but a significantly lower 38% yield of propanamide **2** was observed in this case due to incomplete conversion of the alcohol. Repeating the reaction under an atmosphere of dioxygen or air resulted in the formation of benzaldehyde and cinnamamide **1** due to a base-mediated aerobic alcohol oxidation.^16^ The condensation was also attempted in the presence of 10% of *tert*-butyl hydrogen peroxide, but only 30% conversion of benzyl alcohol into benzaldehyde was observed and none of propanamide **2** was formed. No change in the yield occurred when the reaction in Table 1, entry 6 was performed in the absence of light.

**Scheme 2:**
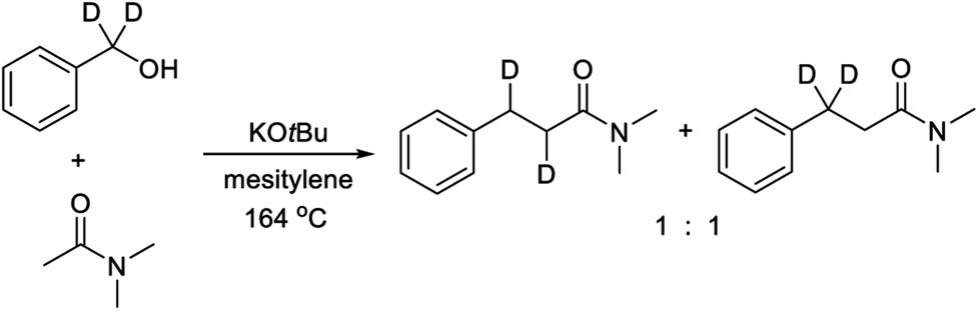
Reaction with deuterium-labelled benzyl alcohol.

The primary kinetic isotope effect (KIE) was determined with both PhCD_2_OH and PhCH_2_OD against PhCH_2_OH by measuring the initial rates. In both cases, a KIE around 1 was observed, which shows that a hydrogen transfer is not involved in the rate-determining step. The products from both labelling experiments were isolated and analyzed by ^1^H and ^2^H NMR. In the reaction with PhCD_2_OH, the product propanamide showed deuterium incorporation in both the α and the β position or exclusively in the β position, and the two labelled compounds were formed in a ratio of approximately 1 : 1 (Scheme 2). In the reaction with PhCH_2_OD, the product showed relatively little deuterium incorporation in either the α or the β position.

Based on these experiments, it is highly unlikely that the coupling takes place by a Meerwein–Ponndorf–Verley/Oppenauer pathway and speculations arose whether a radical mechanism was responsible for the condensation. Therefore, 2 equiv. of several radical scavengers were added to the reaction with benzyl alcohol and *N*,*N*-dimethyl acetamide and the reaction time was extended to 48 h. No coupling product was formed when cyclohexa-1,4-diene and 4-methyl-2,4-diphenylpent-1-ene were included in the mixture and both scavengers underwent conversion into other products according to GCMS analysis. The main product from cyclohexa-1,4-diene was α-cyclohexenylbenzyl alcohol while 4-methyl-2,4-diphenylpent-1-ene afforded 1,2-dimethyl-1,2-diphenylbutane and small amounts of cumene. TEMPO was also employed as a scavenger and led to a lower 50% yield of propanamide **2**. During the transformation TEMPO was mainly reduced to 2,2,6,6-tetramethylpiperidine, but small amounts of 1-benzyl-2,2,6,6-tetramethylpiperidine could also be detected. In addition, 2,6-di-*tert*-butyl-4-methylphenol (BHT) was added as a scavenger and in this case complete conversion of benzyl alcohol was observed, but the product was a 1 : 1 mixture of cinnamamide **1** and propanamide **2**, and BHT was primarily oxidized into 2,6-di-*tert*-butyl-4-methylenecyclohexa-2,5-dien-1-one.

**Fig. 1:**
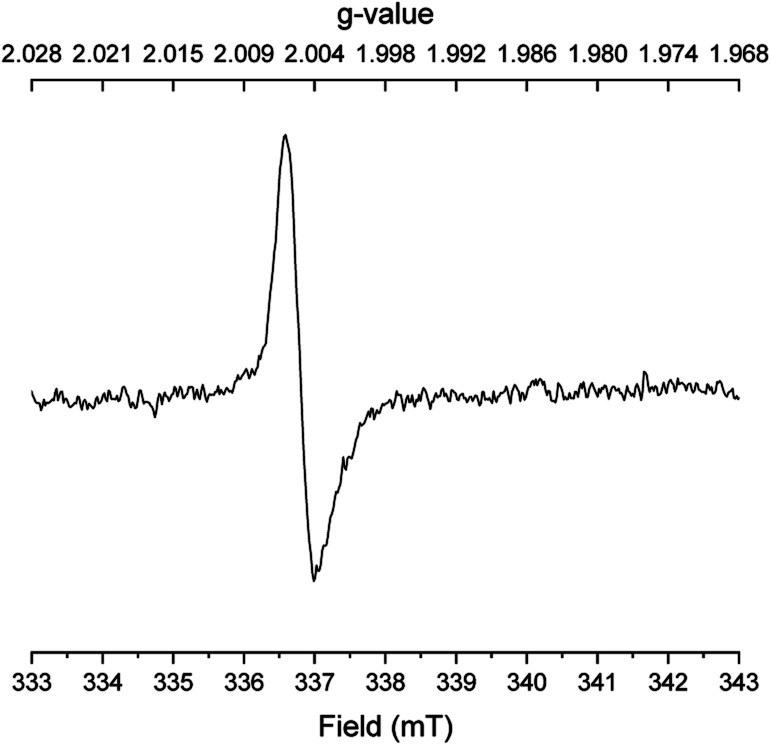
EPR spectrum of the reaction in Table 1, entry 6.

To gain further evidence for radical intermediates, a radical clock experiment was then performed with *o*-allylbenzyl alcohol and *N*,*N*-dimethyl acetamide. No propanamide was formed in this case, but 50% conversion of *o*-allylbenzyl alcohol occurred into a 5 : 3 : 2 mixture of 2-methylindene, 2-methylindane and *o*-propylbenzaldehyde according to GCMS analysis. Finally, several EPR spectra were recorded under different conditions with benzyl alcohol, *N*,*N*-dimethyl acetamide and potassium *tert*-butoxide after 45 min in refluxing mesitylene. With all three reactants in the mixture, a singlet with a *g* value of 2.0045 was observed indicating a carbon-centered radical bound to oxygen (Fig. 1). The same signal, although weaker, was detected when only benzyl alcohol and potassium *tert*-butoxide were subjected to refluxing mesitylene. However, no radicals were observed upon refluxing only the base, the acetamide and the base, or the alcohol and the acetamide in mesitylene solution. These results indicate that the carbon-centered radical is formed in connection with the deprotonation of benzyl alcohol.

**Scheme 3:**
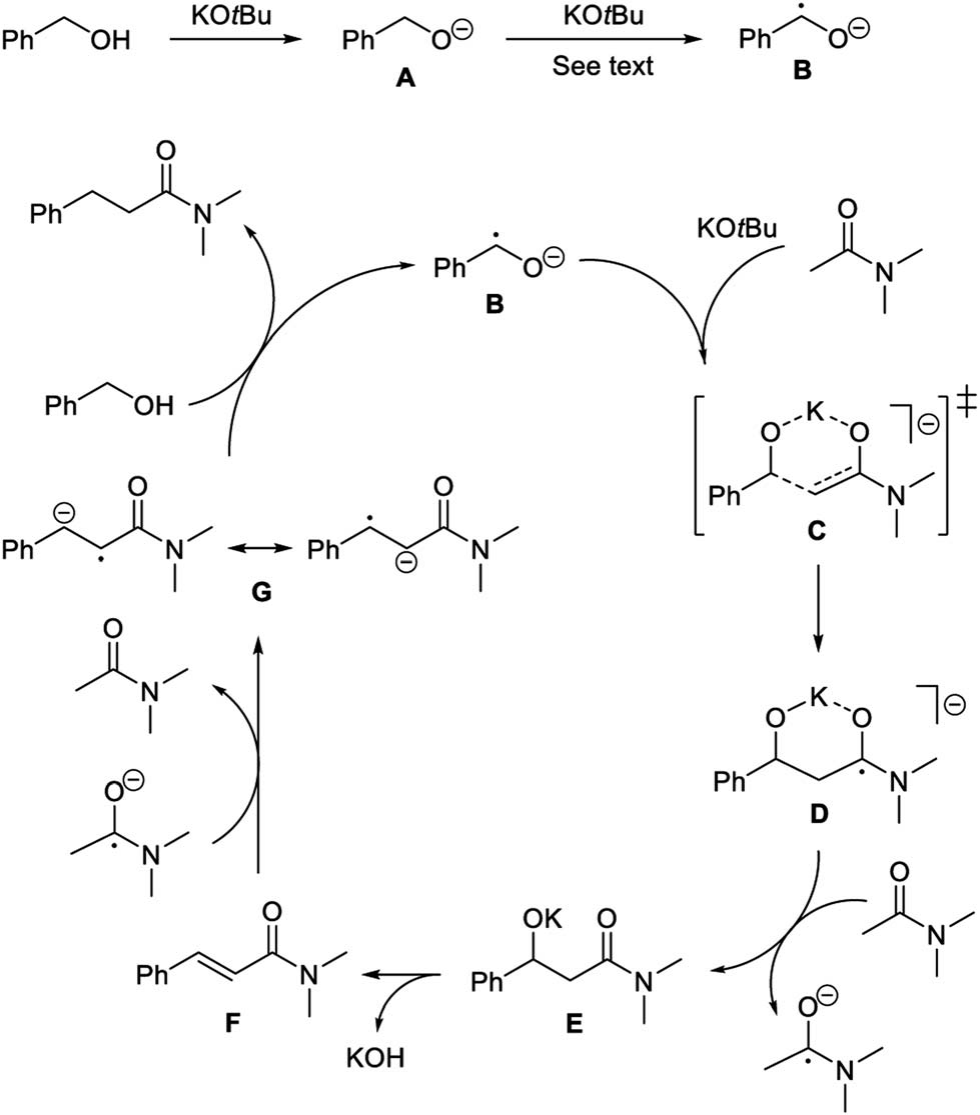
Proposed mechanism for radical condensation.

Accordingly, a mechanism is proposed where benzyl alcohol is deprotonated by the base to form alkoxide **A** (Scheme 3). Initiation then occurs by generating radical anion **B**.^17^ In principle, the α-hydrogen atom transfer can be mediated by traces of dioxygen or the amide. Recently similar hydrogen atom transfer reactions have been proposed from alkoxides to alkynes^10^ and ketones^18^ as part of a radical initiation process. However, in our case a more likely scenario involves potassium *tert*-butoxide for abstracting the α-hydrogen atom in **A**. It has been debated whether potassium *tert*-butoxide is able to serve as a single electron donor to form the alkoxyl radical,^11^ which would rapidly remove an α-hydrogen atom from **A**.^19^ As an example, potassium *tert*-butoxide reduces benzophenone under photoexcitation conditions to the benzophenone ketyl radical anion.^20^ Our transformation, however, is not affected by light and instead potassium *tert*-butoxide may perform an additional deprotonation of **A** to form the dianion PhCHO^2−^. A number of organic compounds have been shown to undergo deprotonation or double deprotonation by potassium *tert*-butoxide to generate anionic intermediates, which can serve as strong single electron donors.^21^ A relevant example is the double deprotonation of 2-pyridinemethanol to yield the dianion capable of initiating haloarene – arene couplings.^22^ Thus, anion **A** is most likely deprotonated by potassium *tert*-butoxide at the elevated temperature to form PhCHO^2−^, which will convert into radical anion **B** by single electron transfer to the acetamide (during the reaction) or to the aromatic moiety of the solvent or the alcohol (in the absence of the amide).

With the formation of the radical anion **B**, a radical chain pathway can be envisioned where **B** first reacts with the enolate of the amide, possibly through a six-membered transition state **C**, to form **D**. This C–C bond forming reaction is most likely the rate-determining step in the condensation. The addition of carbon-centered radicals to enolates has previously been described to form α-alkylated and α-arylated ketones.^23^ Radical anion **D** is an aminoketyl radical,^24^ which can be converted into the corresponding amide **E** by single electron transfer to an acceptor, *e.g. N*,*N*-dimethyl acetamide. Amide **E** then undergoes elimination under the basic conditions to form cinnamamide **F**. Finally, the olefin is reduced through single electron transfer^25^ to generate radical anion **G**,^26^ which reacts with benzyl alcohol to produce the product propanamide and regenerate radical anion **B**. The proposed pathway is in accordance with the experimental observations from both the deuterium-labelling experiments and the radical trapping reactions.

Thus, the study illustrates the importance of additional mechanistic investigations when performing transformations, which seems to proceed by dehydrogenation of an alcohol. This is especially important when potassium *tert*-butoxide is employed as an additive since the salt can serve both as a base and as a reagent for initiating radical reactions. It should be noted that the previous transition metal-catalyzed syntheses of 3-arylpropanamides from benzylic alcohols and acetamides have all been carried out with potassium *tert*-butoxide as a stoichiometric additive.^14^ Although, the transformations all use known alcohol dehydrogenation catalysts,^14^ it cannot be excluded that the present radical coupling is a minor pathway in some of these reactions.

## Conclusions

In summary, we have described a new radical coupling where 3-arylpropanamides are formed from benzylic alcohols and acetamides in the presence of potassium *tert*-butoxide. A variety of alcohols and amides have been subjected to the transformation to generate the product propanamides in good yields. Experimental mechanistic studies have revealed that the condensation takes place by a radical pathway where the radical anion of the benzylic alcohol is proposed as the key intermediate. The findings illustrate the importance of radical trapping experiments when performing coupling reactions with alcohols, which appear to occur by transition metal-catalyzed acceptorless dehydrogenation or Meerwein–Ponndorf–Verley/Oppenauer pathways. This is especially important when potassium *tert*-butoxide is employed since the salt may serve a dual role as both base and initiator of radical reactions. The discoveries are envisioned to spur further interest in the development of new radical-mediated C–C bond-forming reactions with alcohols.

## Conflicts of interest

There are no conflicts to declare.

## Supplementary Material

SC-011-D0SC02948C-s001
