# Supplementary material for: Radical condensation between benzylic alcohols and acetamides to form 3-arylpropanamides
Source: Chem Sci. 2020 Jun 25;11(30):7800–6. doi: 10.1039/d0sc02948c (PMC8163310; doi:10.1039/d0sc02948c)

Electronic Supplementary Material (ESI) for Chemical Science.
This journal is © The Royal Society of Chemistry 2020

SUPPLEMENTARY INFORMATION

Radical condensation between benzylic alcohols and acetamides to form 3-arylpropanamides

Kobra Azizi and Robert Madsen*

Department of Chemistry, Technical University of Denmark, 2800 Kgs. Lyngby, Denmark

Table of Contents

	Page
General information	2
General procedure for synthesis of 3-arylpropanamides	2
Deuterium labelling studies	2
Radical clock experiment	10
EPR spectra	12
^{1}H and ^{13}C NMR data of products	13
References	21
Copies of ^{1}H and ^{13}C NMR spectra	22

General information

All commercially available reagents were purchased from Sigma-Aldrich and were not further purified. Mesitylene was stored over activated 4Å molecular sieves and degassed with N_2 before being used. Gas chromatography was performed on a Shimadzu GCMS-QP2010S instrument fitted with an Equity 5, 30 m × 0.25 mm × 0.25 μm column using 1,3,5-trimethoxybenzene as an internal standard. Flash column chromatography separations were performed on silica gel 60 (40 – 63 μm). NMR spectra were recorded on a Bruker Ascend 400 spectrometer. Chemical shifts were measured relative to the signals of residual $CHCl_3$ (δ_H = 7.26 ppm) and $CDCl_3$ (δ_C = 77.16 ppm). EPR spectra were recorded on a continuous wave X-band Bruker EMX EPR spectrometer with the ER 4102ST cavity with a gunn diode microwave source in the field interval 220 – 400 mT. A spectrum was run using the empty EPR tube and the spectra shown were corrected by subtracting this background spectrum.

General procedure for synthesis of 3-arylpropanamides

An oven-dried tube (20 mL) was charged with KO*t*Bu (224 mg, 2 mmol) and placed in a Radley carousel on a hotplate. The tube was evacuated and then filled with N_2 gas (repeated 5 times). Freshly degassed mesitylene (3 mL) was injected into the tube followed by heating to 164 °C under a N_2 atmosphere. Then, the alcohol (1 mmol) and the amide (2 mmol) were added and the reaction was refluxed for 6 h. The mixture was cooled to room temperature and the solvent removed in vacuo. The residue was purified by silica gel column chromatography (2:1 hexane/EtOAc → EtOAc) to obtain the desired product.

Deuterium labelling studies

The reaction with $C_6H_5CD_2OH$ and *N*,*N*-dimethyl acetamide was performed in mesitylene solution using the general procedure. ^1H, ^2H and ^{13}C NMR analysis of the product (and a GCMS) showed deuterium incorporation at the α and the β position of the propanamide.

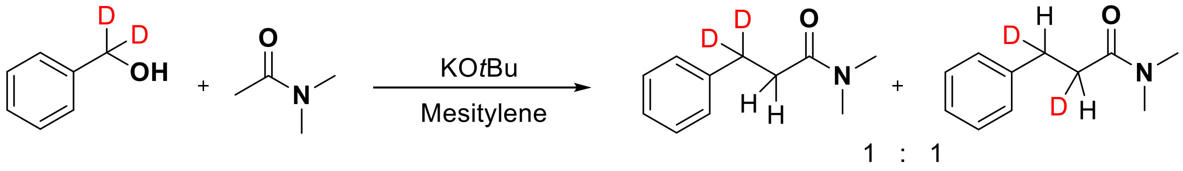

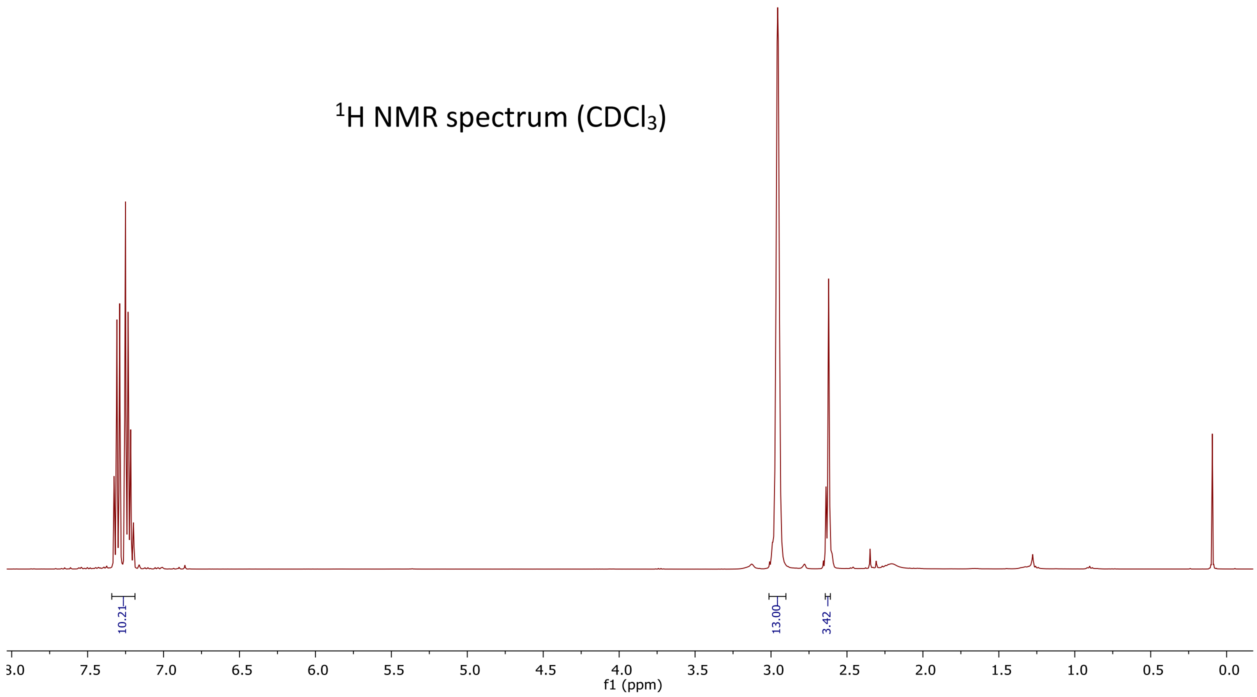

^{1}H NMR spectrum (CDCl$_3$)
10.21
13.00
3.42
f1 (ppm)

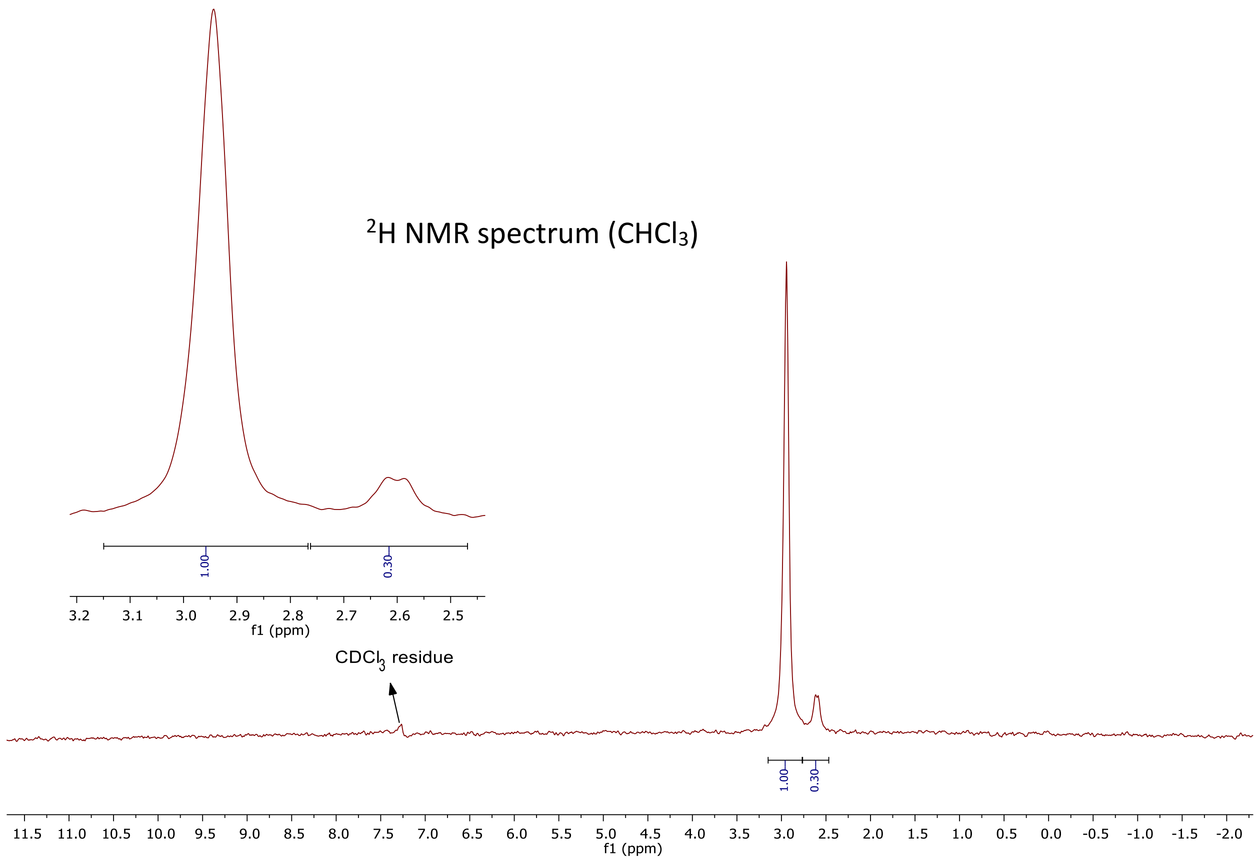

^{2}H NMR spectrum (CHCl$_3$)
1.00
0.30
f1 (ppm)
CDCl$_3$ residue

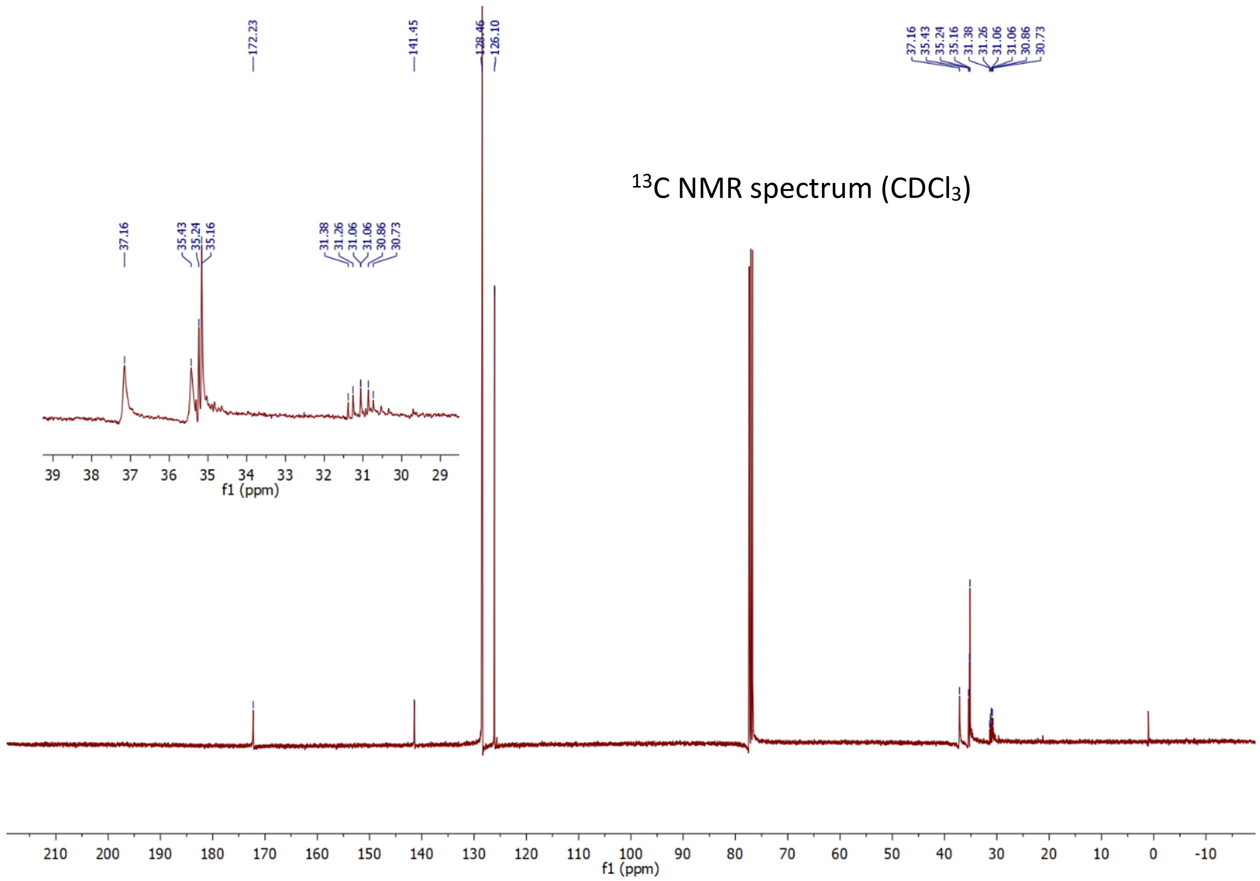

^{13}C NMR spectrum ($CDCl_3$)
172.23
141.45
128.46
126.10
37.16
35.43
35.24
35.16
31.38
31.26
31.06
31.06
30.86
30.73
f1 (ppm)

GCMS (EtOAc)

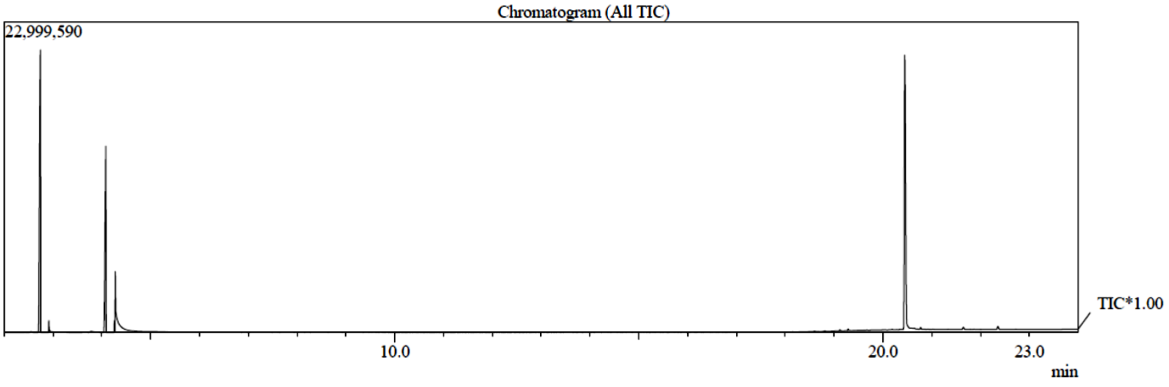
Chromatogram (All TIC)
22,999,590
TIC*1.00
10.0
20.0
23.0
min

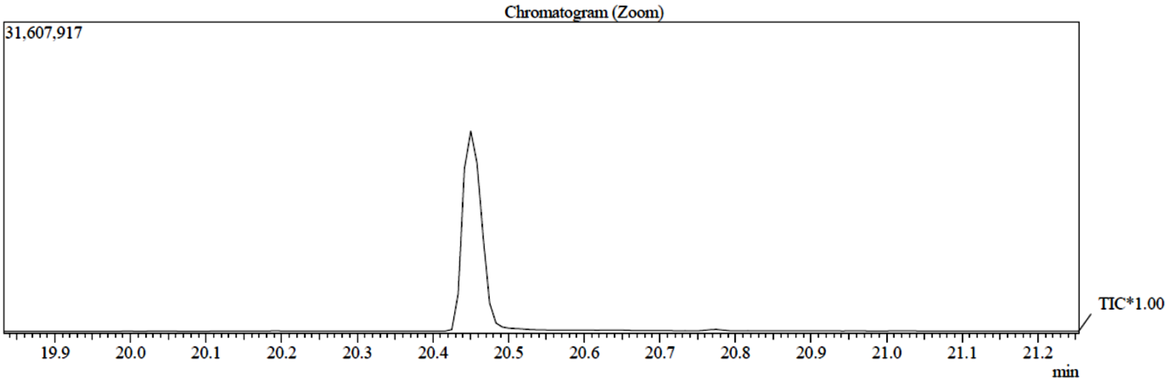
Chromatogram (Zoom)
31,607,917
TIC*1.00
19.9
20.0
20.1
20.2
20.3
20.4
20.5
20.6
20.7
20.8
20.9
21.0
21.1
21.2
min

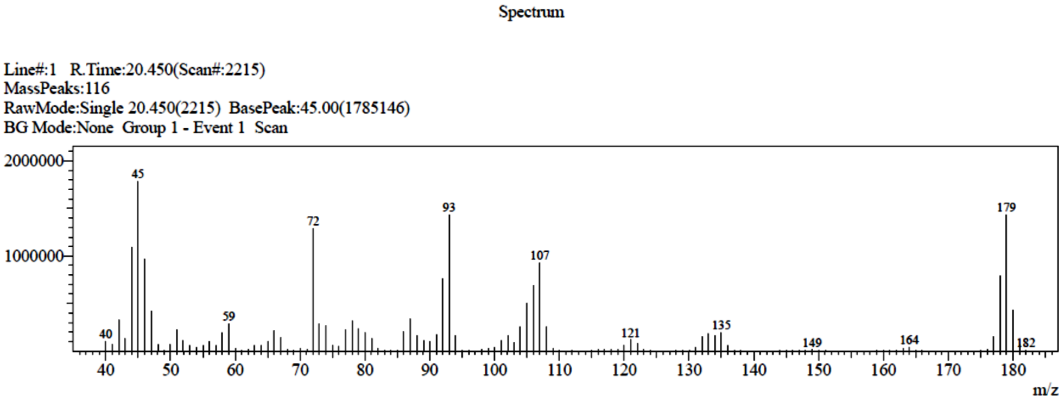
Spectrum
Line#:1 R.Time:20.450(Scan#:2215)
MassPeaks:116
RawMode:Single 20.450(2215) BasePeak:45.00(1785146)
BG Mode:None Group 1 - Event 1 Scan
2000000
1000000
40
45
59
72
93
107
121
135
149
164
179
182
40
50
60
70
80
90
100
110
120
130
140
150
160
170
180
m/z

The reaction with $C_6H_5CH_2OD$ and *N*,*N*-dimethyl acetamide was performed in mesitylene solution using the general procedure. ^1H, ^2H and ^{13}C NMR analysis of the product (and a GCMS) showed relatively little deuterium incorporation at the α and the β position of the propanamide.

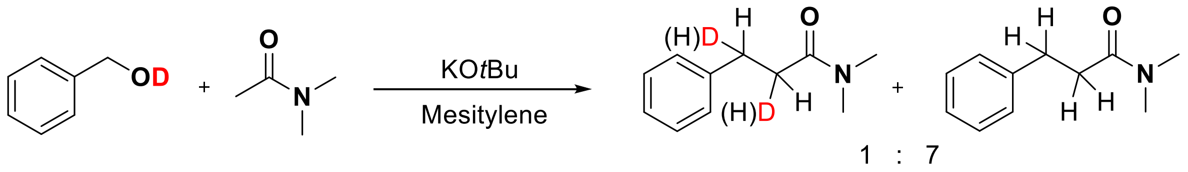

^1H NMR spectrum ($CDCl_3$)

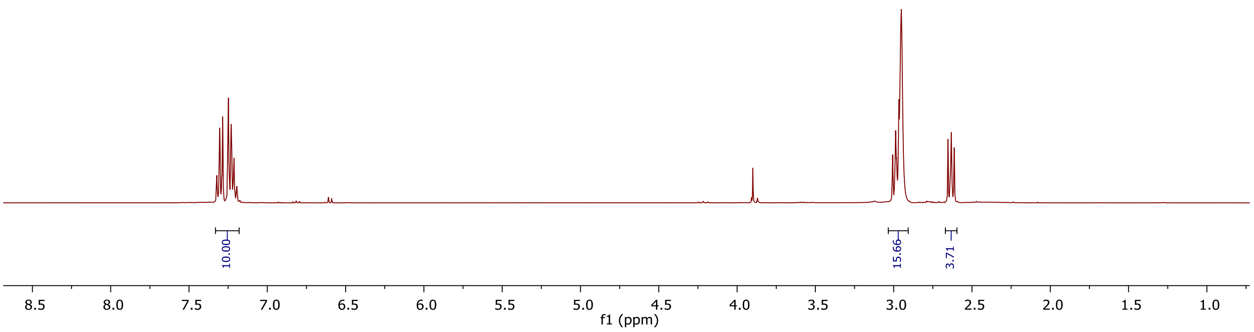

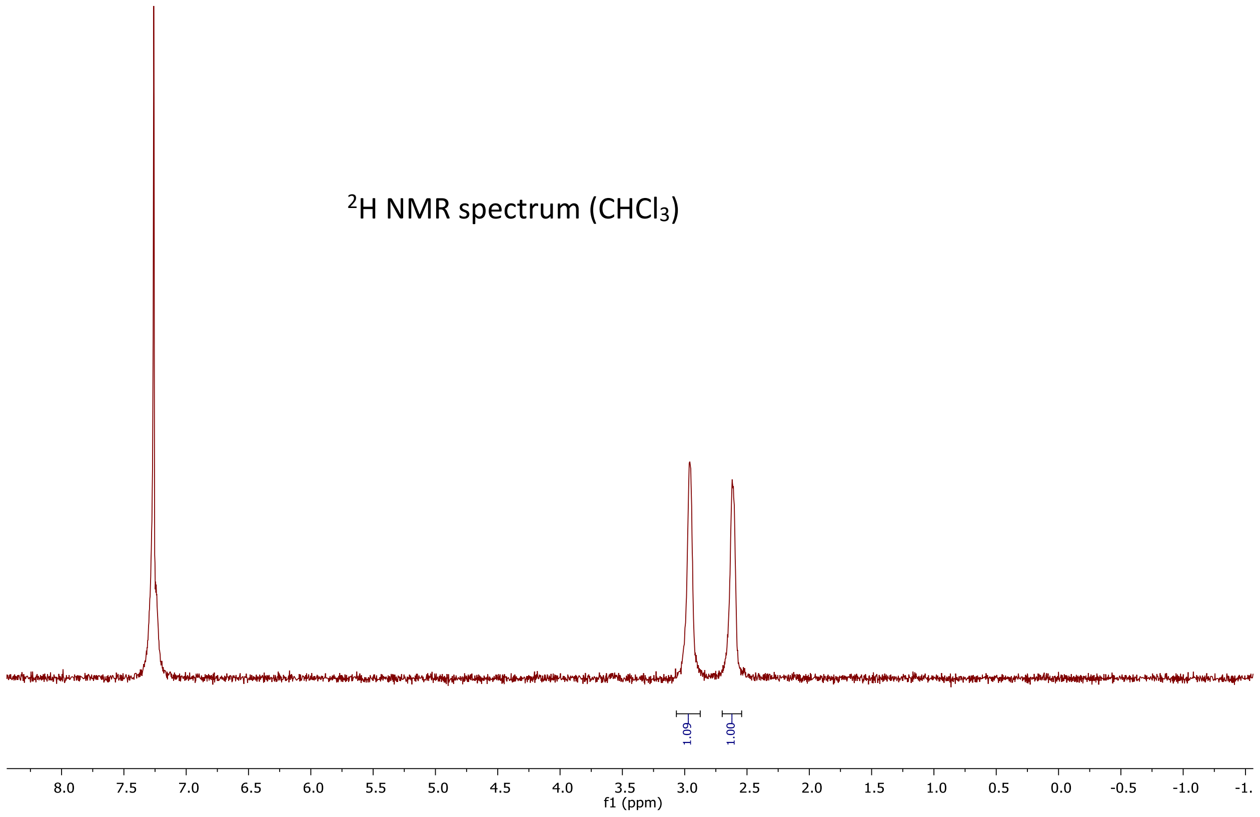

^{2}H NMR spectrum (CHCl$_3$)
1.09
1.00
f1 (ppm)

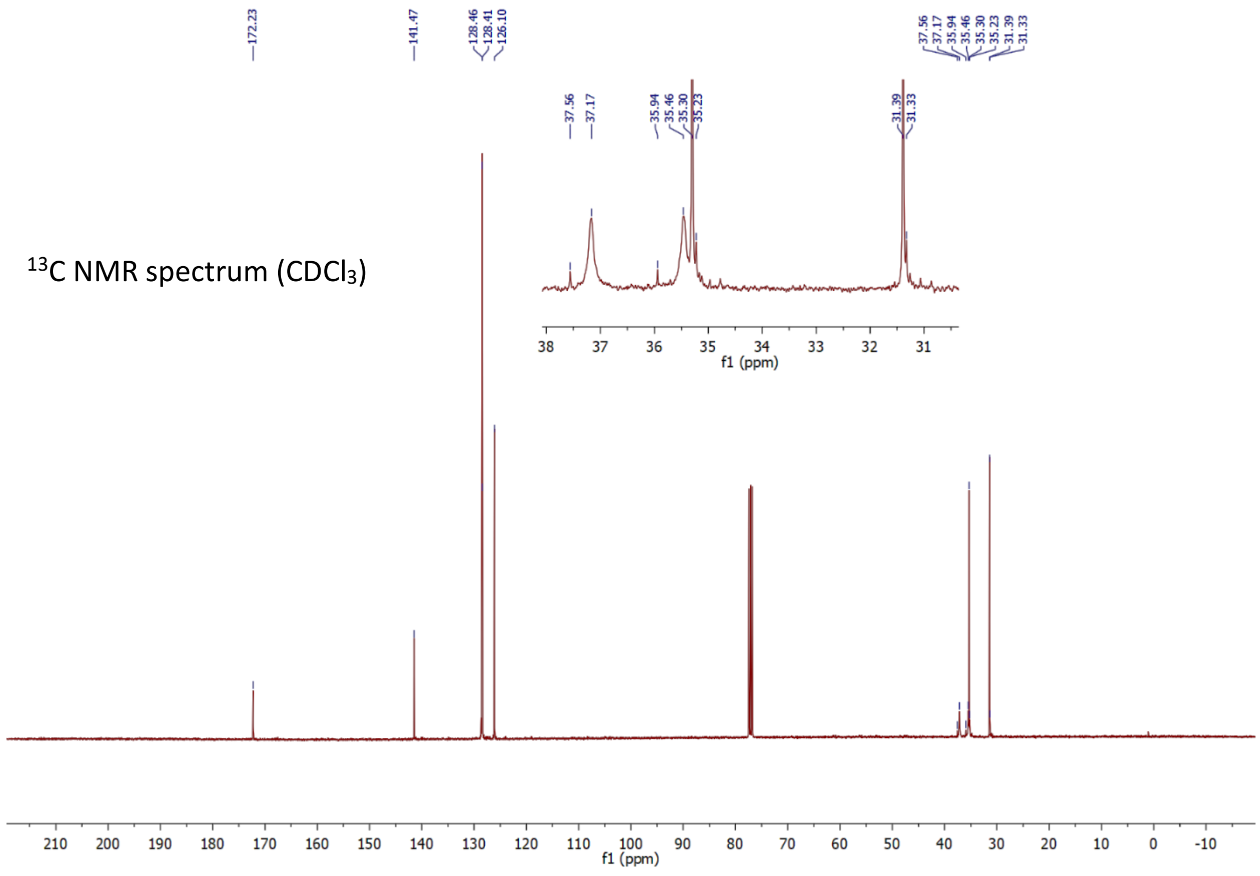

172.23
141.47
128.46
128.41
126.10
37.56
37.17
35.94
35.46
35.30
35.23
31.39
31.33
^{13}C NMR spectrum (CDCl$_3$)
f1 (ppm)

GCMS (EtOAc)

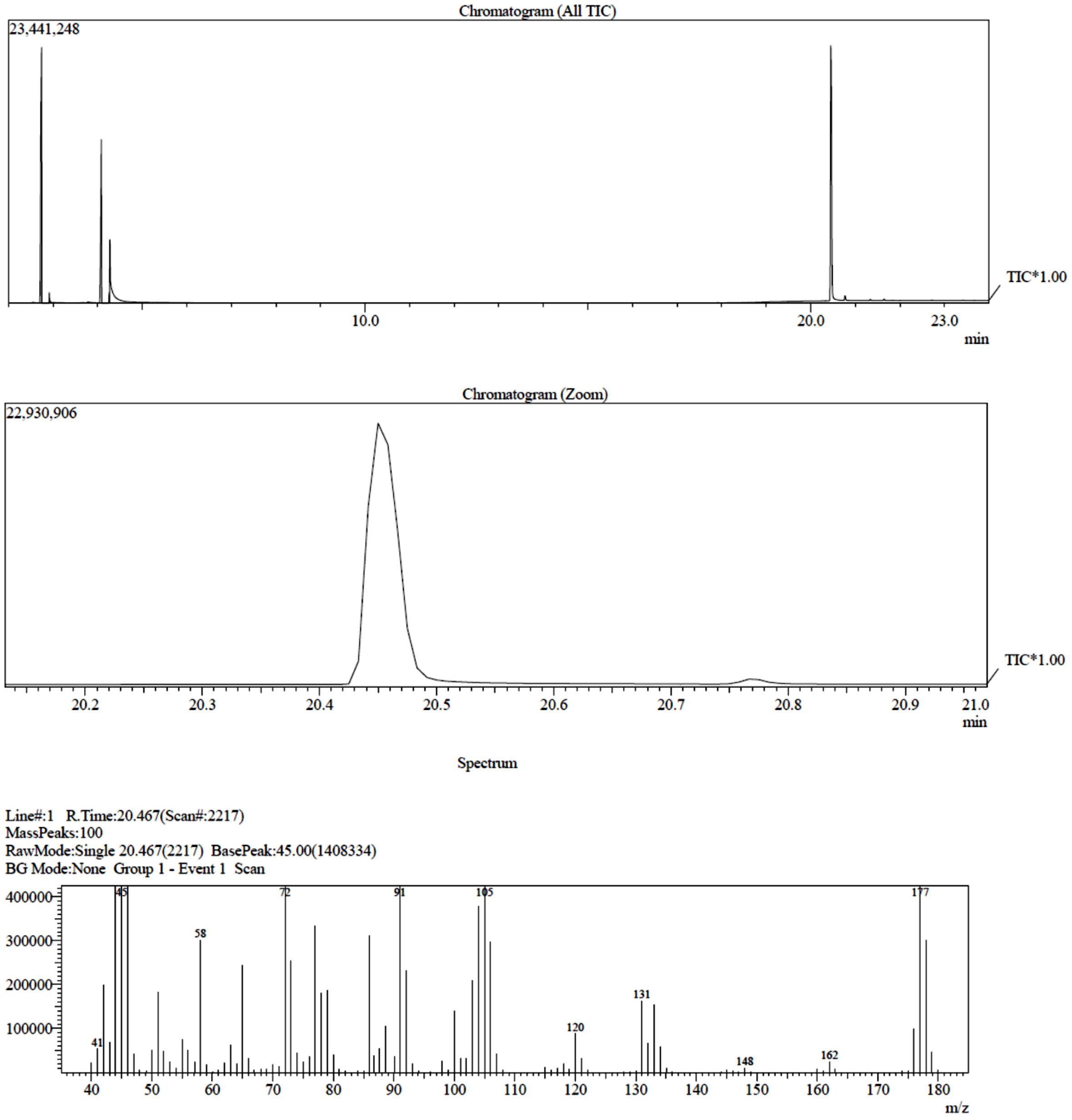

The deuterium isotope effect was determined with both $PhCD_2OH$ and $PhCH_2OD$. An oven-dried tube was charged with KO*t*Bu (224 mg, 2.0 mmol) and 1,3,5-trimethoxybenzene (TMB) (84 mg, 0.5 mmol, internal standard) and placed in a Radley carousel on a hotplate. The tube was evacuated and then filled with N_2 gas. Freshly degassed mesitylene (3 mL) was injected into the tube followed by heating to 164 °C under a N_2 atmosphere. The alcohol (1.0 mmol, $PhCH_2OH$, $PhCD_2OH$ or $PhCH_2OD$) and *N*,*N*-dimethyl acetamide (174 mg, 2.0 mmol) were

added and the course of the reaction was monitored by GC for 2.5 h. Every 15 min a sample was taken out and subjected to GC analysis to follow the disappearance of the benzylic alcohol (and thus the formation of *N*,*N*-dimethyl 3-phenylpropanamide). Initial rate plots were performed to determine the k_H for $PhCH_2OH$ as well as the k_D for $PhCD_2OH$ and $PhCH_2OD$ and thus the KIE = k_H/k_D.

KIE plot of $PhCD_2OH/PhCH_2OH$:

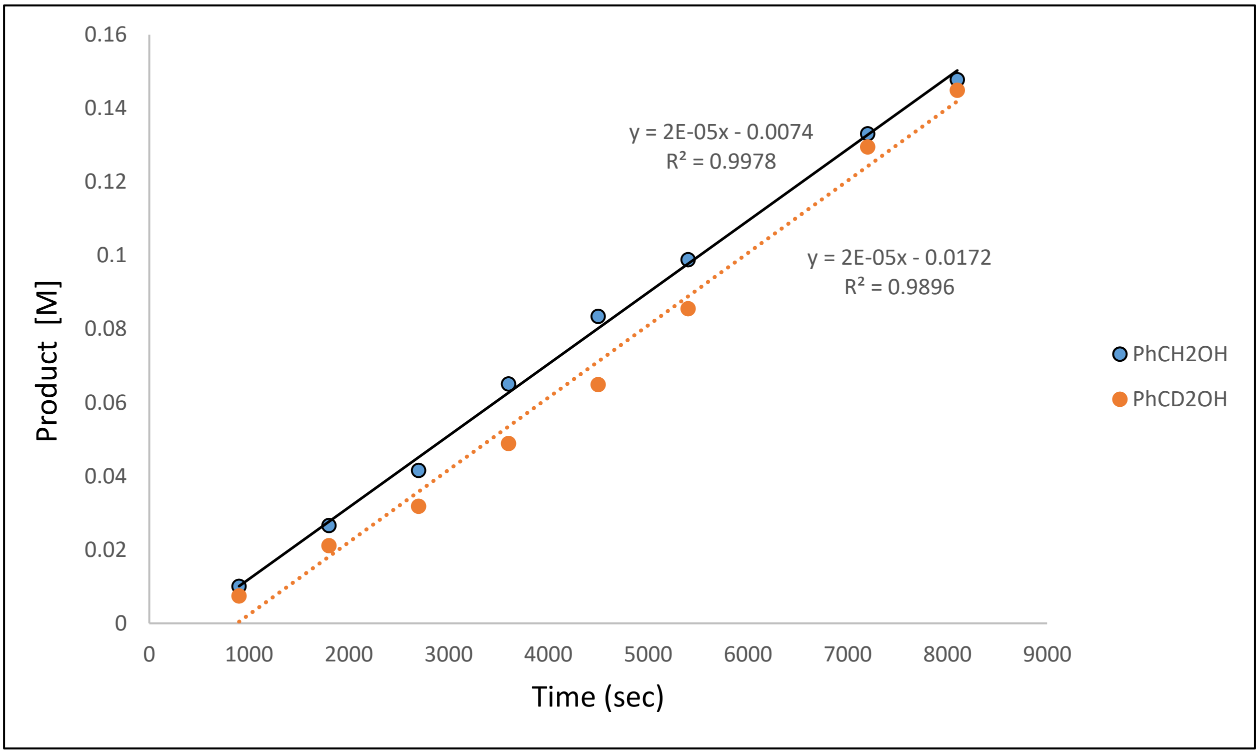

Excel data for the KIE plot of $PhCD_2OH/PhCH_2OH$:

A	B	C	D	E	F	G	H	I	J
t	time(s)	H(TMB)	D(TMB)	P(H)= Alcohol (t0)-Alcohol (tx)	P(D)= Alcohol (t0)-Alcohol (tx)	Yield H %	Yield D %	Conc.(H)prod	Conc.(D)pr
0	0	100	100	0	0	0	0	0	0
1	900	93.68	95.28	6.32	4.72	4.346856293	3.191871041	0.010108968	0.007422956
2	1800	84.92	87.64	15.08	12.36	11.44185507	9.087013814	0.026608965	0.02113259
3	2700	78.28	82.49	21.72	17.51	17.87780065	13.67697109	0.041576281	0.03180691
4	3600	69.71	75.39	30.29	24.61	27.99685687	21.03308403	0.065108969	0.048914149
5	4500	64.23	69.78	35.77	30.22	35.88278183	27.90413609	0.08344833	0.06489334
6	5400	60.25	63.66	39.75	36.34	42.50941889	36.78098666	0.098859114	0.085537178
7	7200	52.2	53.64	46.36	46.36	57.22397347	55.68775942	0.133079008	0.129506417
8	8100	50.34	50.84	49.66	49.16	63.56215169	62.30335284	0.147818957	0.144891518

KIE plot of $PhCH_2OD/PhCH_2OH$:

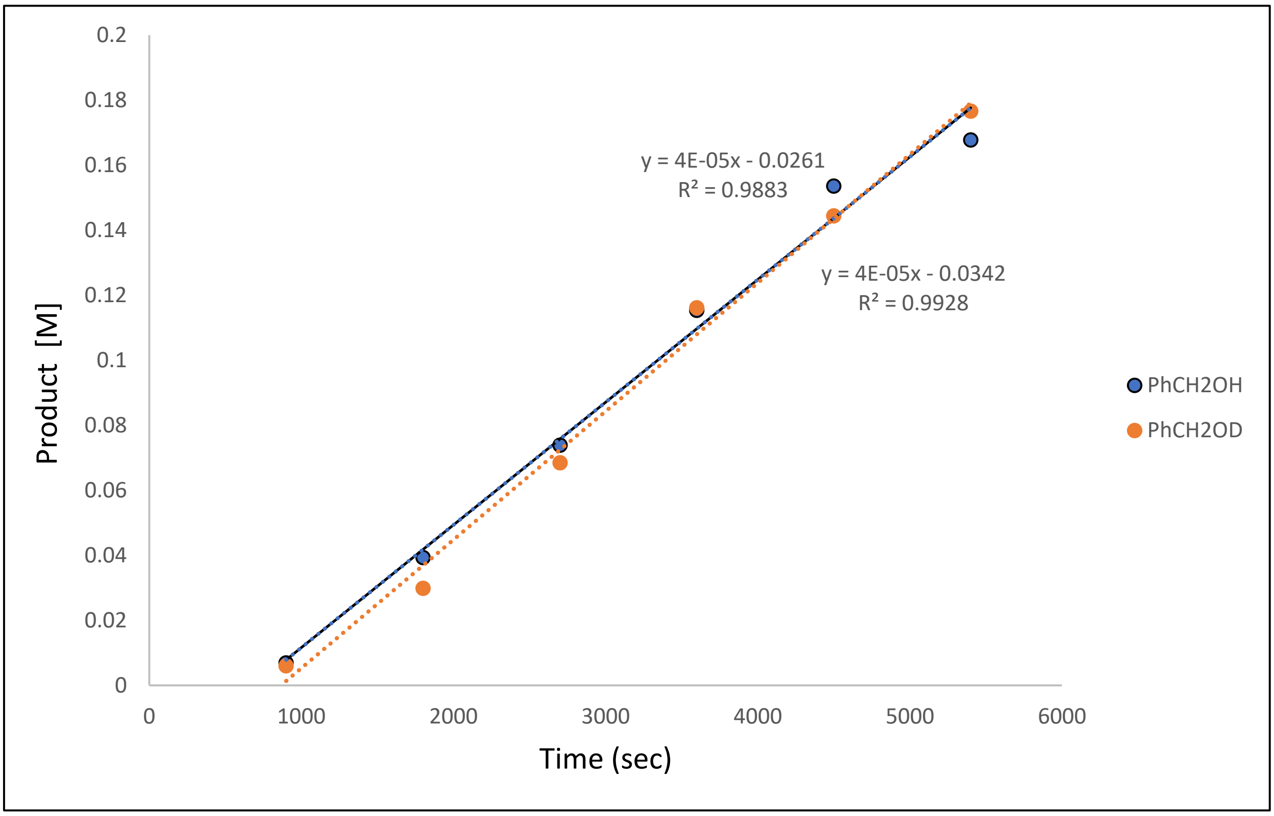

Excel data for the KIE plot of $PhCH_2OD/PhCH_2OH$:

A	B	C	D	E	F	G	H	I	J
t	time (s)	TMB(H)	TMB(D)	P(H)= Alcohol (t_0)-Alcohol (t_x)	P(D)= Alcohol (t_0)-Alcohol (t_x)	Yield H %	Yield D %	Conc.(H)prod	Conc.(D)pr
0	0	100	100	0	0	0	0	0	0
1	900	91.2	92.25	8.8	7.75	2.960558353	2.577633215	0.006885019	0.005994496
2	1800	64.52	70.51	35.48	29.49	16.87232934	12.83245794	0.039237975	0.029842925
3	2700	49.15	51.04	50.85	48.96	31.74338429	29.43178045	0.073821824	0.068446001
4	3600	38.22	38.06	61.78	61.94	49.5955845	49.93306315	0.115338569	0.116123403
5	4500	31.75	33.08	68.3	66.92	66.0028617	62.06921075	0.153495027	0.144347002
5	5400	29.85	28.78	70.15	71.22	72.10562267	75.92712778	0.167687495	0.176574716

Radical clock experiment

The reaction with *o*-allylbenzyl alcohol and *N*,*N*-dimethyl acetamide was performed in mesitylene solution using the general procedure. GCMS analysis of the mixture showed 50% conversion of o-allylbenzyl alcohol into a 5:3:2 mixture of compounds A, B and C.

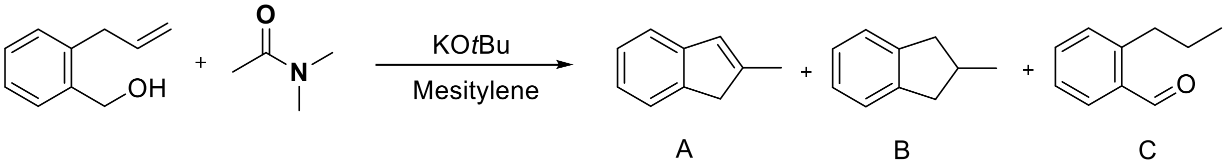

^{1}H NMR spectrum of A ($CDCl_3$)

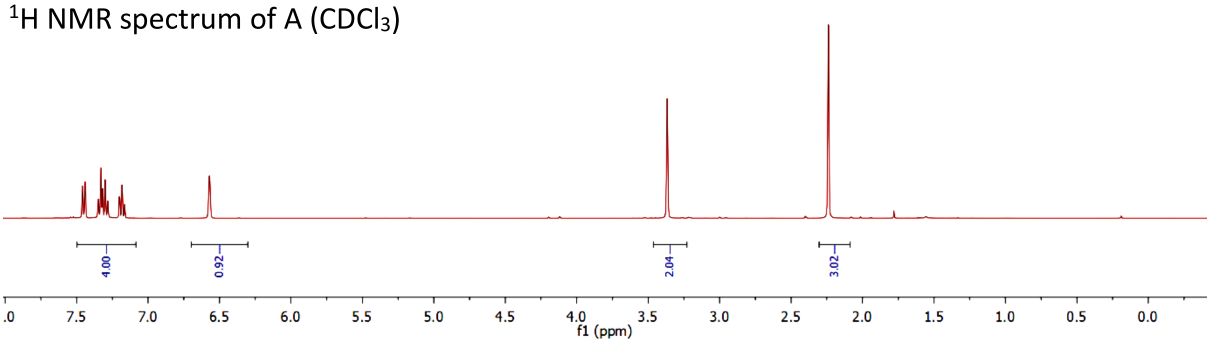

^{1}H NMR spectrum of B ($CDCl_3$)

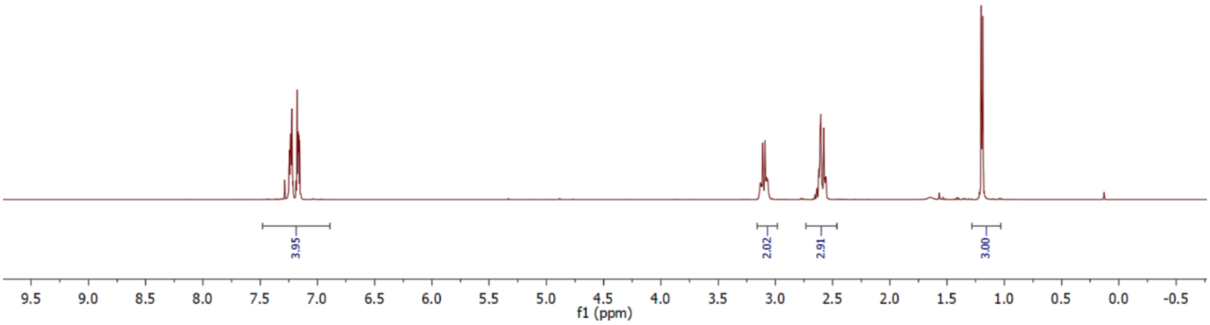

^{1}H NMR spectrum of C ($CDCl_3$)

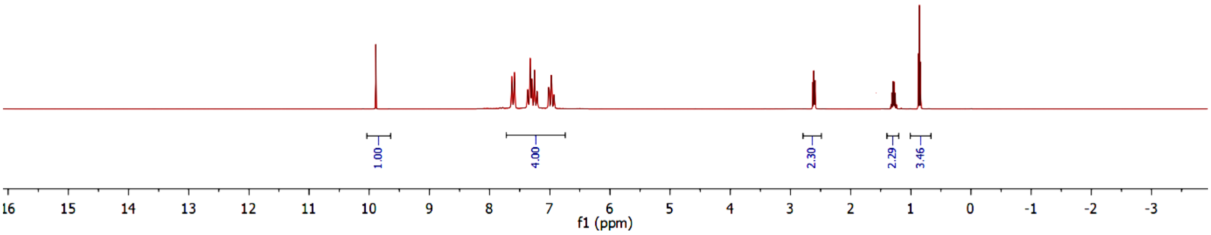

EPR spectra

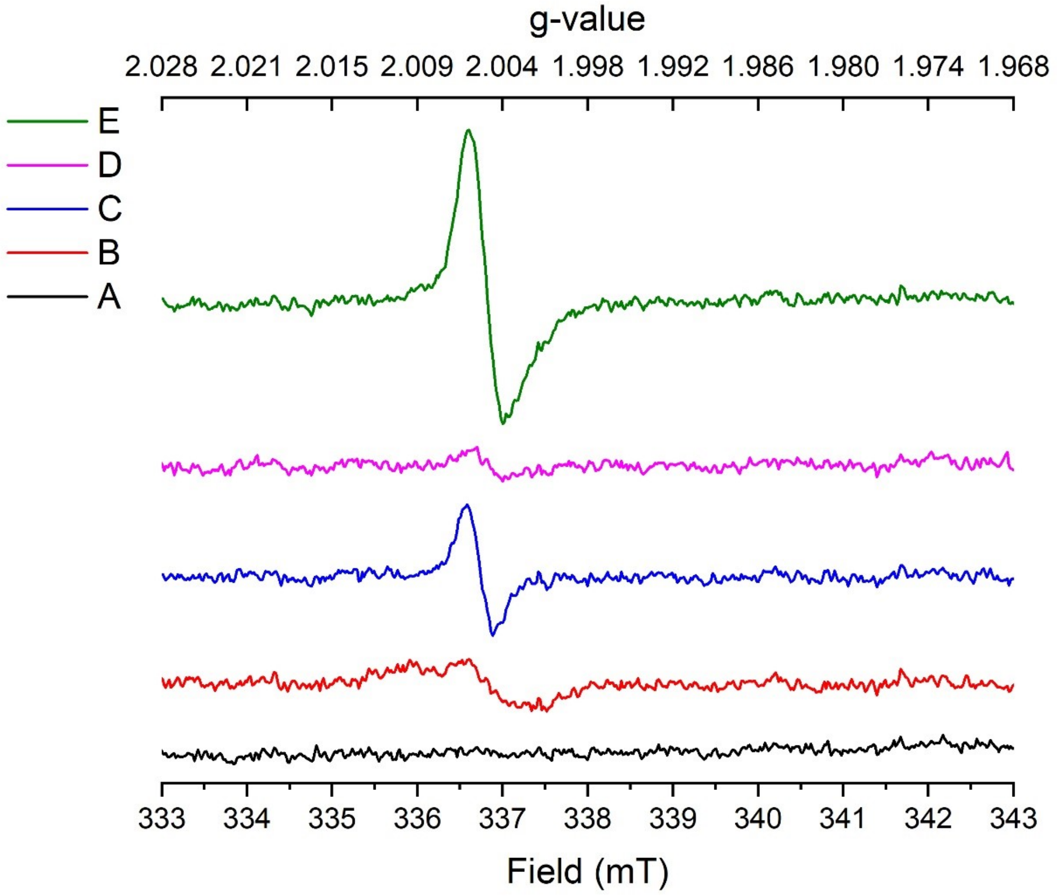

A: Benzyl alcohol and *N*,*N*-dimethyl acetamide in mesitylene at 170 ^oC (no KO*t*Bu)

B: Only KO*t*Bu in mesitylene at 170 ^oC

C: Benzyl alcohol and KO*t*Bu in mesitylene at 170 ^oC

D: *N*,*N*-Dimethyl acetamide and KO*t*Bu in mesitylene at 170 ^oC

E: Benzyl alcohol, *N*,*N*-dimethyl acetamide and KO*t*Bu in mesitylene at 170 ^oC

^{1}H and ^{13}C NMR data of products

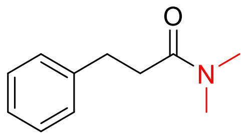

***N*,*N*-Dimethyl 3-phenylpropanamide (2)**:[1] Isolated yield 138 mg (78%)

^{1}H NMR (400 MHz, $CDCl_3$): δ = 7.39–7.14 (m, 5H), 2.98–2.93 (m, 8H), 2.64 (t, *J* = 8.0 Hz, 2H) ppm. ^{13}C NMR (100 MHz, $CDCl_3$): δ = 172.3, 141.6, 128.5, 127.9, 126.2, 37.3, 35.6, 35.4, 31.5 ppm. MS: *m*/*z* = 177 $[M]^+$.

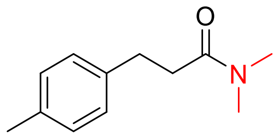

***N*,*N*-Dimethyl 3-(4-methylphenyl)propanamide (3)**:[1] Isolated yield 164 mg (86%)

^{1}H NMR (400 MHz, $CDCl_3$): δ = 7.17–7.02 (m, 4H), 2.99–2.82 (m, 8H), 2.59 (t, *J* = 8.0 Hz, 2H), 2.31 (s, 3H) ppm. ^{13}C NMR (100 MHz, $CDCl_3$): δ = 172.3, 138.4, 135.5, 129.1, 128.3, 37.2, 35.5, 35.4, 30.9, 21.0 ppm. MS: *m*/*z* = 191 $[M]^+$.

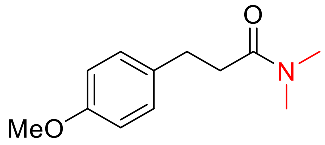

***N*,*N*-Dimethyl 3-(4-methoxyphenyl)propanamide (4)**:[2] Isolated yield 174 mg (84%)

^{1}H NMR (400 MHz, $CDCl_3$): δ = 7.13 (d, *J* = 8.6 Hz, 2H), 6.82 (d, *J* = 8.6 Hz, 2H), 3.78 (s, 3H), 2.98–2.88 (m, 8H), 2.58 (t, *J* = 8.9 Hz, 2H) ppm. ^{13}C NMR (100 MHz, $CDCl_3$): δ = 172.6, 158.1, 133.6, 129.5, 114.0, 55.4, 39.2, 37.3, 35.7, 30.6 ppm. MS: *m*/*z* = 207 $[M]^+$.

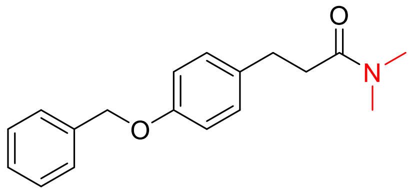

***N*,*N*-Dimethyl 3-(4-(benzyloxy)phenyl)propanamide (5)**: Isolated yield 238 mg (84%)

^{1}H NMR (400 MHz, $CDCl_3$): δ = 7.50–7.30 (m, 5H), 7.21–7.13 (m, 2H), 6.97–6.88 (m, 2H), 5.07 (s, 2H), 3.02–2.85 (m, 8H), 2.61 (t, *J* = 8.0 Hz, 2H) ppm. ^{13}C NMR (100 MHz, $CDCl_3$): δ = 172.4,

157.2, 137.2, 133.8, 129.4, 128.6, 127.9, 127.5, 114.9, 70.1, 37.2, 35.5, 35.5, 30.5 ppm. MS: m/z = 283 $[M]^+$.

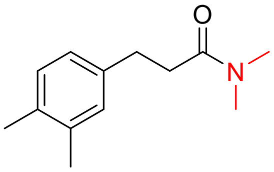

***N*,*N*-Dimethyl 3-(3,4-dimethylphenyl)propanamide (6)**: Isolated yield 170 mg (83%)

^{1}H NMR (400 MHz, $CDCl_3$): δ = 7.17–6.84 (m, 3H), 3.02–2.80 (m, 8H), 2.62 (t, J = 8.0 Hz, 2H), 2.27 (s, 3H), 2.26 (s, 3H) ppm. ^{13}C NMR (100 MHz, $CDCl_3$): δ = 172.4, 138.9, 136.6, 134.2, 129.8, 129.7, 125.7, 37.2, 35.6, 35.4, 30.9, 19.8, 19.3 ppm. MS: m/z = 205 $[M]^+$.

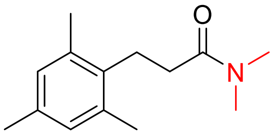

***N*,*N*-Dimethyl 3-(2,4,6-trimethyl)propanamide (7)**: Isolated yield 164 mg (75%)

^{1}H NMR (400 MHz, $CDCl_3$): δ = 6.87 (s, 2H), 3.01–2.92 (m, 8H), 2.45 (t, J = 8.0 Hz, 2H), 2.32 (s, 6H), 2.28 (s, 3H) ppm. ^{13}C NMR (100 MHz, $CDCl_3$): δ = 172.5, 136.2, 135.4, 134.9, 128.9, 37.1, 35.4, 32.6, 24.8, 20.8, 19.7 ppm. MS: m/z = 219 $[M]^+$.

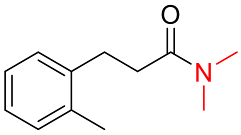

***N*,*N*-Dimethyl 3-(2-methylphenyl)propanamide (8)**:[3] Isolated yield 162 mg (85%)

^{1}H NMR (400 MHz, $CDCl_3$): δ = 7.23–7.09 (m, 4H), 3.00–2.95 (m, 8H), 2.58 (t, J = 8.0 Hz, 2H), 2.35 (s, 3H) ppm. ^{13}C NMR (100 MHz, $CDCl_3$): δ = 172.4, 139.5, 135.9, 130.3, 128.8, 126.3, 126.1, 37.2, 35.5, 33.9, 28.7, 19.3 ppm. MS: m/z = 191 $[M]^+$.

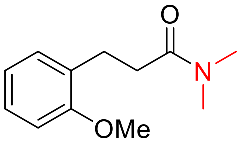

***N*,*N*-Dimethyl 3-(2-methoxyphenyl)propanamide (9)**:[2] Isolated yield 149 mg (72%)

^{1}H NMR (400 MHz, $CDCl_3$): δ = 7.26–7.17 (m, 2H), 6.95–6.83 (m, 2H), 3.84 (s, 3H), 2.99–2.94 (m, 8H), 2.61 (t, J = 8.0 Hz, 2H) ppm. ^{13}C NMR (100 MHz, $CDCl_3$): δ = 172.9, 157.5, 130.2, 129.6, 127.5, 120.5, 110.2, 55.2, 37.2, 35.4, 33.7, 26.7 ppm. MS: m/z = 207 $[M]^+$.

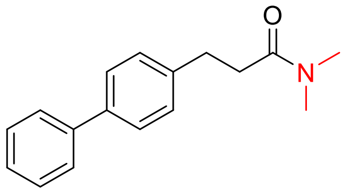

***N*,*N*-Dimethyl 3-([1,1'-biphenyl]-4-yl)propanamide (10)**:[4] Isolated yield 190 mg (75%)
^{1}H NMR (400 MHz, $CDCl_3$): δ = 7.64–7.31 (m, 9H), 3.11–2.94 (m, 8H), 2.68 (t, J = 8.0 Hz, 2H) ppm. ^{13}C NMR (100 MHz, $CDCl_3$): δ = 172.1, 141.0, 140.7, 139.1, 128.9, 128.8, 127.2, 127.1, 127.0, 37.2, 35.5, 35.2, 31.0 ppm. MS: m/z = 253 $[M]^+$.

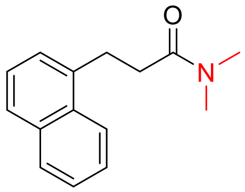

***N*,*N*-Dimethyl 3-(naphthalen-1-yl)propanamide (11)**:[2] Isolated yield 168 mg (74%)
^{1}H NMR (400 MHz, $CDCl_3$): δ = 8.09 (d, J = 8.0 Hz, 1H), 7.93–7.70 (m, 2H), 7.63–7.34 (m, 4H), 3.48 (t, J = 8.0 Hz, 2H), 2.98 (s, 3H), 2.86 (s, 3H), 2.75 (t, J = 8.0 Hz, 2H) ppm. ^{13}C NMR (100 MHz, $CDCl_3$): δ = 172.3, 137.6, 133.9, 131.7, 128.9, 126.9, 126.2, 126.0, 125.7, 125.6, 123.6, 37.1, 35.5, 34.5, 28.5 ppm. MS: m/z = 227 $[M]^+$.

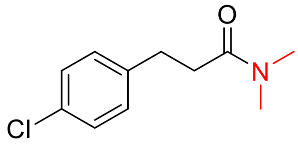

***N*,*N*-Dimethyl 3-(4-chlorophenyl)propanamide (12)**:[3] Isolated yield 135 mg (64%)
^{1}H NMR (400 MHz, $CDCl_3$): δ = 7.24 (d, J = 8.0 Hz, 2H), 7.16 (d, J = 8.0 Hz, 2H), 2.96–2.92 (m, 8H), 2.61 (t, J = 8.0 Hz, 2H) ppm. ^{13}C NMR (100 MHz, $CDCl_3$): δ = 171.9, 139.9, 131.8, 129.8, 128.5, 37.2, 35.5, 35.0, 30.6 ppm. MS: m/z = 211 $[M]^+$.

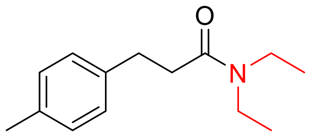

N,N-Diethyl 3-(4-methylphenyl)propanamide (13): Isolated yield 175 mg (80%)

^{1}H NMR (400 MHz, CDCl$_3$): δ = 7.19–7.07 (m, 4H), 3.40 (q, J = 7.1 Hz, 2H), 3.25 (q, J = 7.1 Hz, 2H), 2.96 (t, J = 8.0 Hz, 2H), 2.60 (t, J = 8.0 Hz, 2H), 2.34 (s, 3H), 1.13 (t, J = 7.1 Hz, 6H) ppm. ^{13}C NMR (100 MHz, CDCl$_3$): δ = 171.5, 138.5, 135.6, 129.1, 128.3, 76.7, 41.9, 40.2, 35.3, 31.2, 21.0, 14.3 ppm. MS: m/z = 219 [M]$^+$.

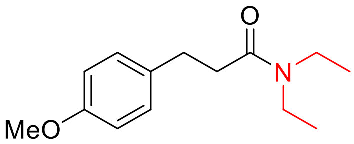

N,N-Diethyl 3-(4-methoxyphenyl)propanamide (14): Isolated yield 193 mg (82%)

^{1}H NMR (400 MHz, CDCl$_3$): δ = 7.13 (d, J = 8.6 Hz, 2H), 6.82 (d, J = 8.6 Hz, 2H), 3.78 (s, 3H), 3.37 (q, J = 7.1 Hz, 2H), 3.21 (q, J = 7.1 Hz, 2H), 2.92 (t, J = 8.0 Hz, 2H), 2.55 (t, J = 8.0 Hz, 2H), 1.17–1.10 (m, 6H) ppm. ^{13}C NMR (100 MHz, CDCl$_3$): δ = 171.4, 157.9, 133.6, 129.4, 113.8, 55.3, 41.9, 40.2, 35.4, 30.8, 14.3, 13.1 ppm. MS: m/z = 235 [M]$^+$.

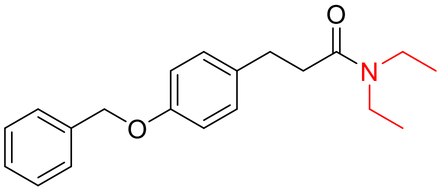

N,N-Diethyl 3-(4-(benzyloxy)phenyl)propanamide (15): Isolated yield 265 mg (85%)

^{1}H NMR (400 MHz, CDCl$_3$): δ = 7.49–7.30 (m, 5H), 7.19–7.15 (m, 2H), 6.97–6.91 (m, 2H), 5.07 (s, 2H), 3.40 (q, J = 7.1 Hz, 2H), 3.23 (q, J = 7.1 Hz, 2H), 3.08–2.87 (m, 2H), 2.70–2.47 (m, 2H), 1.17–1.09 (m, 6H) ppm. ^{13}C NMR (100 MHz, CDCl$_3$): δ = 171.4, 157.2, 137.2, 133.9, 129.4, 128.6, 127.9, 127.4, 114.9, 70.0, 41.9, 40.2, 35.3, 30.9, 14.3, 13.1 ppm. MS: m/z = 311 [M]$^+$.

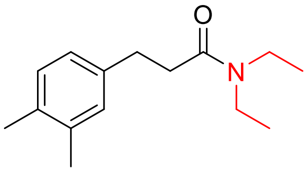

N,N-Diethyl 3-(3,4-dimethylphenyl)propanamide (16): Isolated yield 198 mg (85%)

^{1}H NMR (400 MHz, CDCl$_3$): δ = 7.15–6.91 (m, 3H), 3.41 (q, J = 8.0 Hz, 2H), 3.26 (q, J = 8.0 Hz, 2H), 3.06–2.85 (m, 2H), 2.71–2.51 (m, 2H), 2.26 (s, 3H), 2.25 (s, 3H), 1.14 (t, J = 7.1 Hz, 6H)

ppm. ^{13}C NMR (100 MHz, $CDCl_3$): δ = 171.5, 139.0, 136.6, 134.2, 129.8, 129.7, 125.7, 41.9, 40.2, 35.4, 31.2, 19.7, 19.3, 14.3, 13.1 ppm. MS: m/z = 233 $[M]^+$.

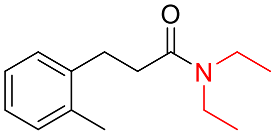

***N,N*-Diethyl 3-(2-methylphenyl)propanamide (17)**: Isolated yield 191 mg (87%)

^1H NMR (400 MHz, $CDCl_3$): δ = 7.20–7.11 (m, 4H), 3.41 (q, J = 7.1 Hz, 2H), 3.25 (q, J = 7.1 Hz, 2H), 3.00 (t, J = 8.0 Hz, 2H), 2.57 (t, J = 8.0 Hz, 2H), 2.36 (s, 3H), 1.18–1.10 (m, 6H) ppm. ^{13}C NMR (100 MHz, $CDCl_3$): δ = 171.4, 139.7, 135.9, 130.3, 128.9, 126.3, 126.1, 41.9, 40.3, 33.7, 28.9, 19.3, 14.3, 13.1 ppm. MS: m/z = 219 $[M]^+$.

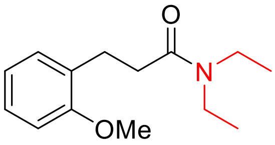

***N,N*-Diethyl 3-(2-methoxyphenyl)propanamide (18)**: Isolated yield 179 mg (76%)

^1H NMR (400 MHz, $CDCl_3$): δ = 7.24–7.19 (m, 2H), 6.95–6.85 (m, 2H), 3.85 (s, 3H), 3.40 (q, J = 7.1 Hz, 2H), 3.29 (q, J = 7.1 Hz, 2H), 3.04–2.87 (m, 2H), 2.71–2.54 (m, 2H), 1.17–1.10 (m, 6H) ppm. ^{13}C NMR (100 MHz, $CDCl_3$): δ = 172.0, 157.5, 130.3, 129.7, 127.4, 120.5, 110.2, 55.2, 41.9, 40.1, 33.4, 27.0, 14.3, 13.1 ppm. MS: m/z = 235 $[M]^+$.

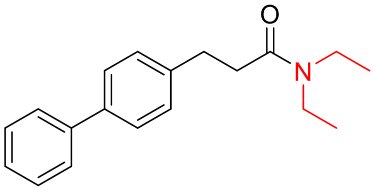

***N,N*-Diethyl 3-([1,1'-biphenyl]-4-yl)propanamide (19)**: Isolated yield 219 mg (78%)

^1H NMR (400 MHz, $CDCl_3$): δ = 7.70–7.31 (m, 9H), 3.42 (q, J = 7.1 Hz, 2H), 3.27 (q, J = 7.2 Hz, 2H), 3.07 (t, J = 8.0 Hz, 2H), 2.67 (t, J = 8.0 Hz, 2H), 1.19–1.11 (m, 6H). ^{13}C NMR (100 MHz, $CDCl_3$): δ = 171.2, 141.0, 140.7, 139.0, 128.9, 128.7, 127.2, 127.1, 127.0, 41.9, 40.3, 35.0, 31.3, 14.3, 13.1 ppm. MS: m/z = 281 $[M]^+$.

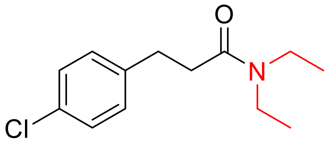

N,N-Diethyl 3-(4-chlorophenyl)propanamide (20): Isolated yield 175 mg (73%)

^{1}H NMR (400 MHz, $CDCl_3$): δ = 7.25 (d, J = 8.0 Hz, 2H), 7.16 (d, J = 8.0 Hz, 2H), 3.38 (q, J = 7.1 Hz, 2H), 3.23 (q, J = 7.2 Hz, 2H), 2.97 (t, J = 8.0 Hz, 2H), 2.58 (t, J = 8.0 Hz, 2H), 1.11 (t, J = 7.1 Hz, 6H) ppm. ^{13}C NMR (100 MHz, $CDCl_3$): δ = 170.9, 140.0, 131.8, 129.9, 128.5, 41.9, 40.3, 34.8, 30.8, 14.3, 13.1 ppm. MS: m/z = 239 $[M]^+$.

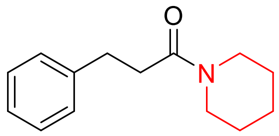

3-Phenyl-1-(piperidin-1-yl)propan-1-one (21):[3] Isolated yield 174 mg (80%)

^{1}H NMR (400 MHz, $CDCl_3$): δ = 7.41–7.07 (m, 5H), 3.62–3.31 (m, 4H), 3.09–2.91 (m, 2H), 2.77–2.56 (m, 2H), 1.71–1.40 (m, 6H) ppm. ^{13}C NMR (100 MHz, $CDCl_3$): δ = 170.5, 141.5, 128.5, 128.4, 126.1, 46.7, 42.6, 35.2, 31.6, 26.4, 25.5, 24.5 ppm. MS: m/z = 217 $[M]^+$.

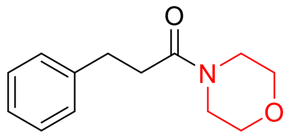

1-Morpholino-3-phenylpropan-1-one (22):[2] Isolated yield 171 mg (78%)

^{1}H NMR (400 MHz, $CDCl_3$): δ = 7.37–7.19 (m, 5H), 3.64 (brs, 4H), 3.56–3.50 (m, 2H), 3.40–3.34 (m, 2H), 2.95 (t, J = 8.0 Hz, 2H), 2.64 (t, J = 8.0 Hz, 2H) ppm. ^{13}C NMR (100 MHz, $CDCl_3$): δ = 170.9, 141.1, 128.5, 128.4, 126.3, 66.9, 66.5, 45.9, 41.9, 34.8, 31.5 ppm. MS: m/z = 219 $[M]^+$.

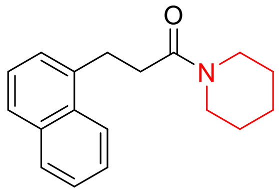

3-(Naphthalen-1-yl)-1-(piperidin-1-yl)propan-1-one (23): Isolated yield 215 mg (78%)

^{1}H NMR (400 MHz, $CDCl_3$): δ = 8.06 (d, J = 8.2 Hz, 1H), 7.86 (dd, J = 7.9, 1.5 Hz, 1H), 7.73 (dd, J = 7.6, 1.8 Hz, 1H), 7.55–7.45 (m, 2H), 7.43–7.32 (m, 2H), 3.57 (brs, 2H), 3.48–3.40 (m, 2H), 3.24 (brs, 2H), 2.83–2.65 (m, 2H), 1.73–1.36 (m, 6H) ppm. ^{13}C NMR (100 MHz, $CDCl_3$): δ = 170.6, 137.6, 133.9, 131.7, 128.8, 126.9, 126.2, 126.0, 125.6, 125.5, 123.6, 46.6, 42.7, 34.3, 28.7, 26.3, 25.5, 24.5 ppm. MS: m/z = 267 $[M]^+$.

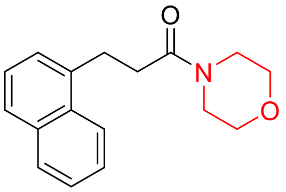

1-Morpholino-3-(naphthalen-1-yl)propan-1-one (24):[5] Isolated yield 215 mg (80%)

^{1}H NMR (400 MHz, $CDCl_3$): δ = 8.07 (d, J = 8.2 Hz, 1H), 7.89 (dd, J = 8.0, 1.5 Hz, 1H), 7.77 (dd, J = 8.0, 1.5 Hz, 1H), 7.58–7.48 (m, 2H), 7.47–7.36 (m, 2H), 3.66–3.56 (m, 4H), 3.52–3.45 (m, 2H), 3.37–3.31 (m, 2H), 3.25–3.19 (m, 2H), 2.81–2.74 (m, 2H) ppm. ^{13}C NMR (100 MHz, $CDCl_3$): δ = 171.0, 137.1, 133.9, 131.6, 128.9, 127.1, 126.4, 126.1, 125.6, 125.6, 123.5, 66.8, 66.3, 45.9, 41.9, 33.9, 28.7 ppm. MS: m/z = 269 $[M]^+$.

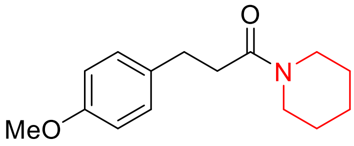

3-(4-Methoxyphenyl)-1-(piperidin-1-yl)propan-1-one (25):[6] Isolated yield 187 mg (85%)

^{1}H NMR (400 MHz, $CDCl_3$): δ = 7.15 (d, J = 7.2 Hz, 2H), 6.84 (d, J = 7.2 Hz, 2H), 3.79 (s, 3H), 3.58–3.52 (m, 2H), 3.35–3.29 (m, 2H), 3.02–2.77 (m, 2H), 2.69–2.47 (m, 2H), 1.69–1.40 (m, 6H) ppm. ^{13}C NMR (100 MHz, $CDCl_3$): δ = 170.5, 157.9, 133.5, 129.3, 113.9, 55.3, 46.6, 42.7, 35.4, 30.7, 26.4, 25.6, 24.5 ppm. MS: m/z = 247 $[M]^+$.

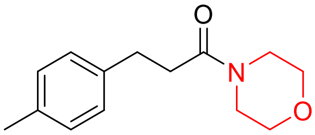

3-(4-Methylphenyl)-1-morpholinopropan-1-one (26):[7] Isolated yield 187 mg (80%)

^{1}H NMR (400 MHz, $CDCl_3$): δ = 7.13–7.09 (m, 4H), 3.64 (brs, 4H), 3.55–3.43 (m, 2H), 3.40–3.34 (m, 2H), 3.03–2.82 (m, 2H), 2.67–2.49 (m, 2H), 2.34 (s, 3H) ppm. ^{13}C NMR (100 MHz, $CDCl_3$): δ = 171.1, 137.9, 135.8, 129.2, 128.3, 66.9, 66.5, 46.0, 41.9, 35.0, 31.1, 21.0 ppm. MS: m/z = 233 $[M]^+$.

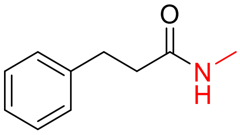

***N*-Methyl 3-phenylpropanamide (27)**:[2] Isolated yield 122 mg (75%)

^{1}H NMR (400 MHz, $CDCl_3$): δ = 7.35–7.27 (m, 2H), 7.24–7.19 (m, 3H), 5.55 (s, 1H), 2.98 (t, J = 8.0 Hz, 2H), 2.79 (s, 1.5H), 2.77 (s, 1.5H), 2.49 (t, J = 8.0 Hz, 2H) ppm. ^{13}C NMR (100 MHz, $CDCl_3$): δ = 172.8, 140.9, 128.5, 128.3, 126.2, 38.4, 31.8, 26.3 ppm. MS: m/z = 163 $[M]^+$.

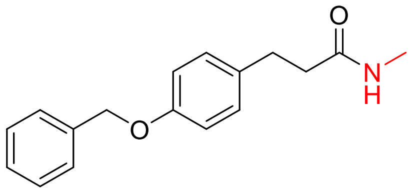

***N*-Methyl 3-(4-(benzyloxy)phenyl)propanamide (28)**: Isolated yield 219 mg (80%)

^{1}H NMR (400 MHz, $CDCl_3$): δ = 7.48–7.31 (m, 5H), 7.15–7.10 (m, 2H), 6.95–6.89 (m, 2H), 5.48 (brs, 1H), 5.06 (s, 2H), 2.93 (t, J = 7.7 Hz, 2H), 2.79 (s, 1.5H), 2.77 (s, 1.5H), 2.46 (t, J = 7.7 Hz, 2H) ppm. ^{13}C NMR (100 MHz, $CDCl_3$): δ = 173.9, 157.3, 137.1, 133.3, 129.3, 128.6, 127.9, 127.5, 114.9, 70.0, 38.7, 30.9, 26.3 ppm. MS: m/z = 269 $[M]^+$.

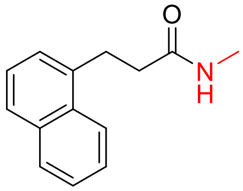

***N*-Methyl 3-(naphthalen-1-yl)propanamide (29)**: Isolated yield 154 mg (72%)

^{1}H NMR (400 MHz, $CDCl_3$): δ = 8.06 (d, J = 8.0 Hz, 1H), 7.88 (d, J = 8.0, 1.8 Hz, 1H), 7.74 (d, J = 8.0 Hz, 1H), 7.57–7.47 (m, 2H), 7.43–7.32 (m, 2H), 5.55 (brs, 1H), 3.45 (t, J = 8.0 Hz, 2H), 2.77 (s, 1.5H), 2.75 (s, 1.5H), 2.60 (t, J = 8.0 Hz, 2H) ppm. ^{13}C NMR (100 MHz, $CDCl_3$): δ = 172.9, 136.9, 133.8, 131.5, 128.8, 127.1, 126.1, 126.1, 125.6, 123.5, 37.6, 28.9, 26.3 ppm. MS: m/z = 213 $[M]^+$.

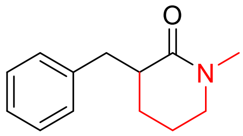

3-Benzyl-1-methylpiperidin-2-one (30):[8] Isolated yield 142 mg (70%)

^{1}H NMR (400 MHz, $CDCl_3$): δ = 7.36–7.27 (m, 2H), 7.26–7.18 (m, 3H), 3.51–3.42 (m, 1H), 3.32–3.22 (m, 2H), 2.98 (s, 3H), 2.71–2.50 (m, 2H), 1.91–1.65 (m, 3H), 1.52–1.41 (m, 1H) ppm. ^{13}C NMR (100 MHz, $CDCl_3$): δ = 172.1, 140.2, 129.2, 128.3, 126.1, 50.2, 43.3, 37.9, 35.0, 25.8, 21.4 ppm. MS: m/z = 203 $[M]^+$.

References:

1. W. Yao, X. Ma, L. Guo, X. Jia, A. Hu and Z. Huang, *Tetrahedron Lett*., 2016, **57**, 2919–2921.
2. Y. K. Jang, T. Krückel, M. Rueping and O. El-Sepelgy, *Org Lett*., 2018, **20**, 7779−7783.
3. X. Zhou, Gu. Zhang, B. Gao and H. Huang, *Org. Lett*., 2018, **20**, 2208−2212.
4. T. Kuwahara, T. Fukuyama and I. Ryu, *RSC Adv*., 2013, **3**, 13702–13704.
5. T. J. Coxon, M. Fernández, J. Barwick-Silk, A. I. McKay, L. E. Britton, A. S. Weller and M. C. Willis, *J. Am. Chem. Soc*., 2017, **139**, 10142−10149.
6. X. Li, F. Lin, K. Huang, J. Wei, X. Li, X. Wang, X. Geng and N. Jiao, *Angew. Chem. Int. Ed*., 2017, **56**, 12307−12311.
7. J. Rana, V. Gupta and E. Balaraman, *Dalton Trans*., 2019, **48**, 7094–7099.
8. D. Katayev, J. Václavík, F. Brüning, B. Commare and A. Togni, *Chem. Commun*., 2016, **52**, 4049–4052.

Copies of ^{1}H and ^{13}C NMR spectra

N,*N*-Dimethyl 3-phenylpropanamide (2)

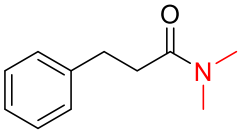

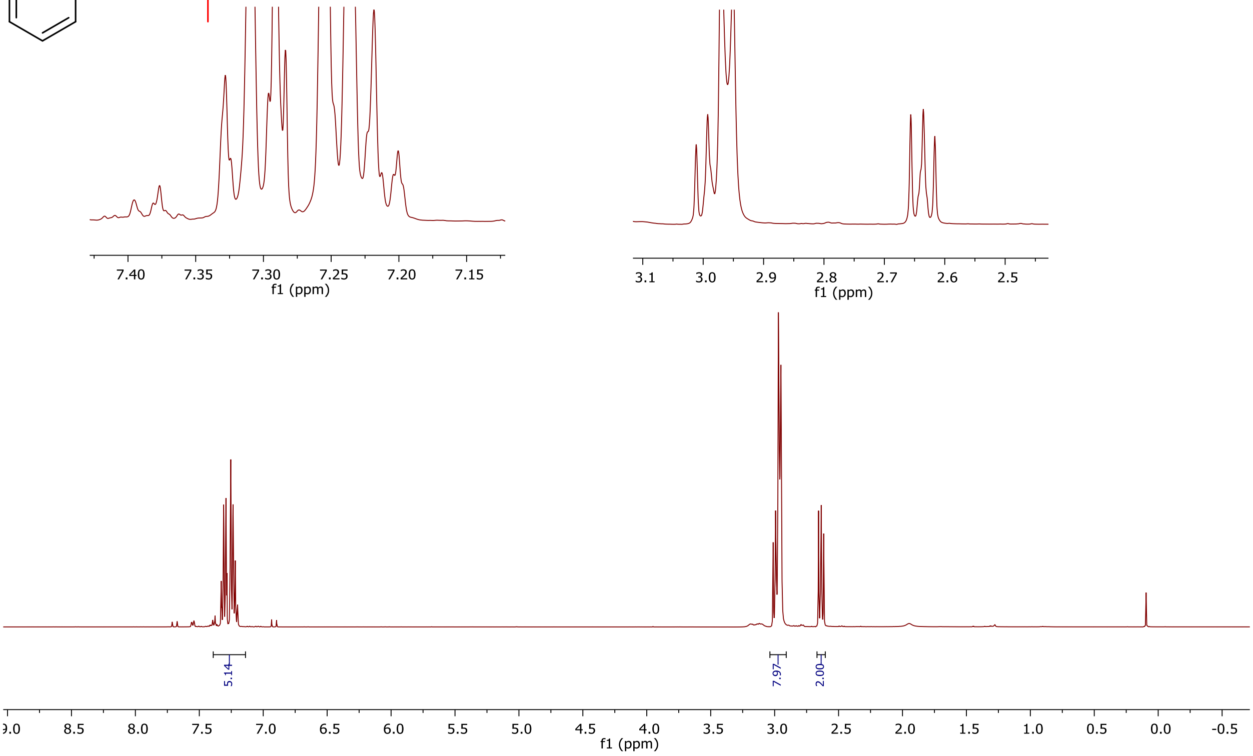

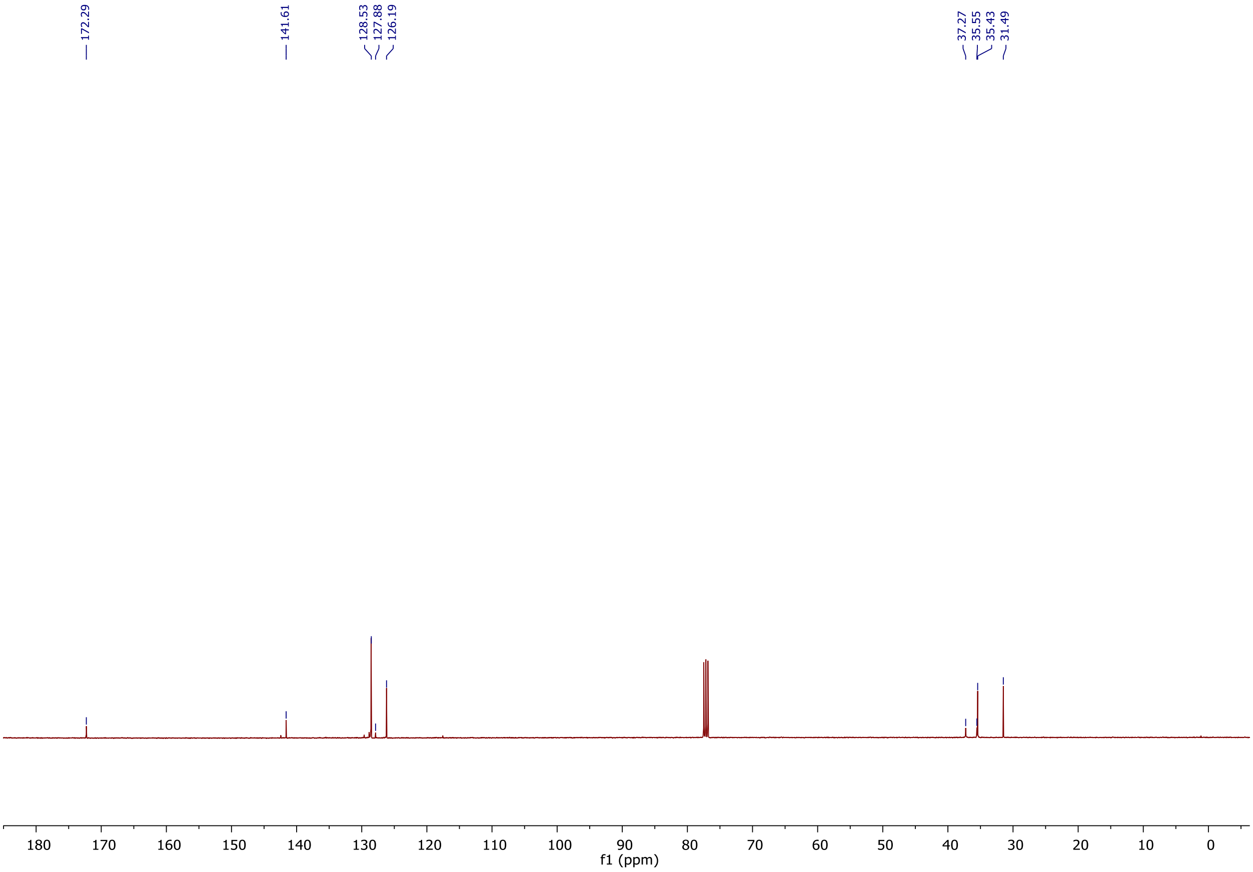

***N*,*N*-Dimethyl 3-(4-methylphenyl)propanamide (3)**

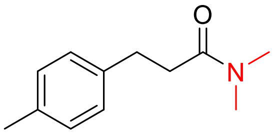

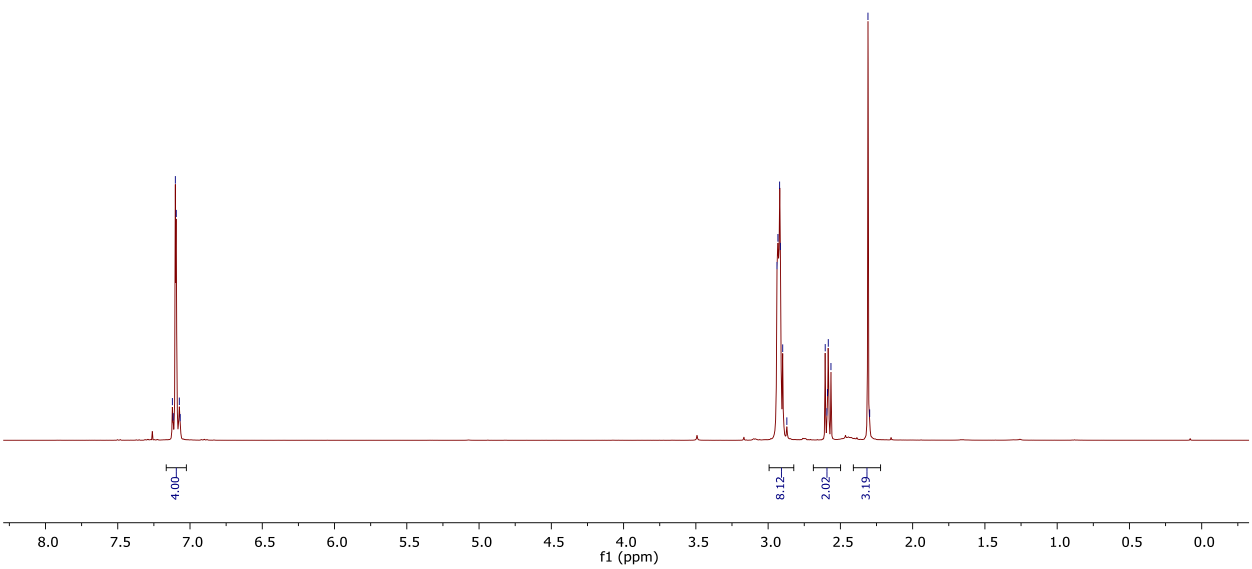

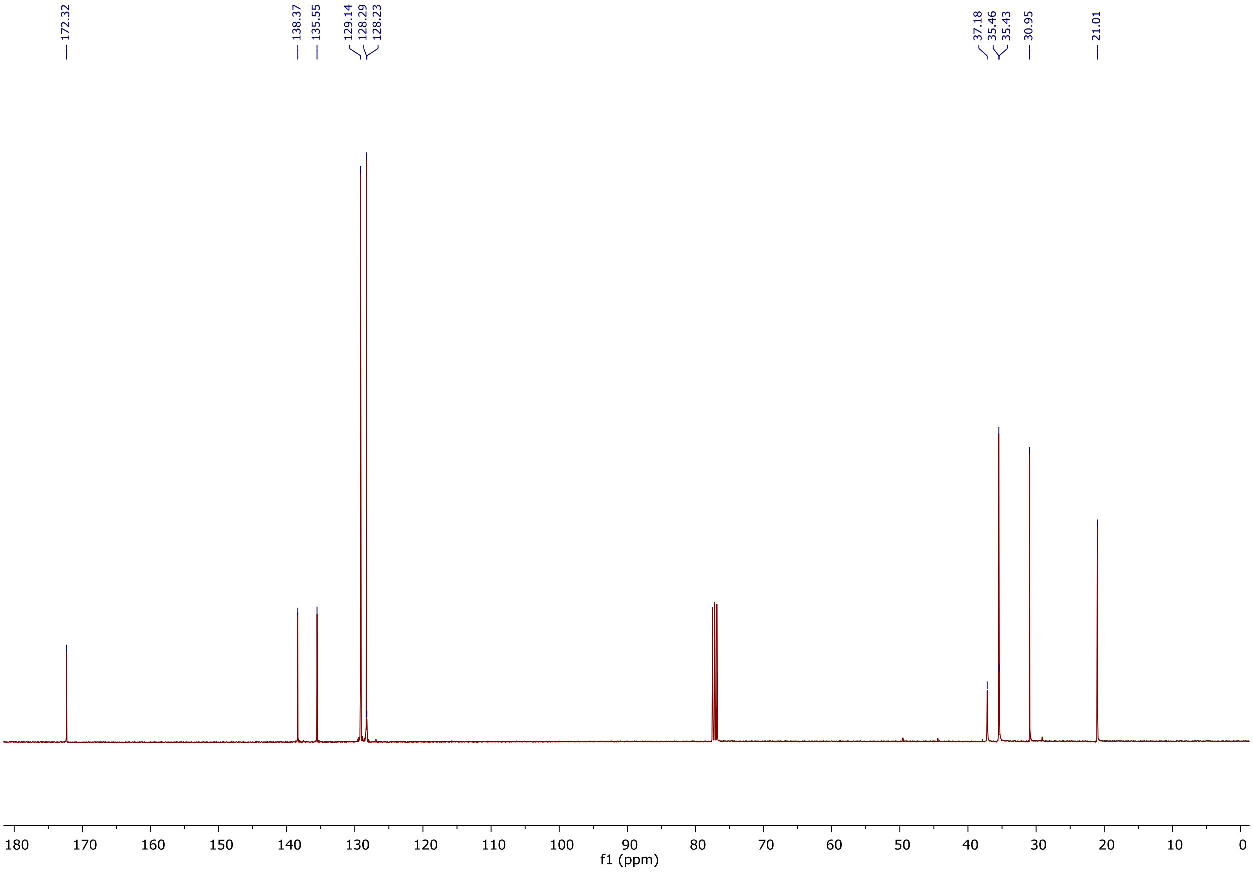

***N*,*N*-Dimethyl 3-(4-methoxyphenyl)propanamide (4)**

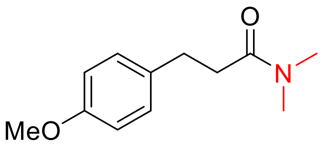

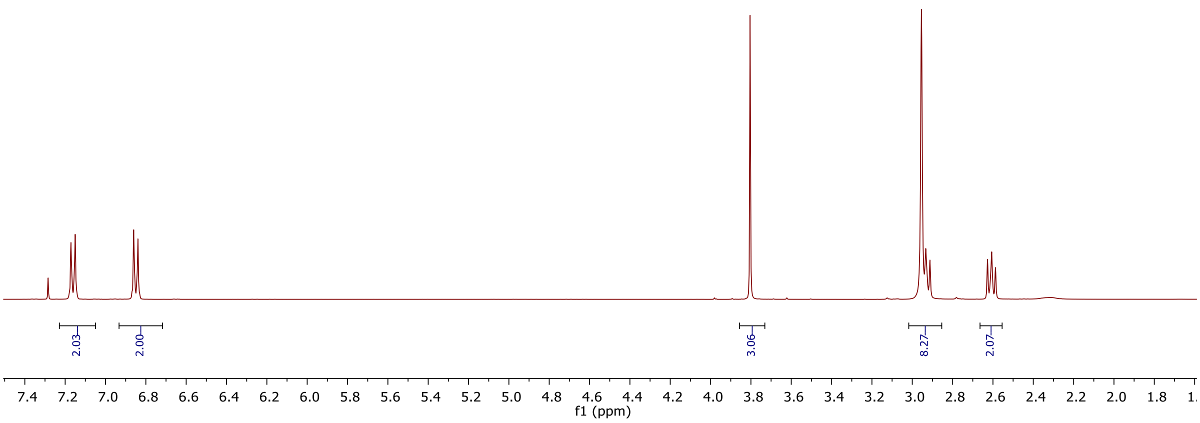

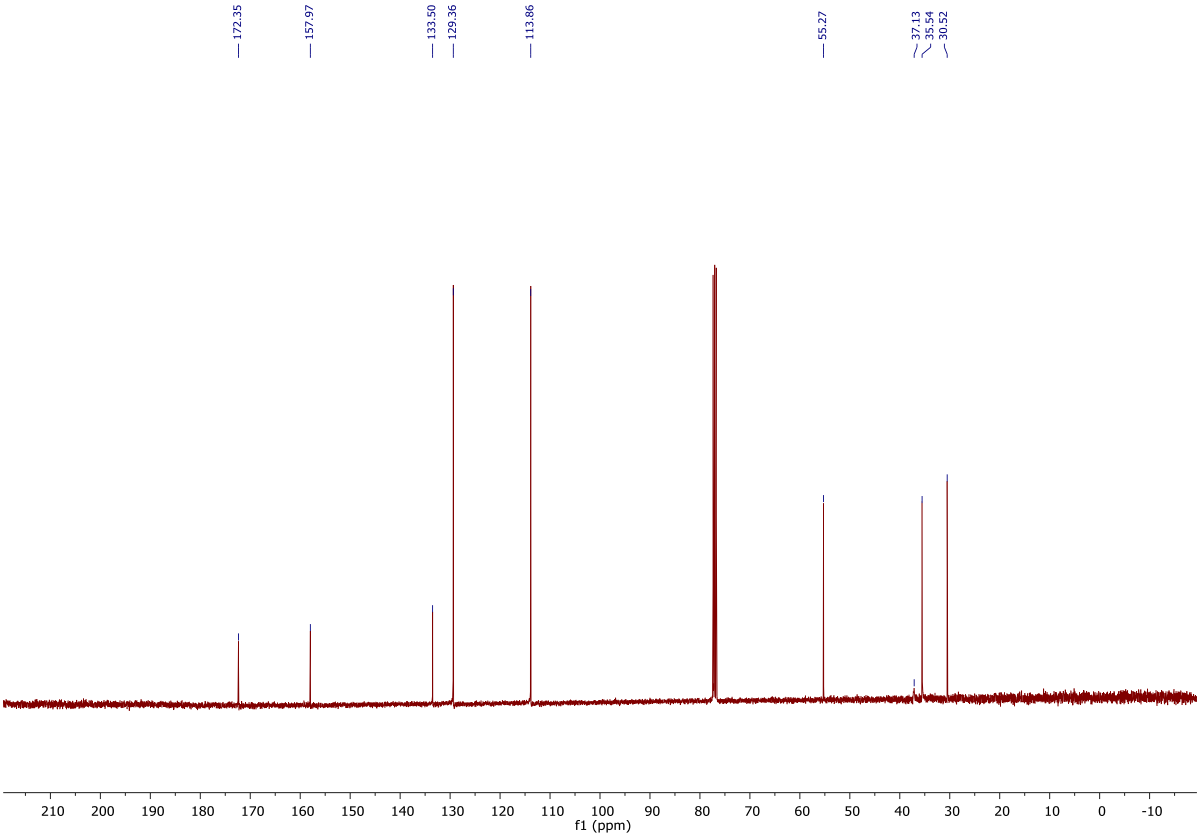

***N*,*N*-Dimethyl 3-(4-(benzyloxy)phenyl)propanamide (5)**

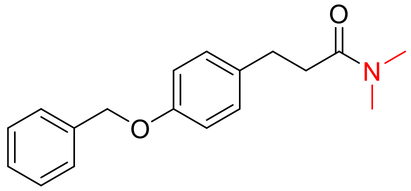

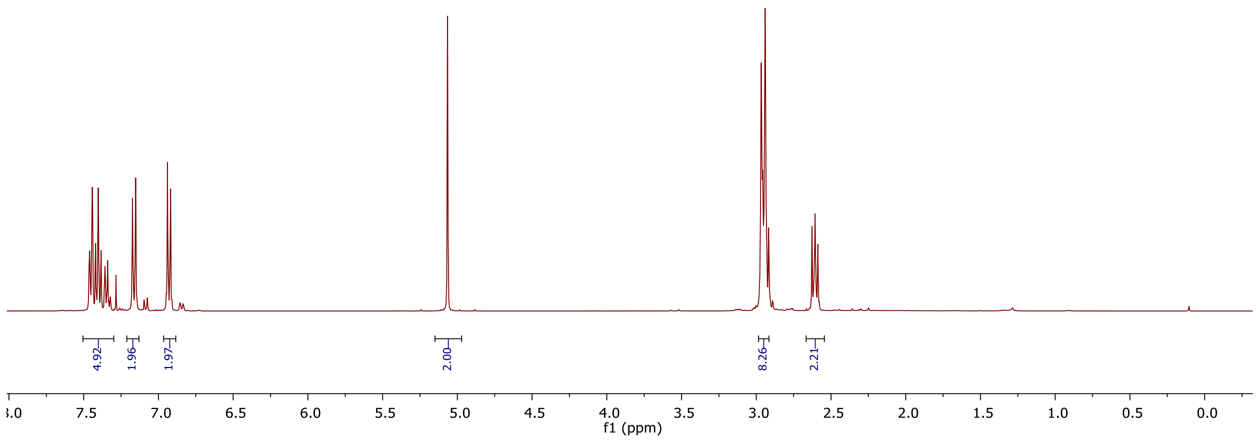

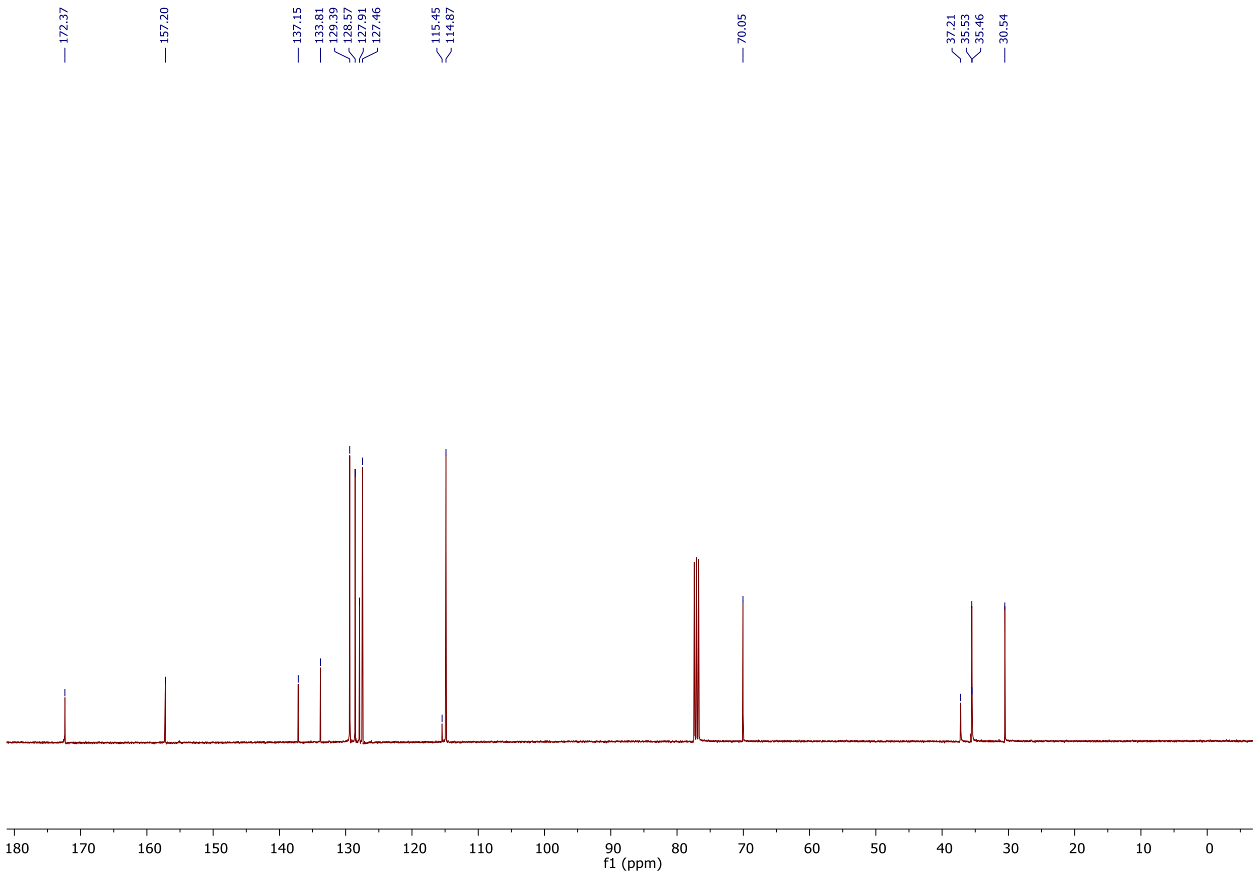

***N*,*N*-Dimethyl 3-(3,4-dimethylphenyl)propanamide (6)**

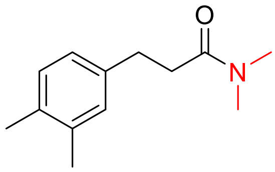

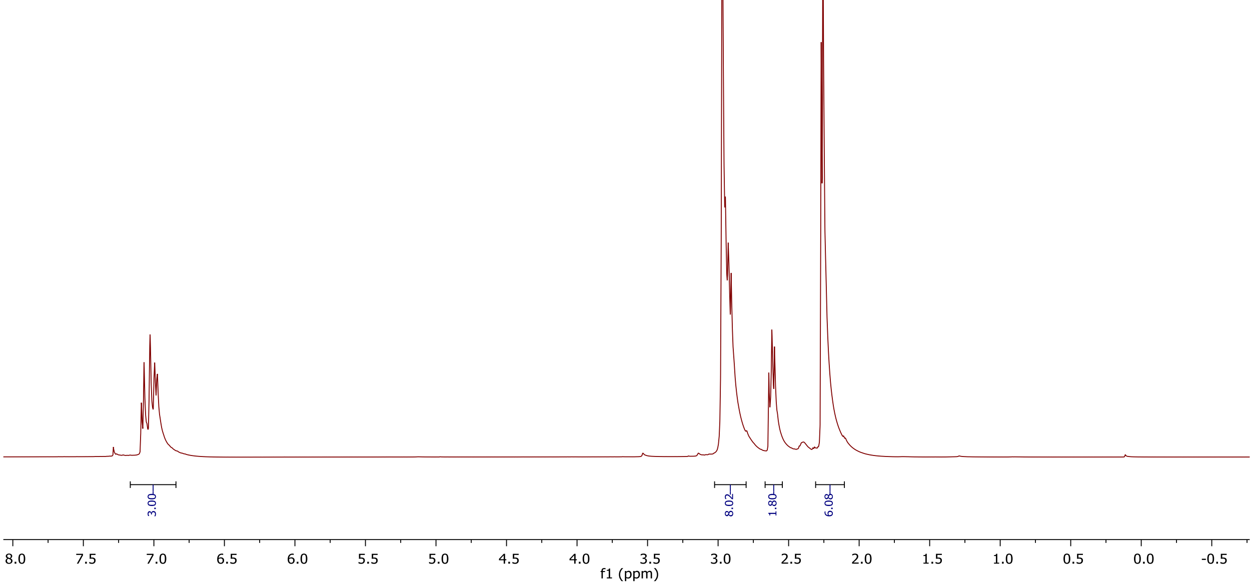

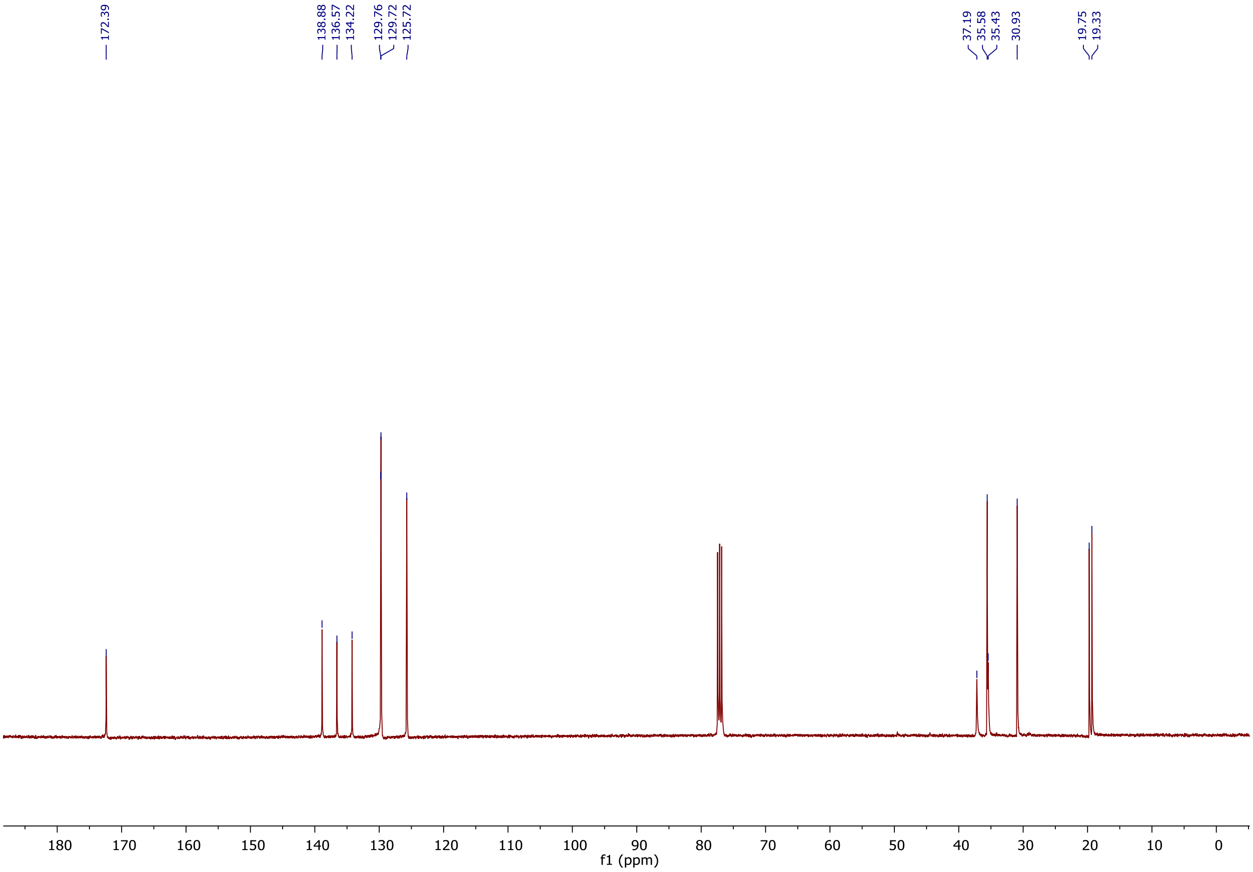

N,N-Dimethyl 3-(2,4,6-trimethyl)propanamide (7)

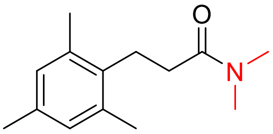

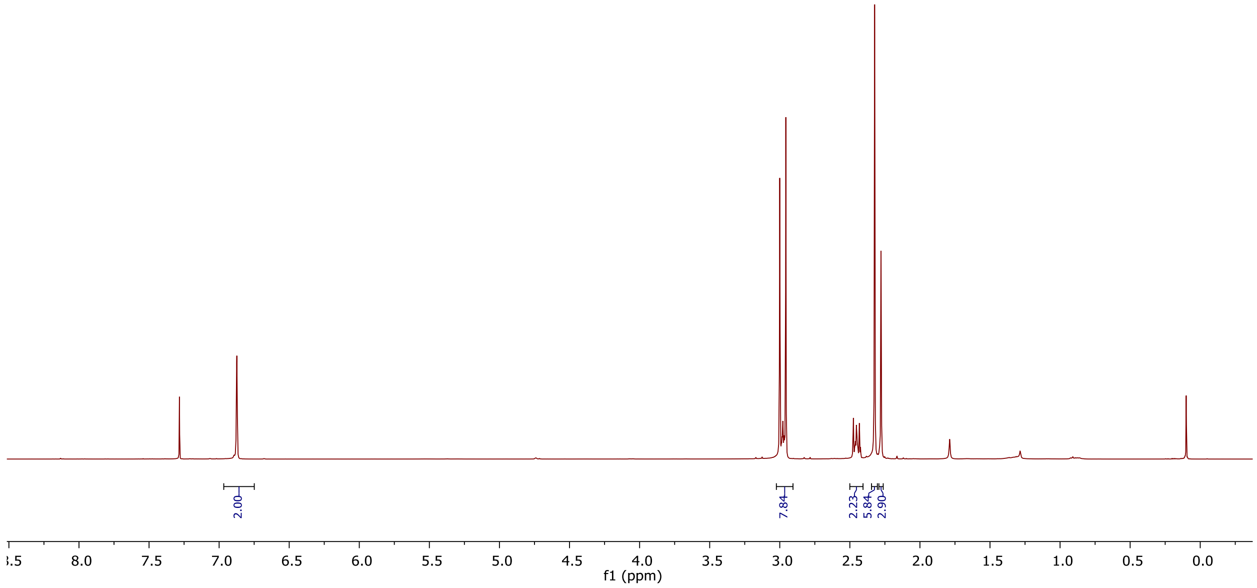

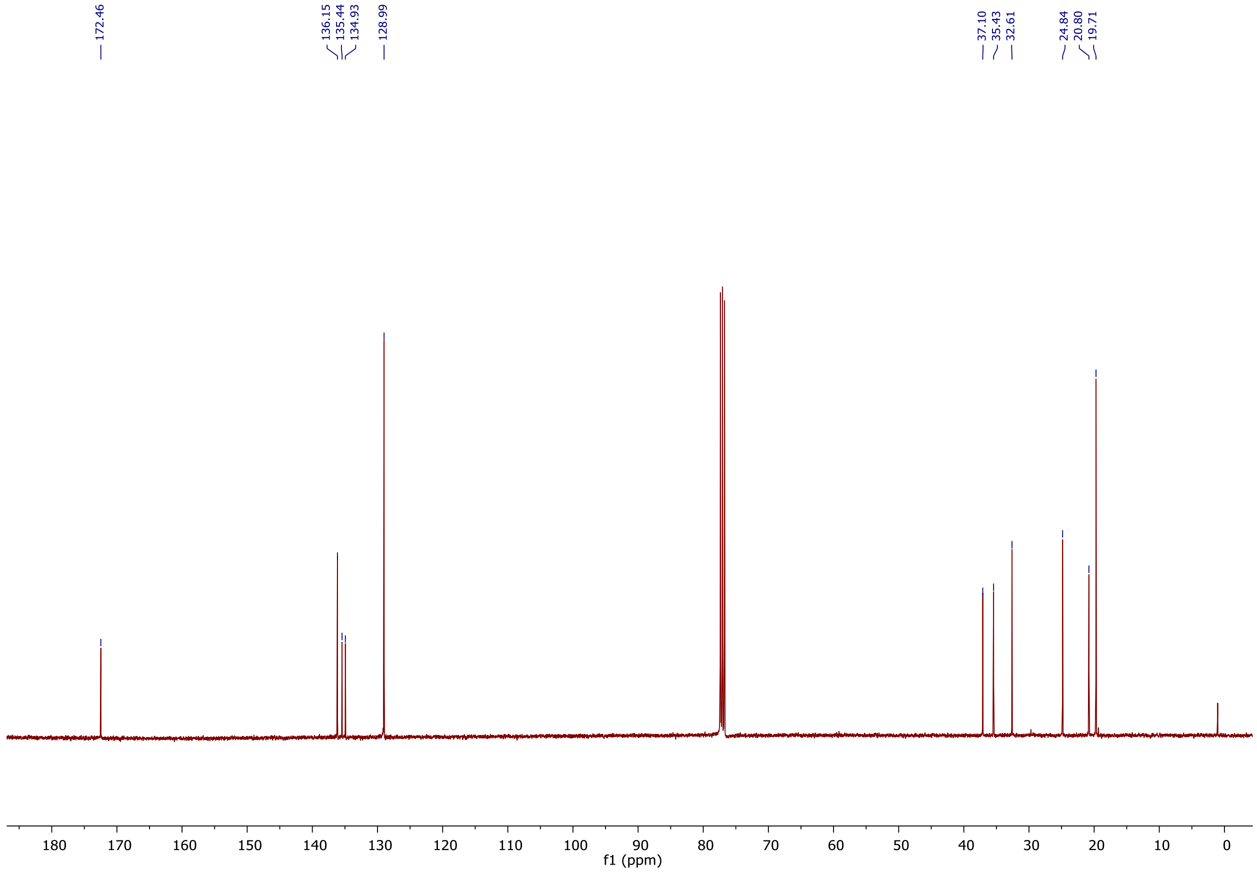

***N*,*N*-Dimethyl 3-(2-methylphenyl)propanamide (8)**

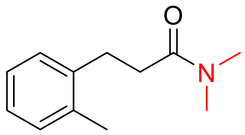

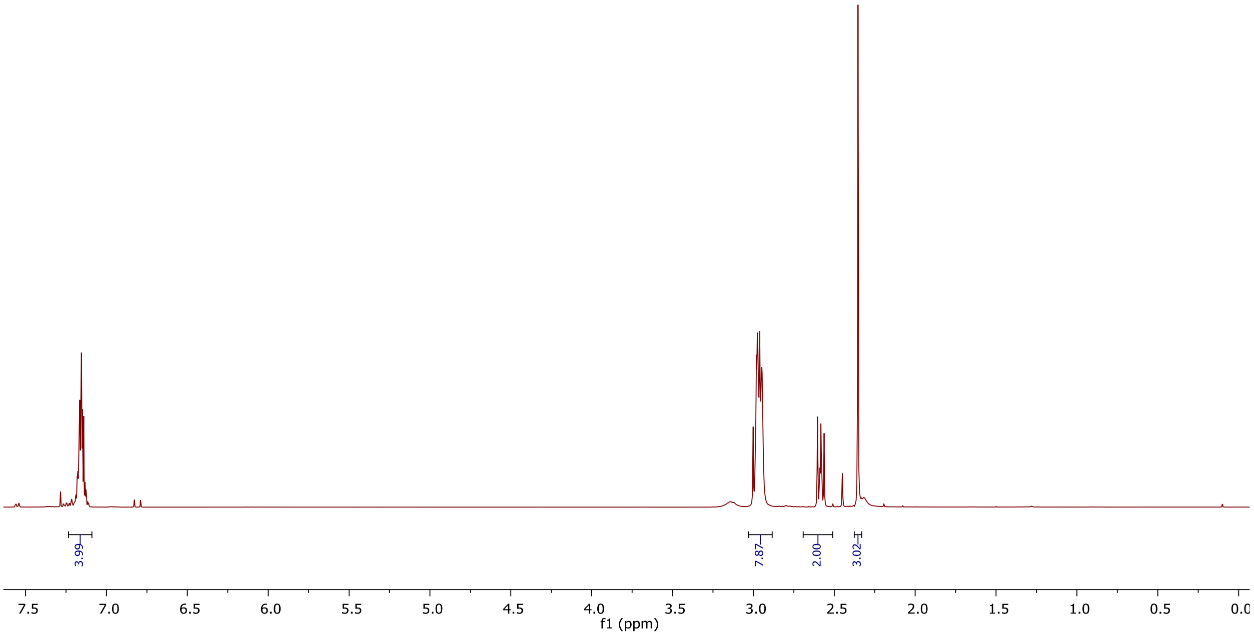

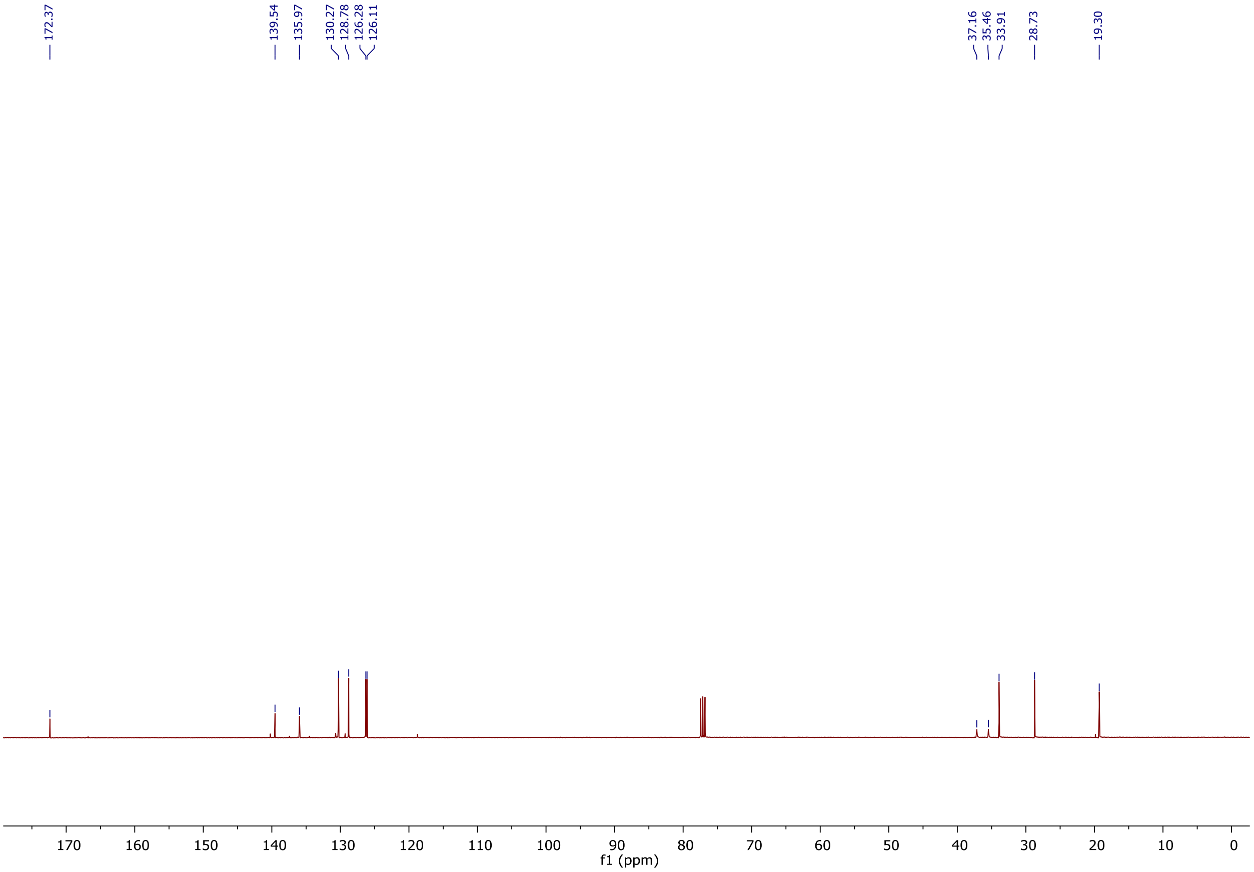

_N,N_-Dimethyl 3-(2-methoxyphenyl)propanamide (9)

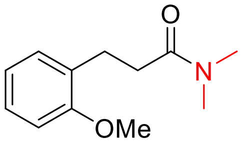

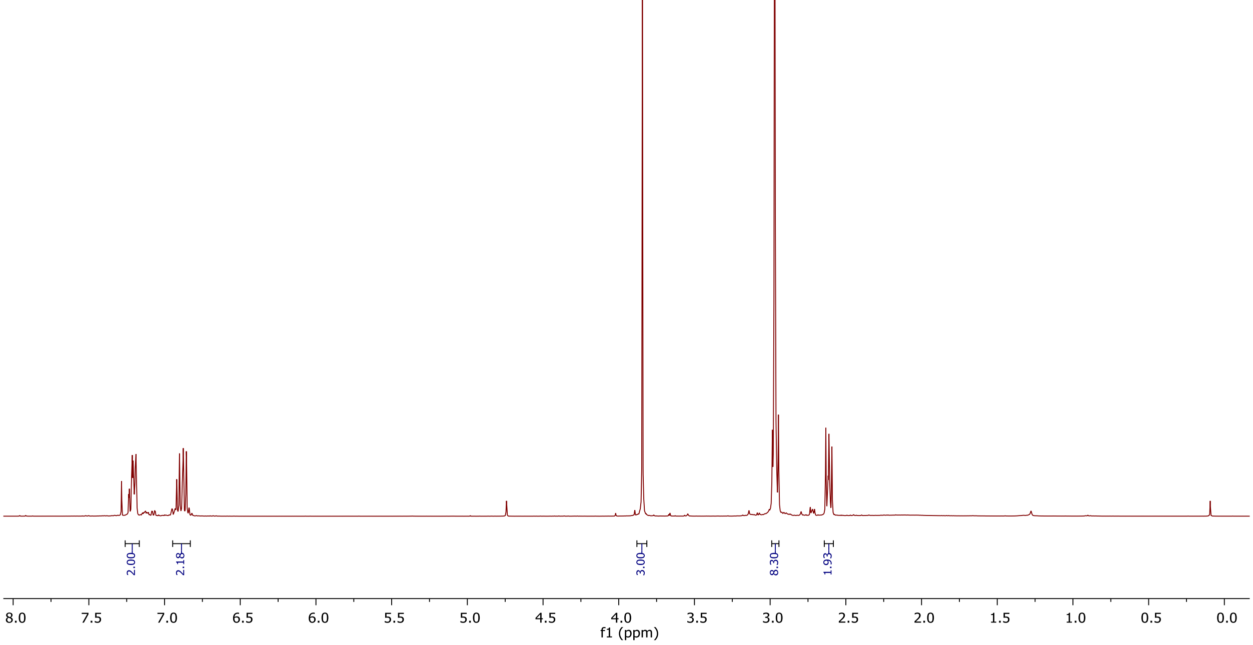

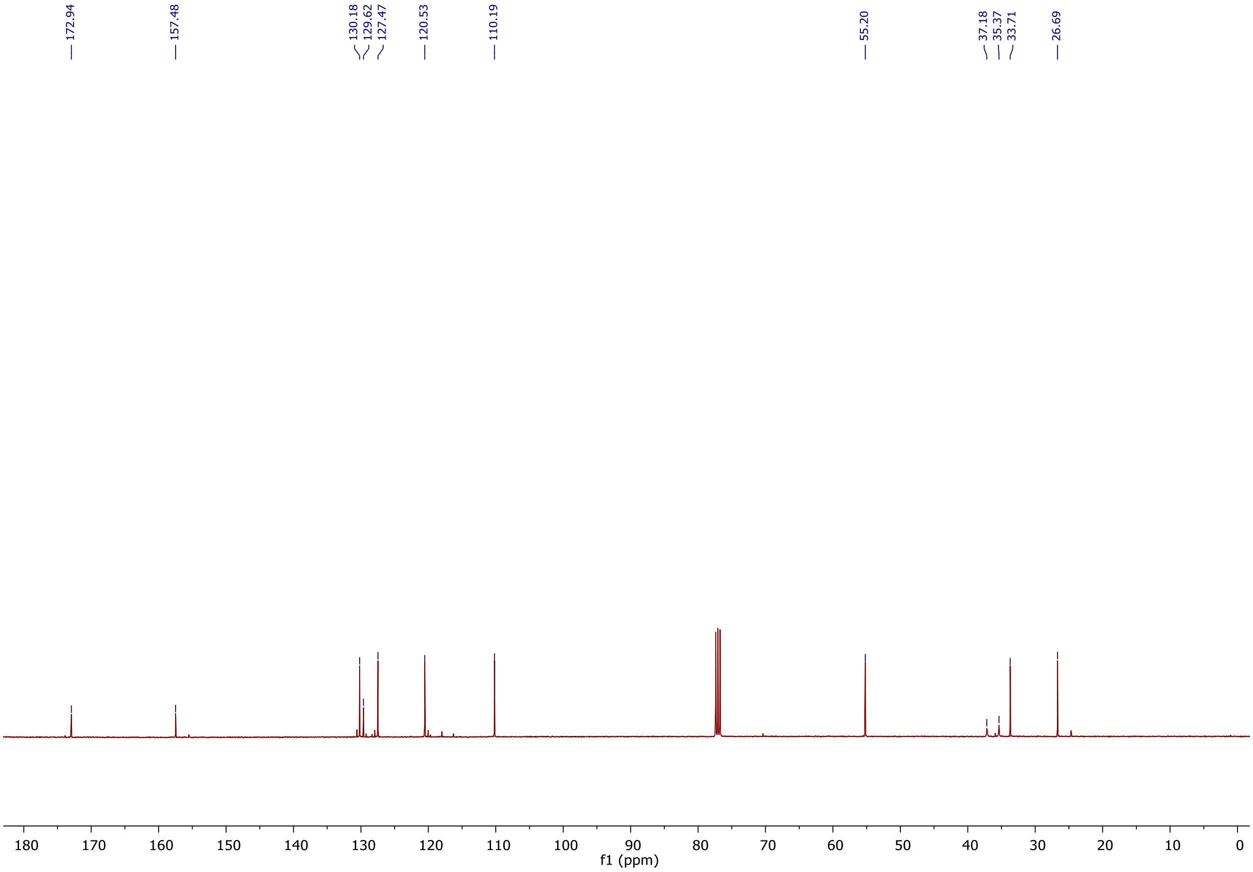

***N*,*N*-Dimethyl 3-([1,1'-biphenyl]-4-yl)propanamide (10)**

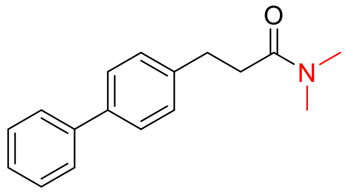

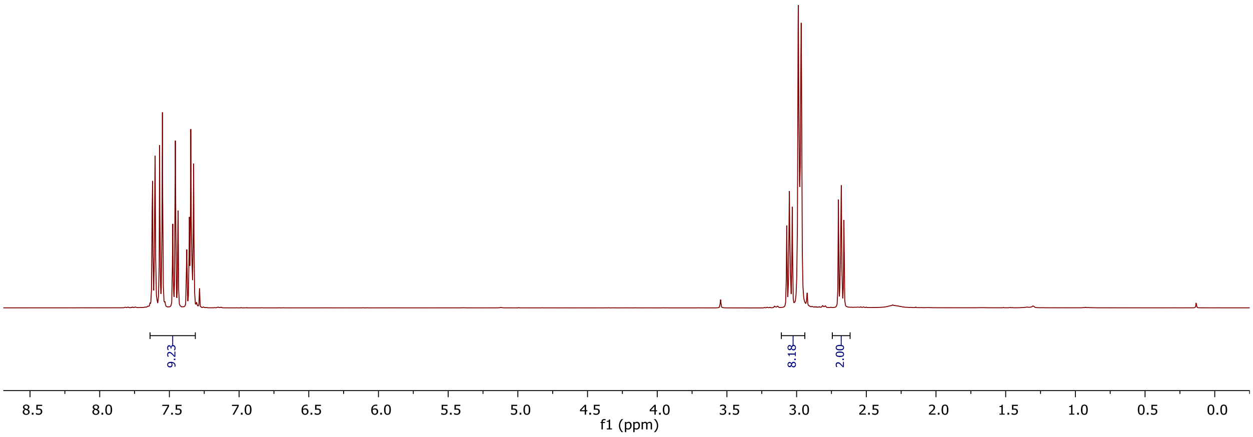

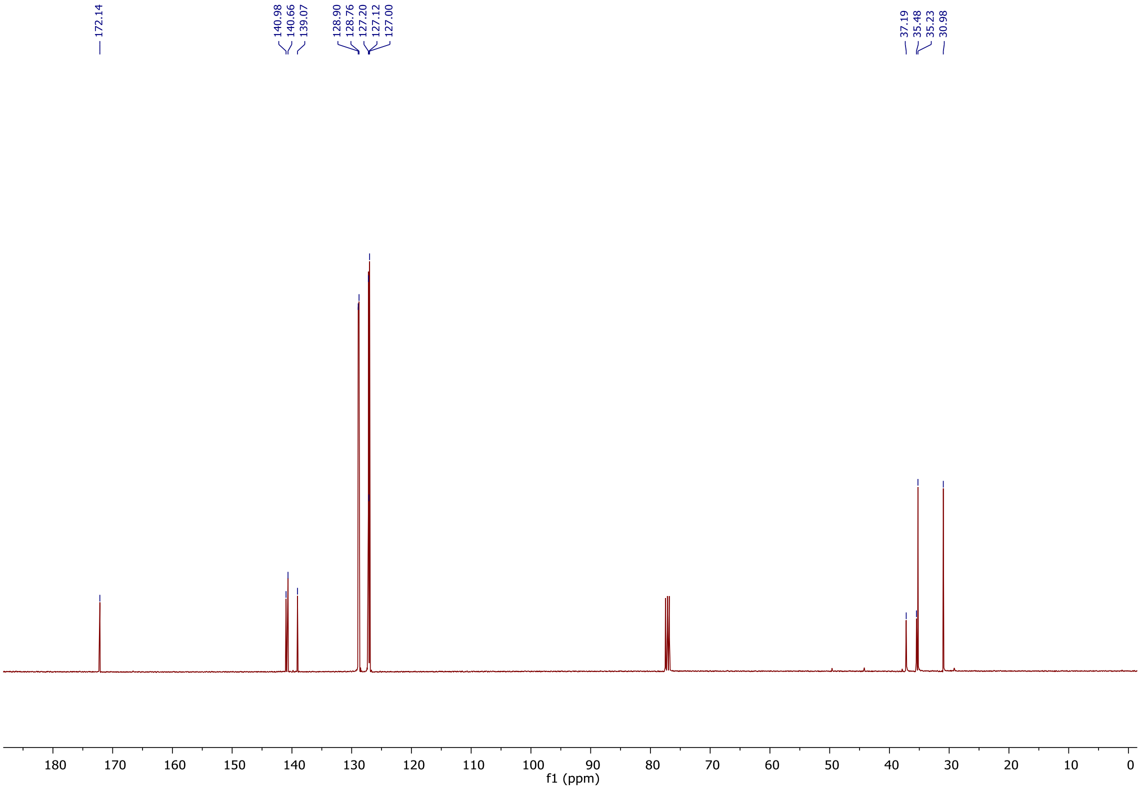

N,*N*-Dimethyl 3-(naphthalen-1-yl)propanamide (11)

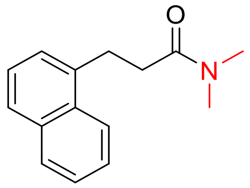

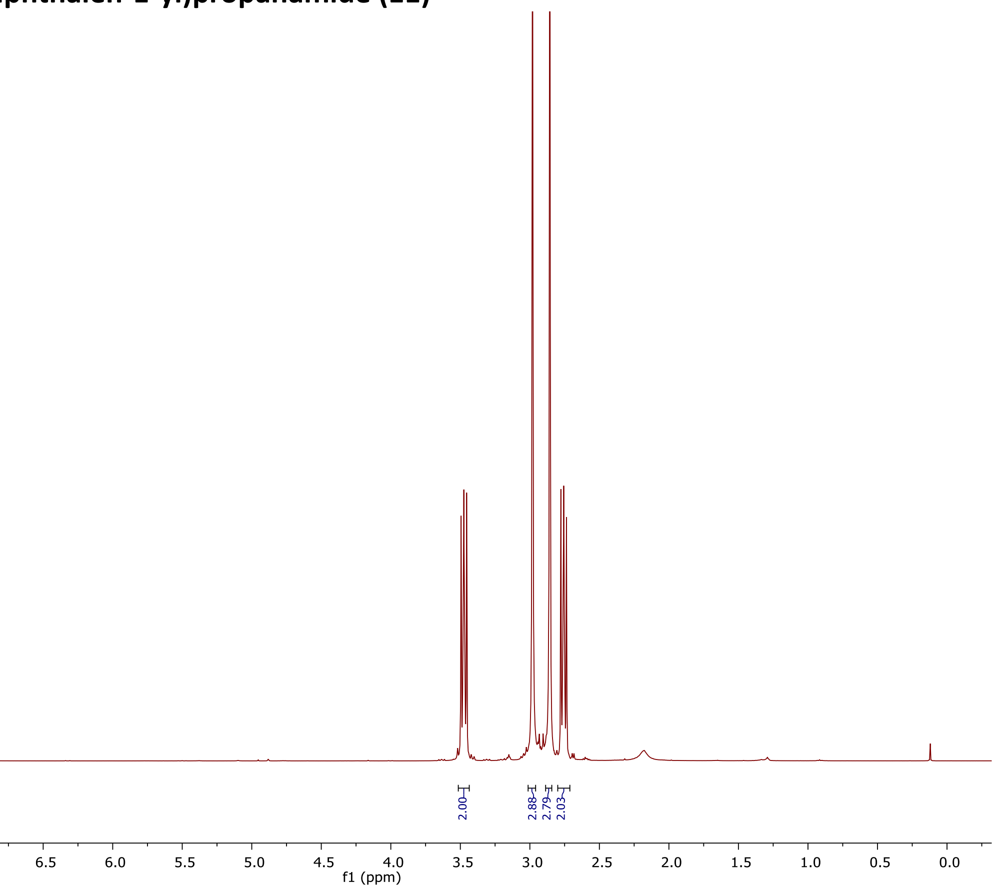

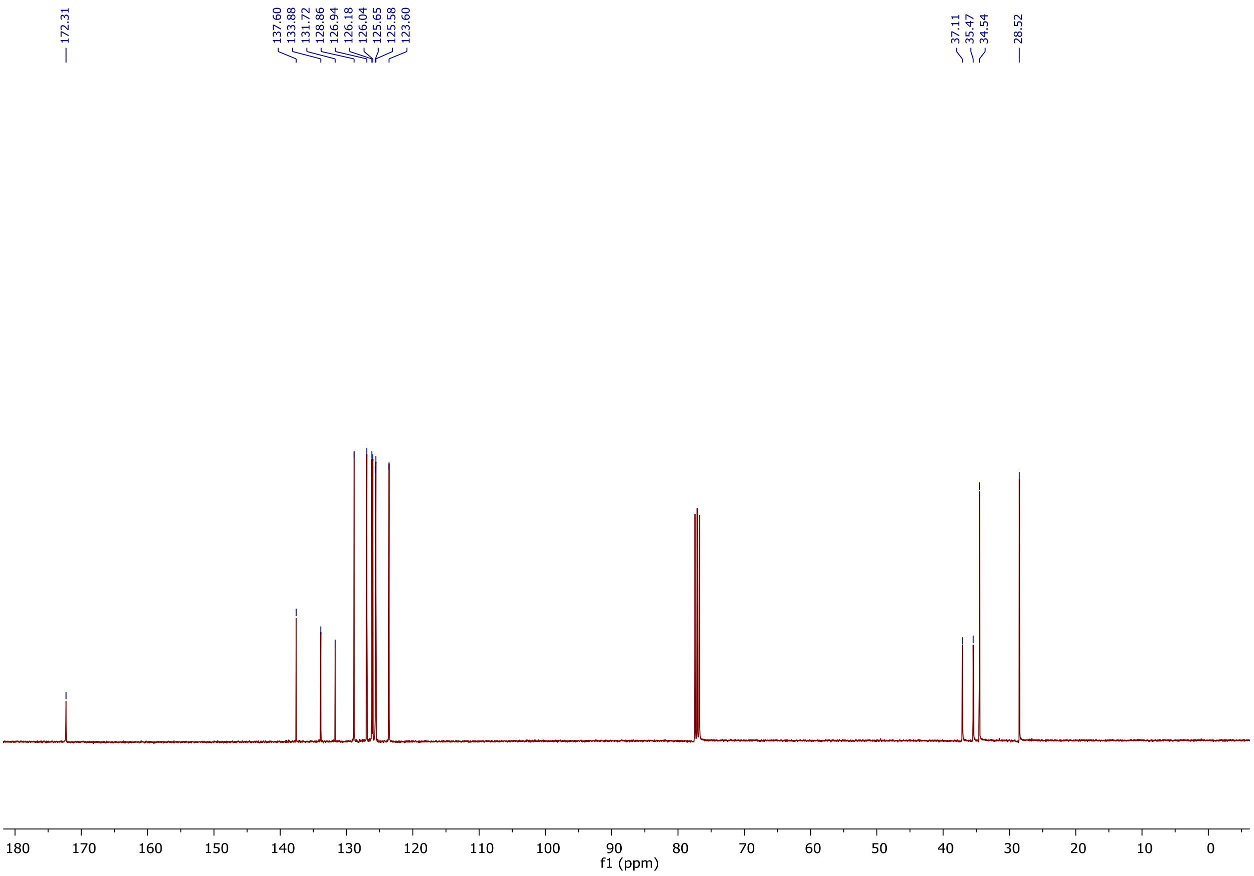

***N*,*N*-Dimethyl 3-(4-chlorophenyl)propanamide (12)**

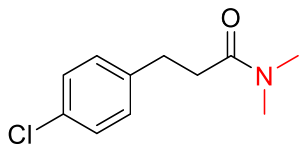

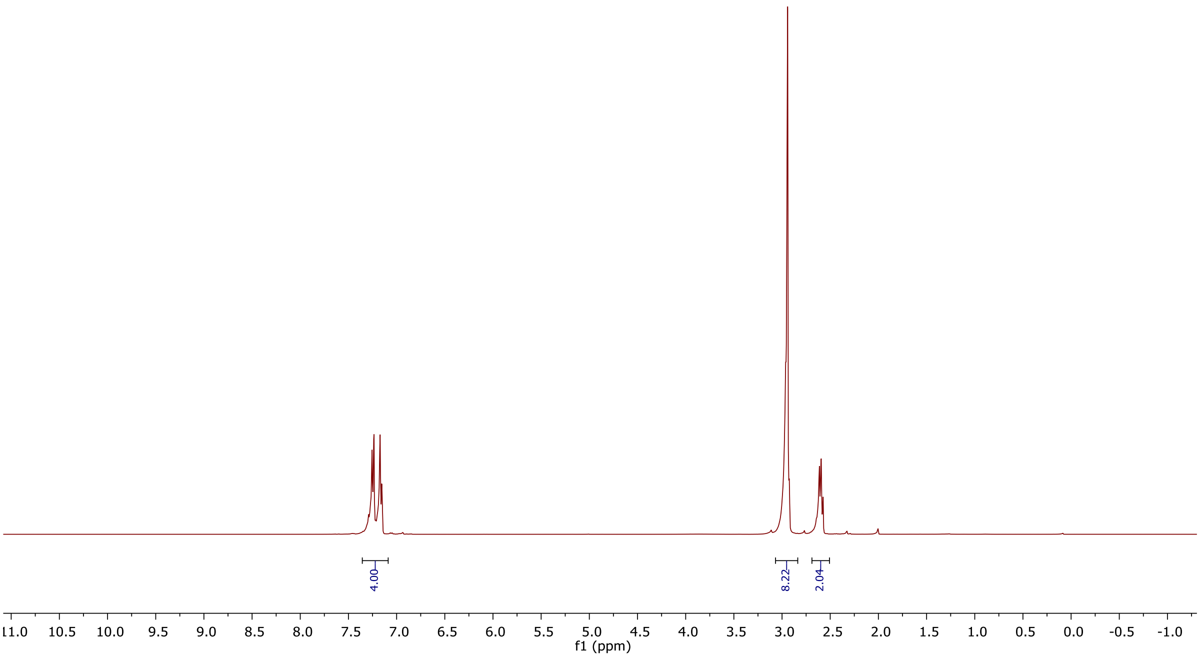

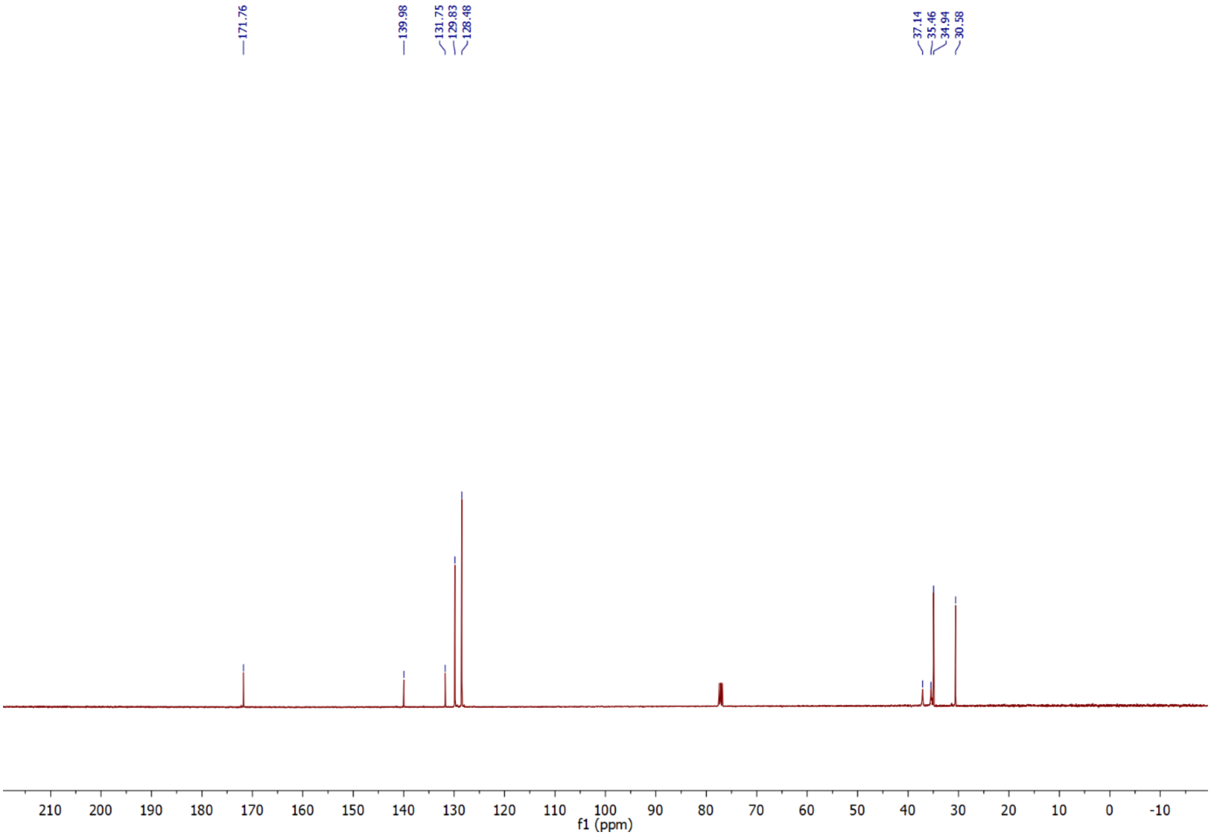

***N*,*N*-Diethyl 3-(4-methylphenyl)propanamide (13)**

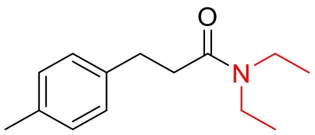

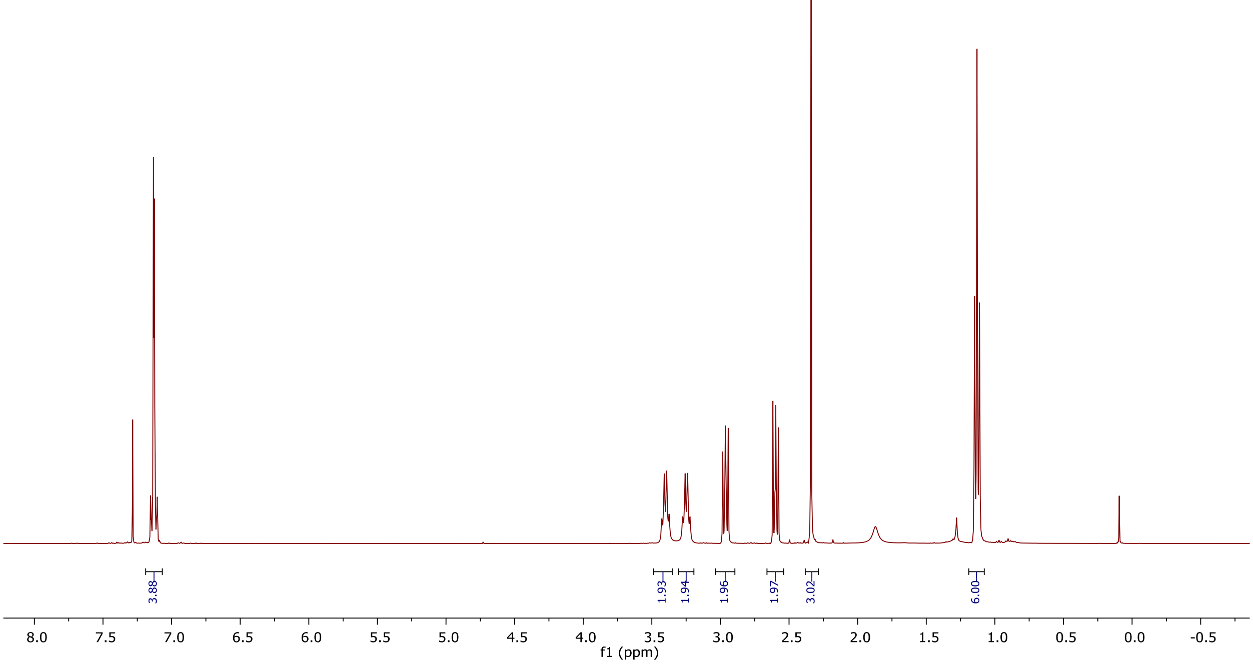

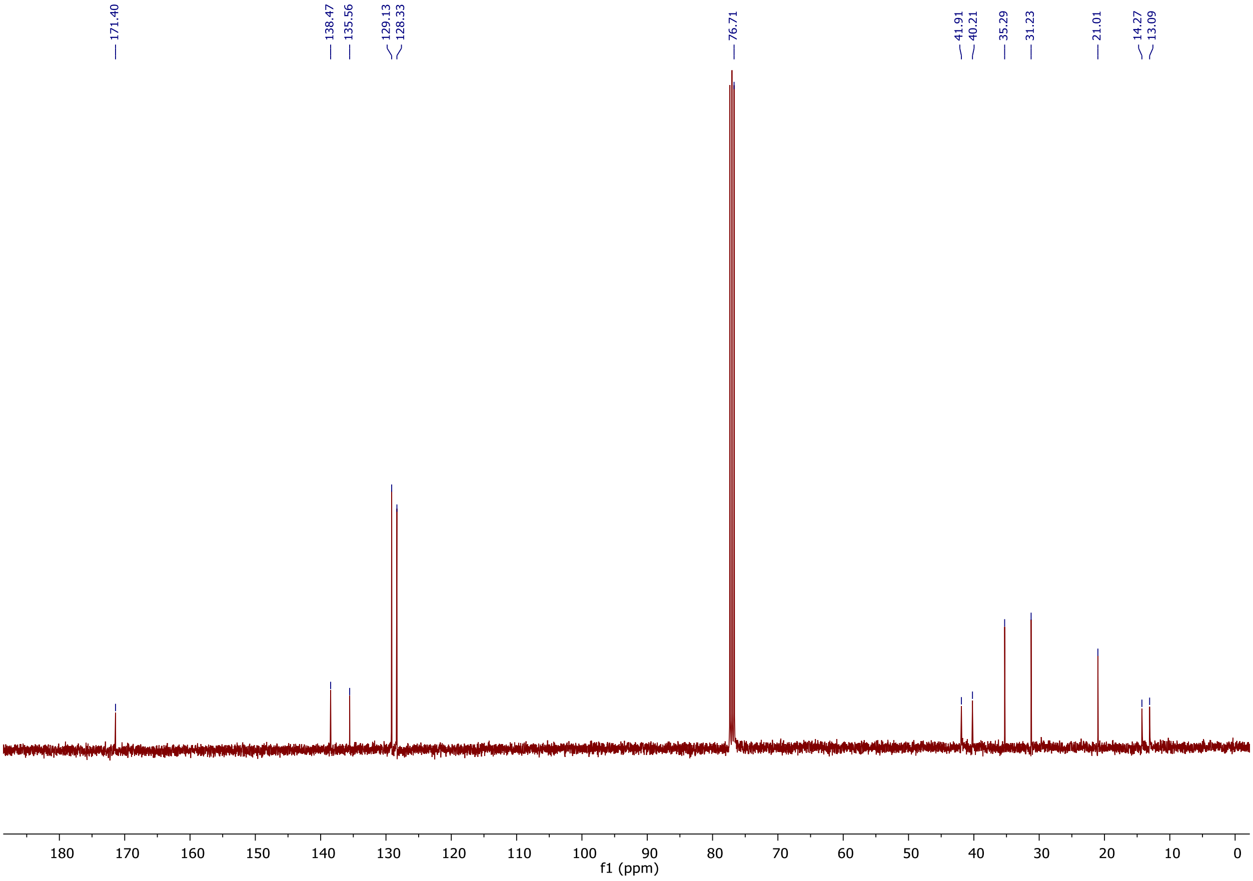

***N,N*-Diethyl 3-(4-methoxyphenyl)propanamide (14)**

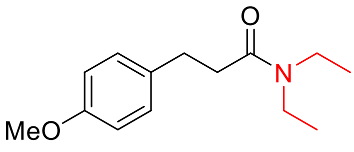

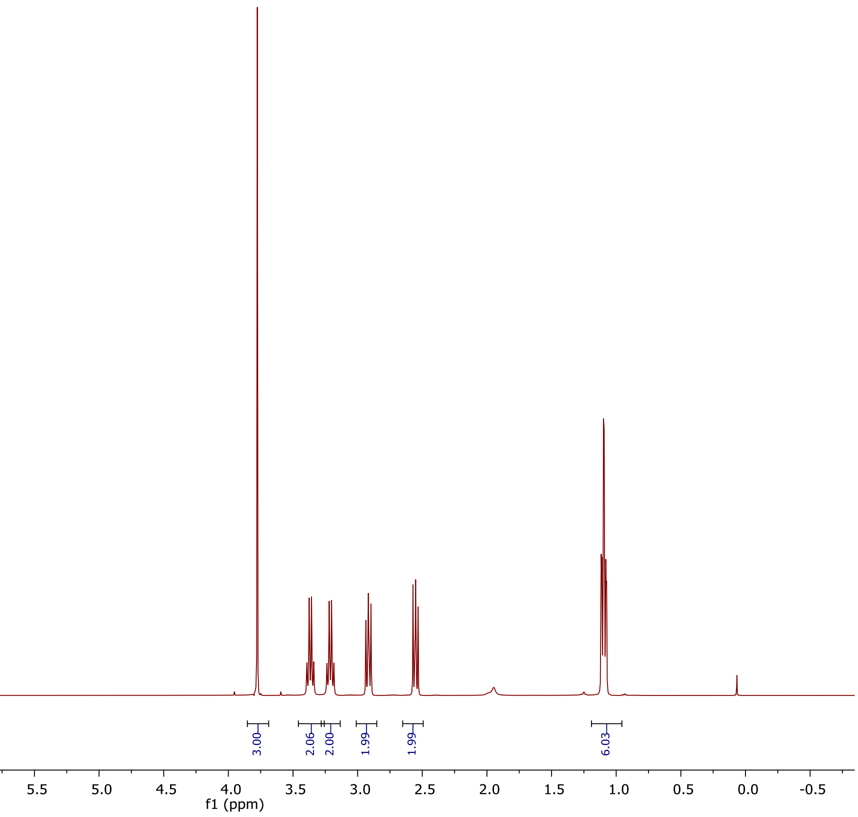

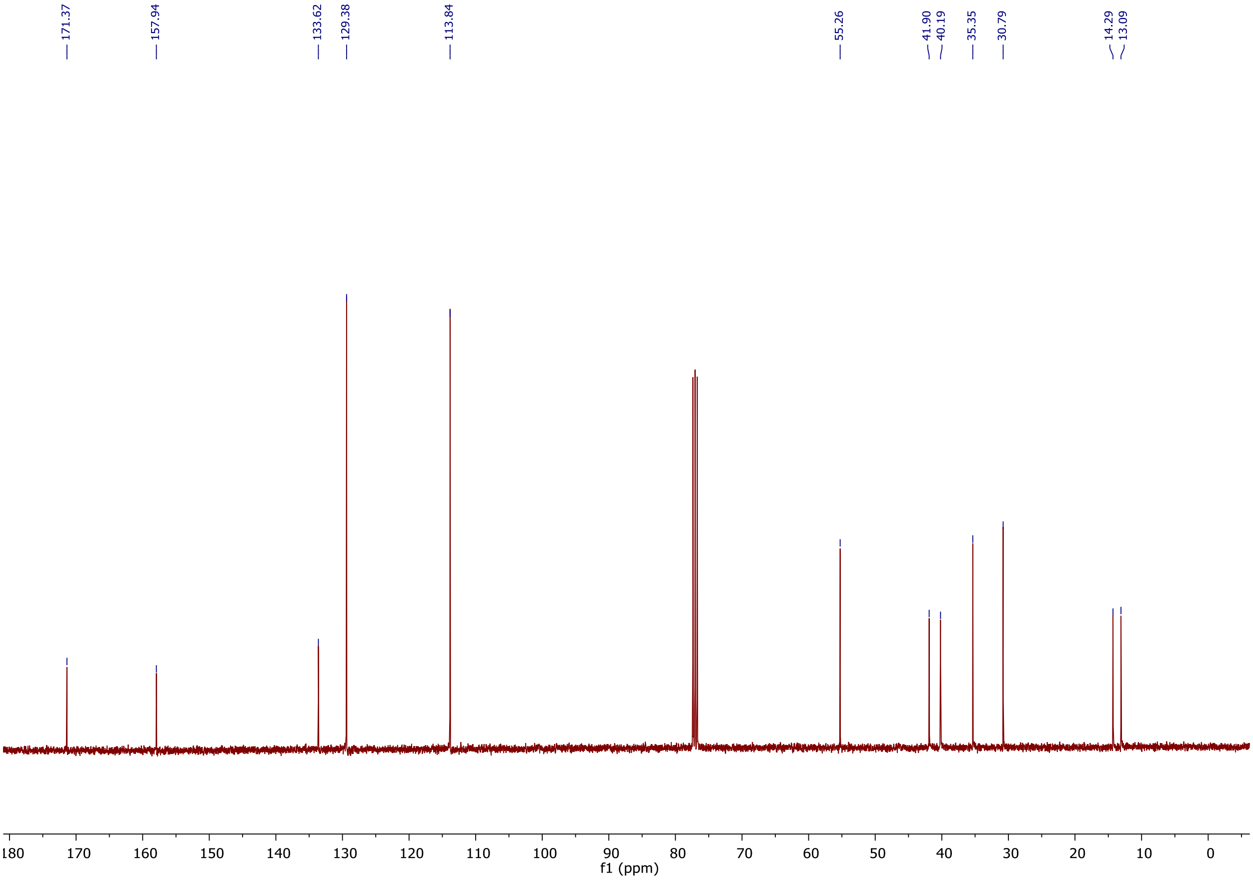

N,*N*-Diethyl 3-(4-(benzyloxy)phenyl)propanamide (15)

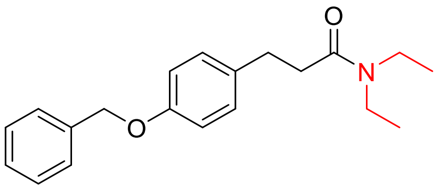

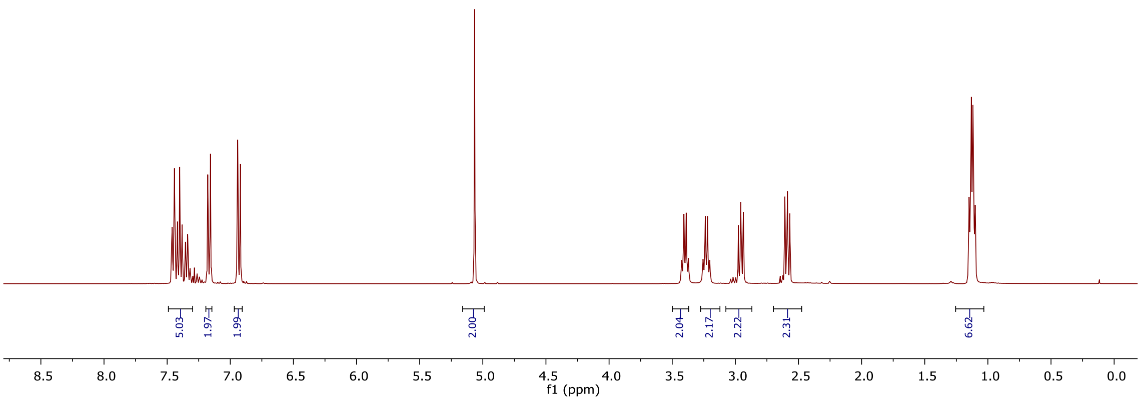

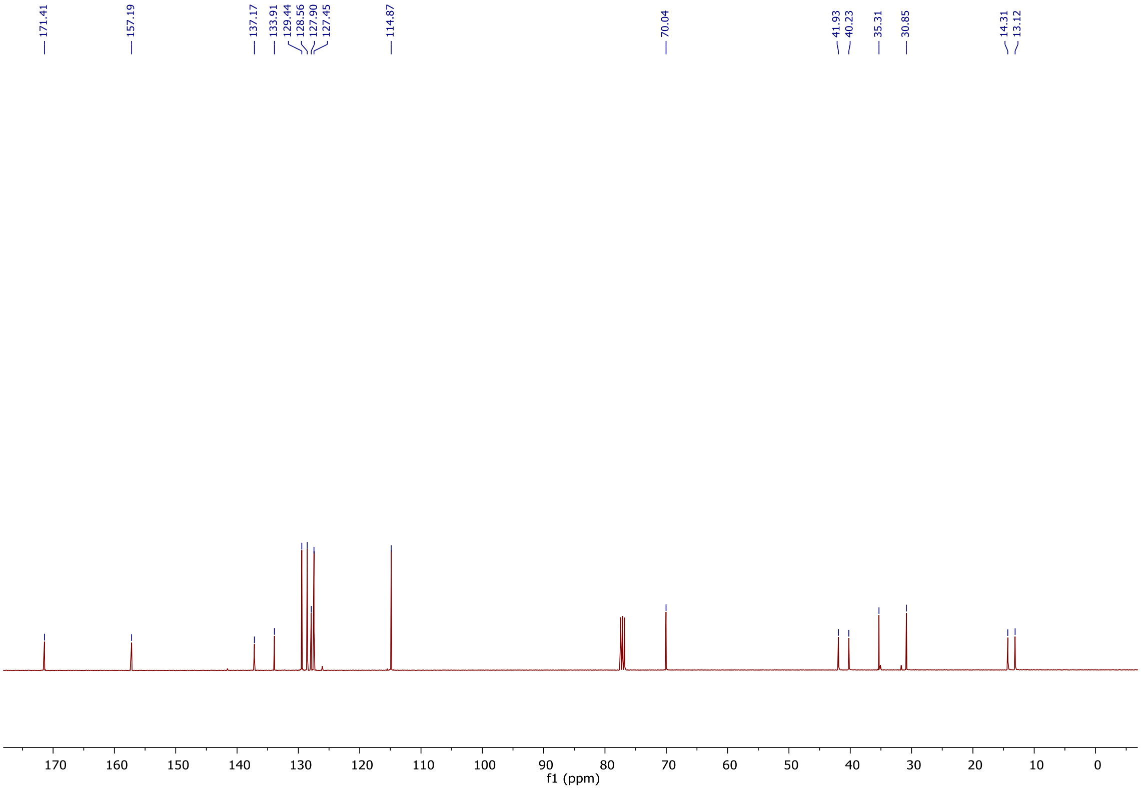

_N,N_-Diethyl 3-(3,4-dimethylphenyl)propanamide (16)

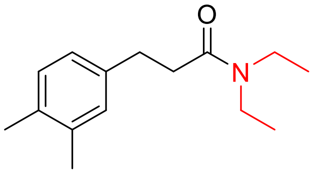

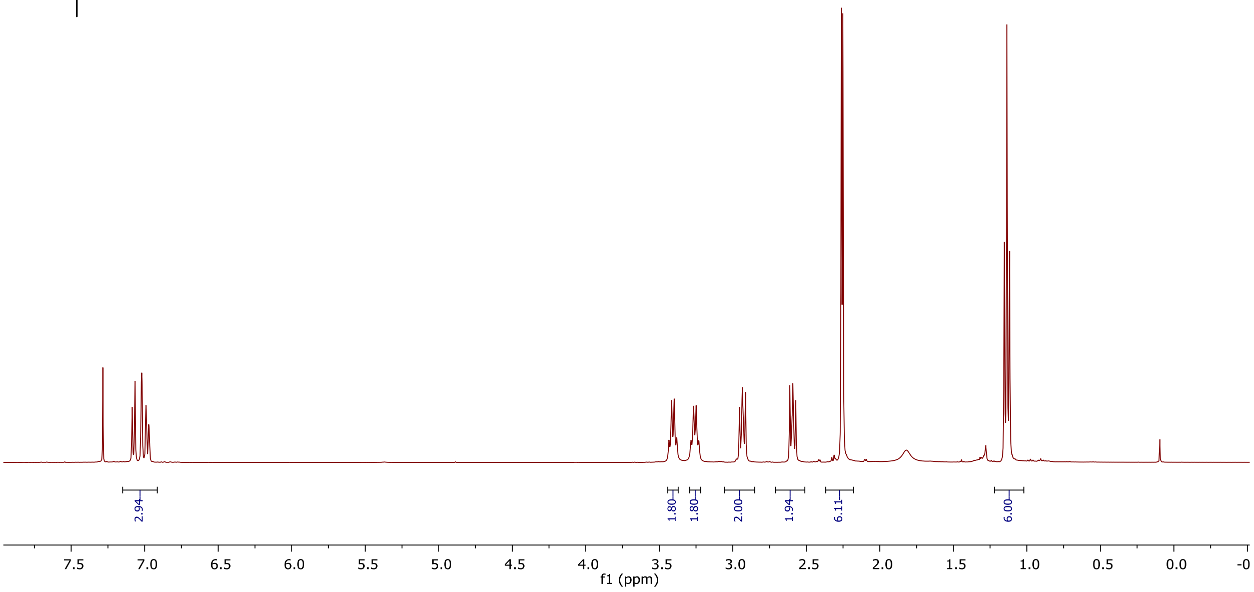

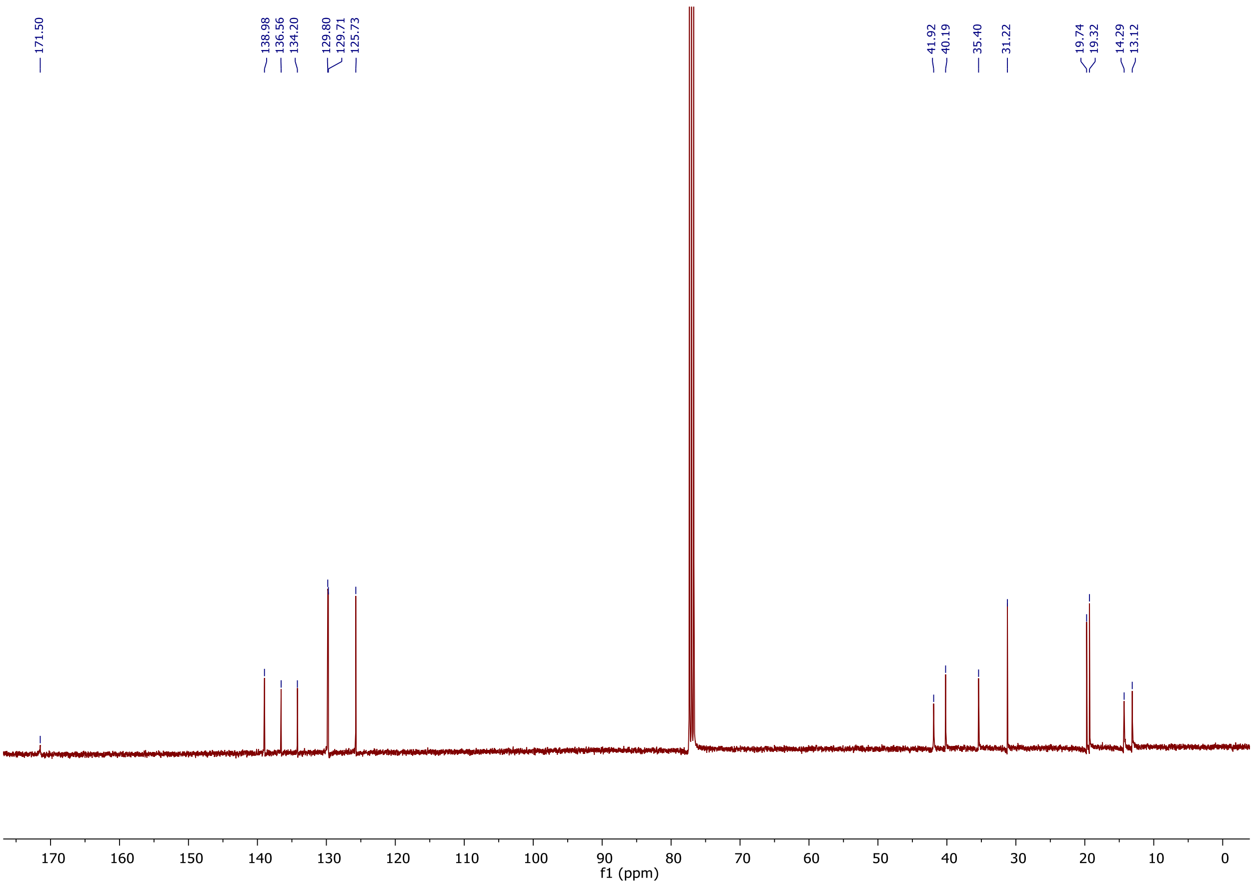

_N,N_-Diethyl 3-(2-methylphenyl)propanamide (17)

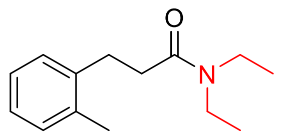

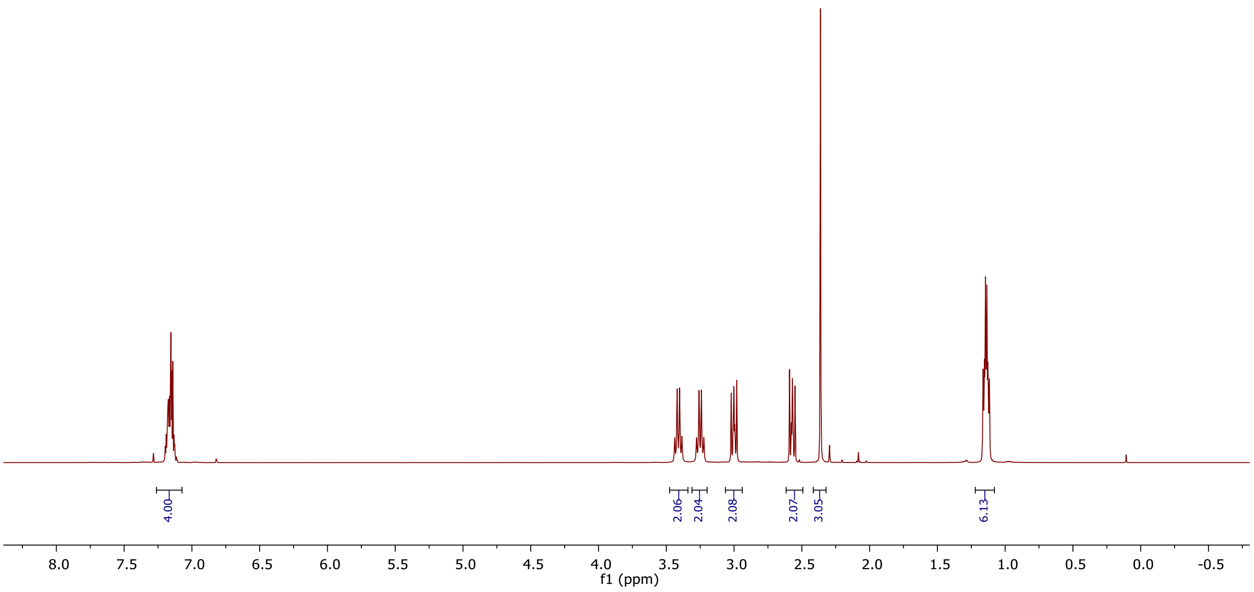

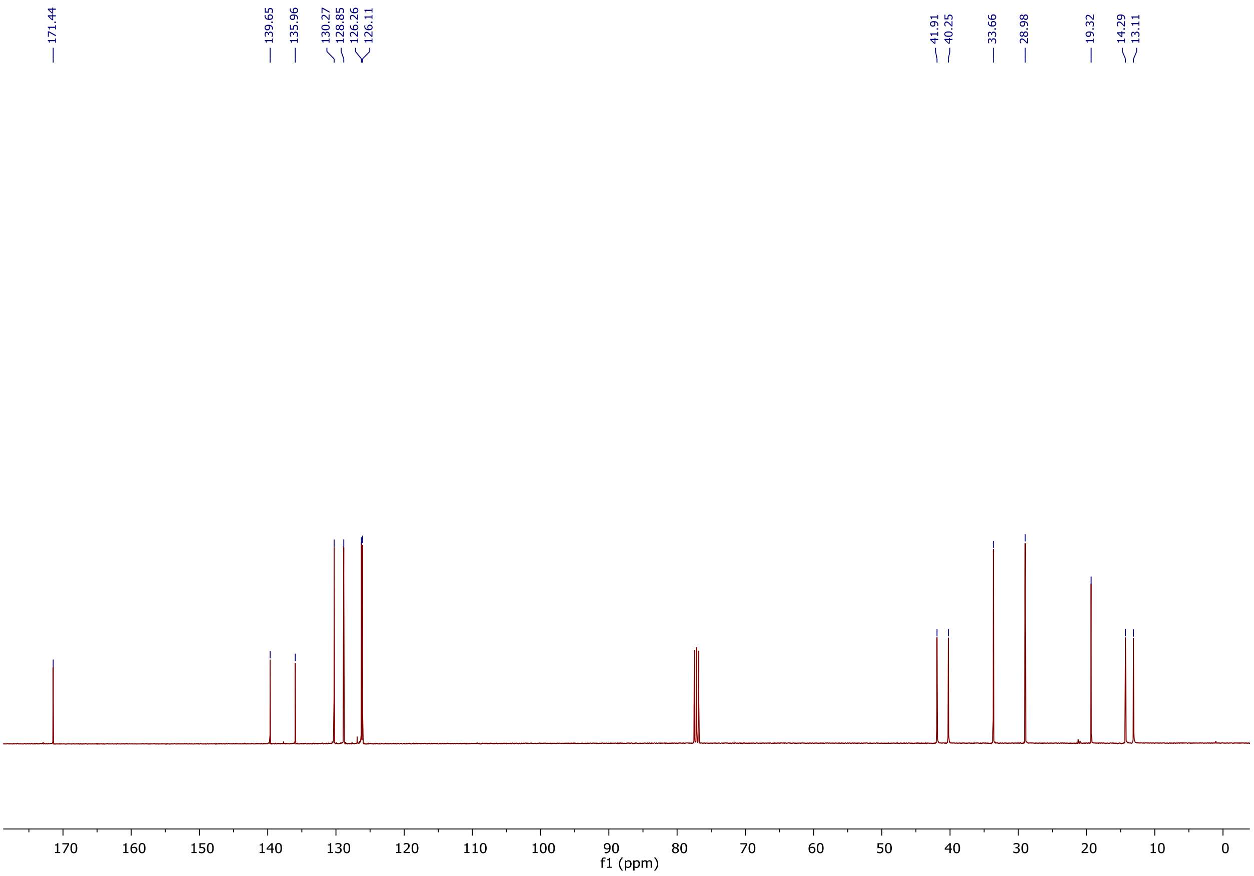

N,*N*-Diethyl 3-(2-methoxyphenyl)propanamide (18)

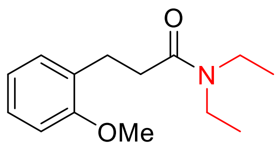

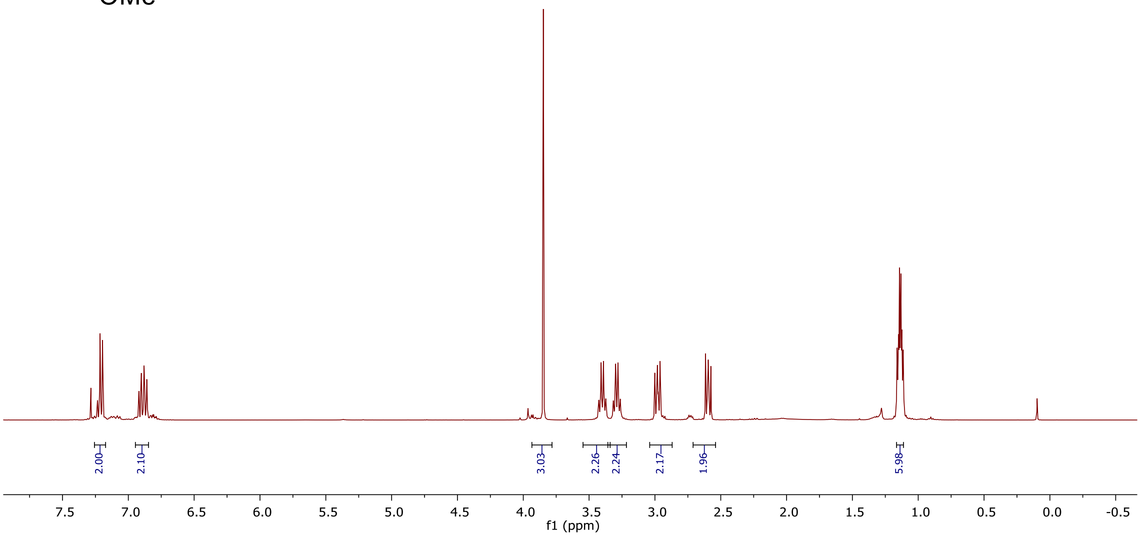

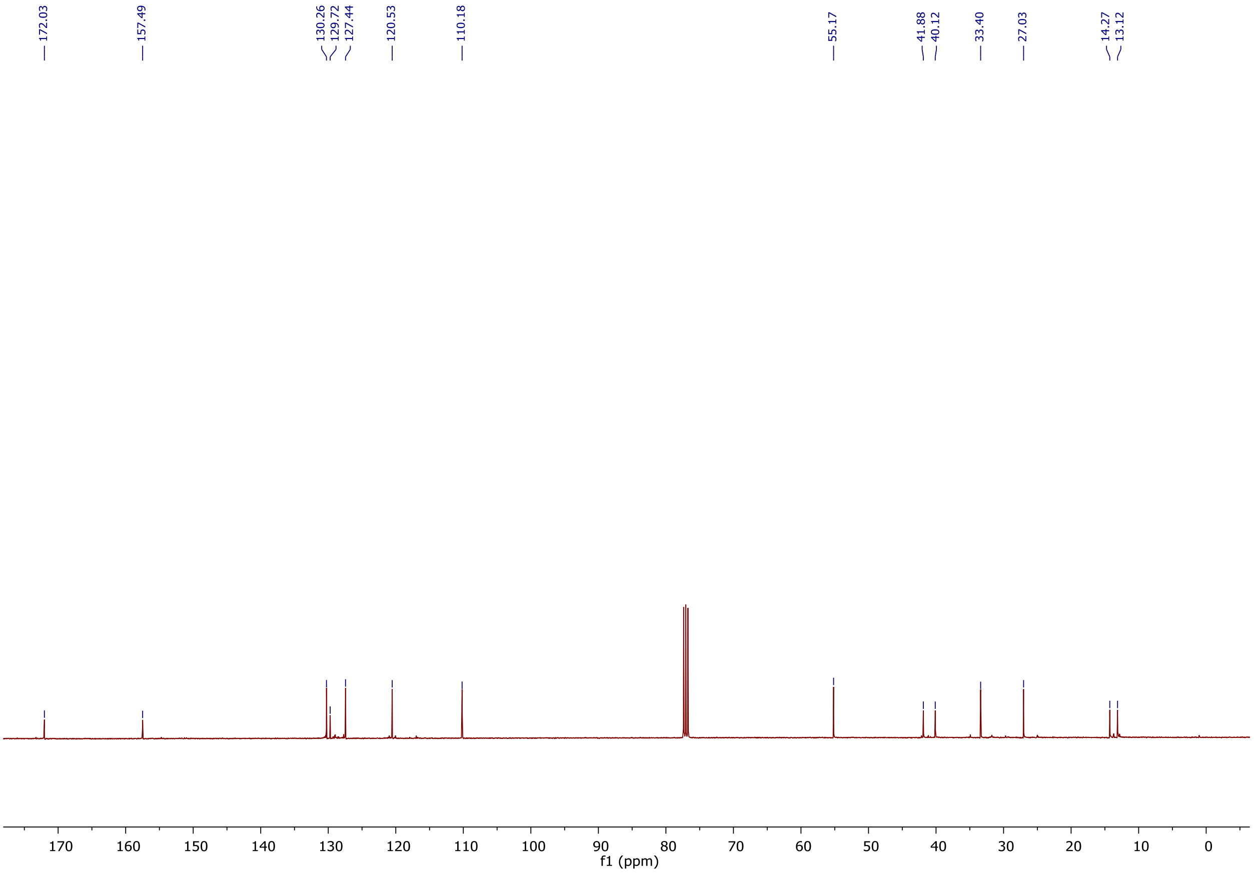

***N*,*N*-Diethyl 3-([1,1'-biphenyl]-4-yl)propanamide (19)**

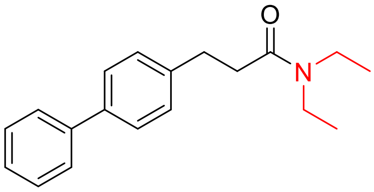

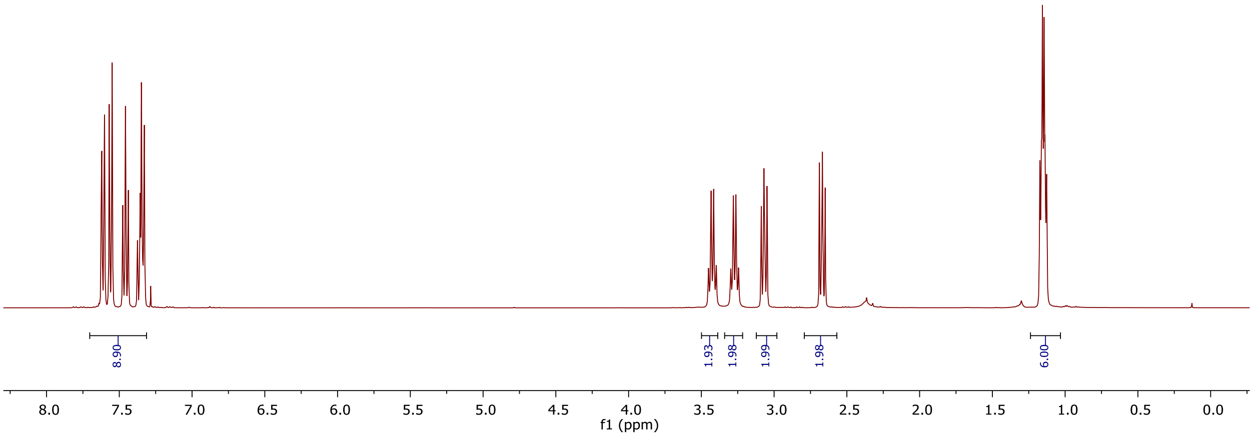

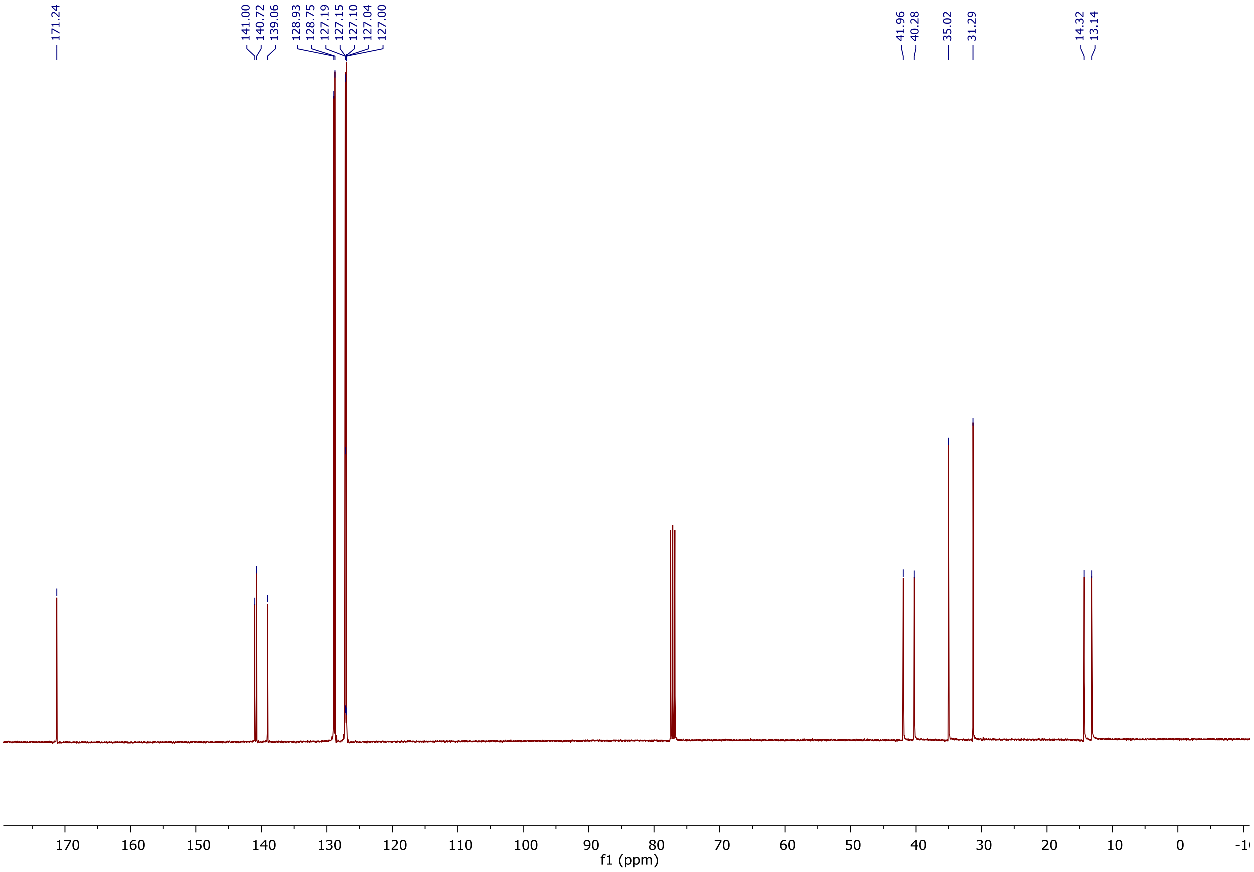

***N*,*N*-Diethyl 3-(4-chlorophenyl)propanamide (20)**

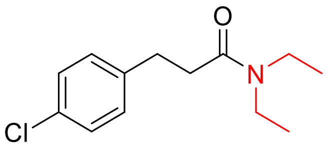

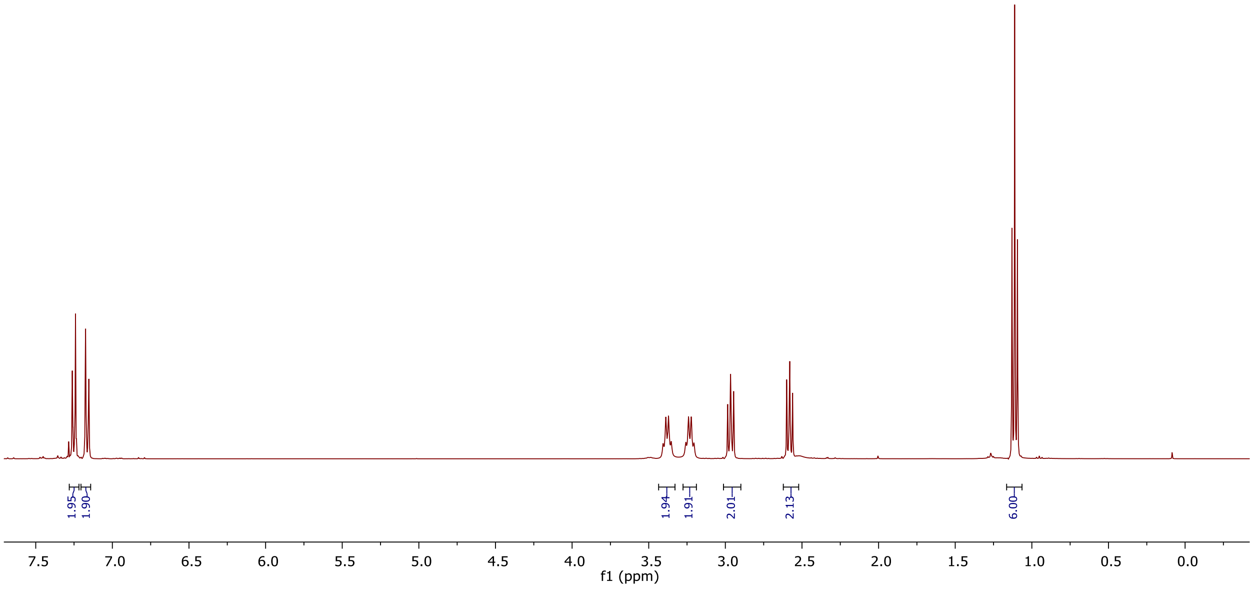

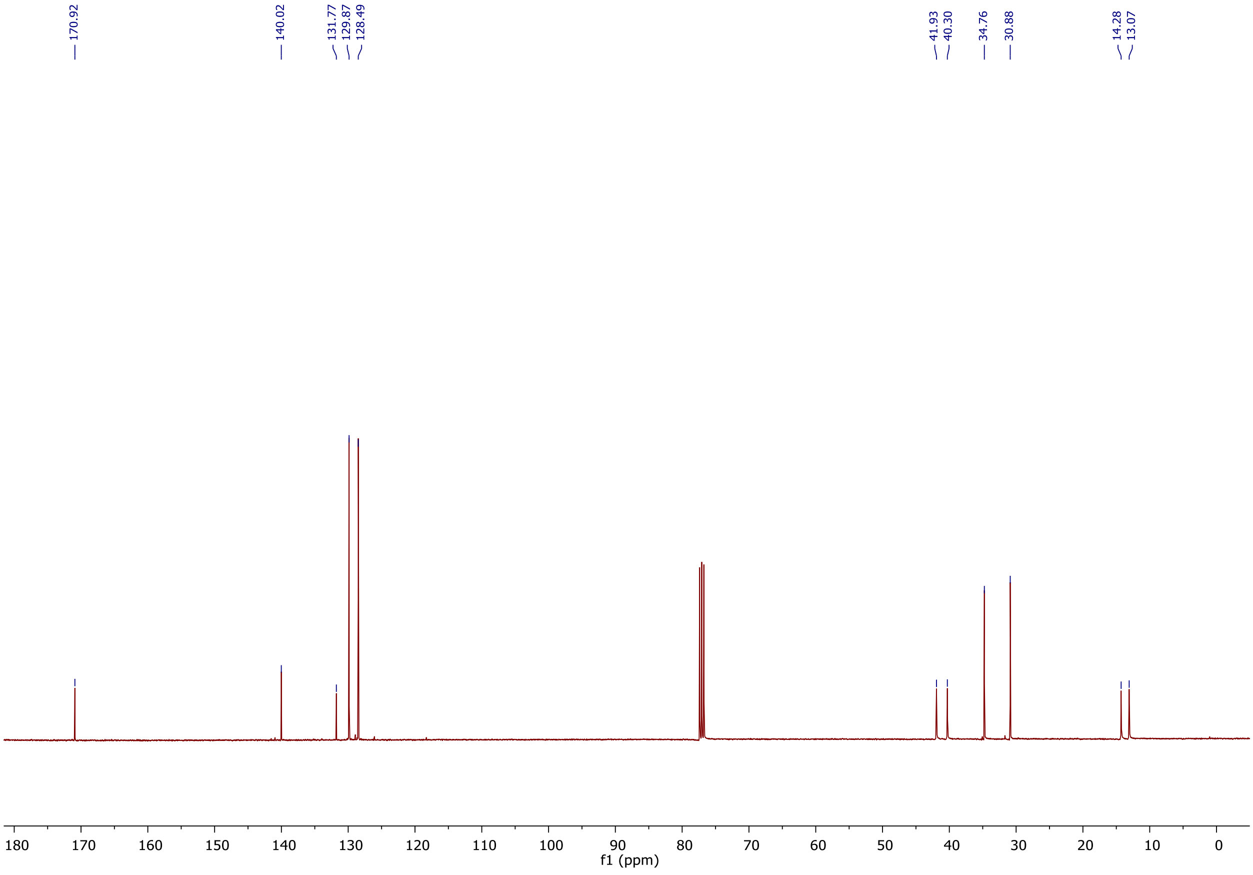

3-Phenyl-1-(piperidin-1-yl)propan-1-one (21)

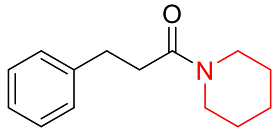

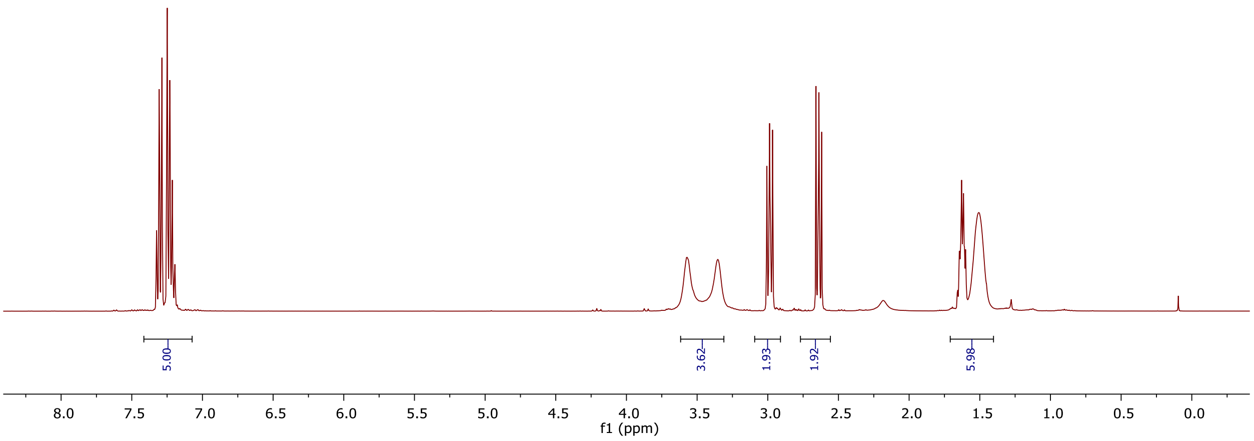

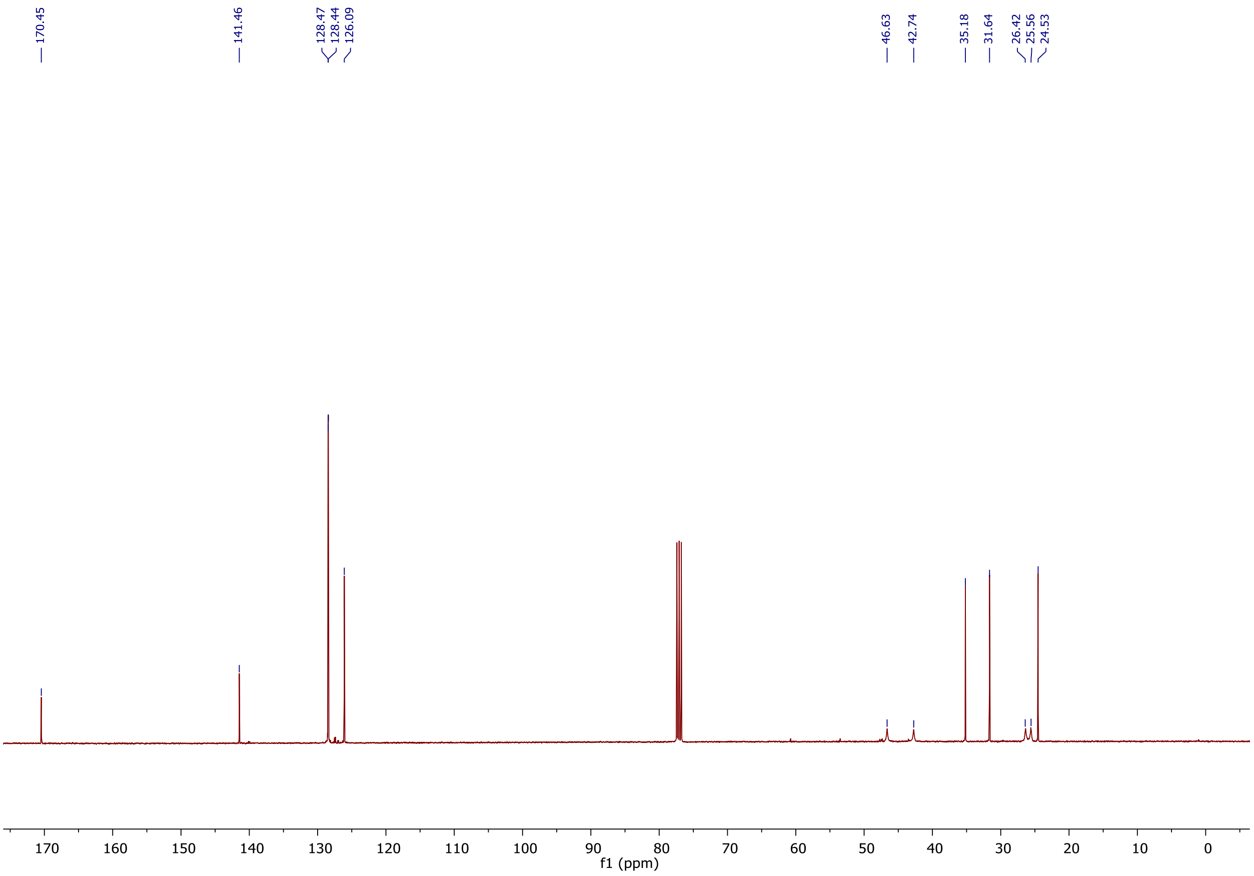

1-Morpholino-3-phenylpropan-1-one (22)

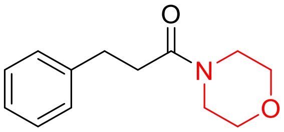

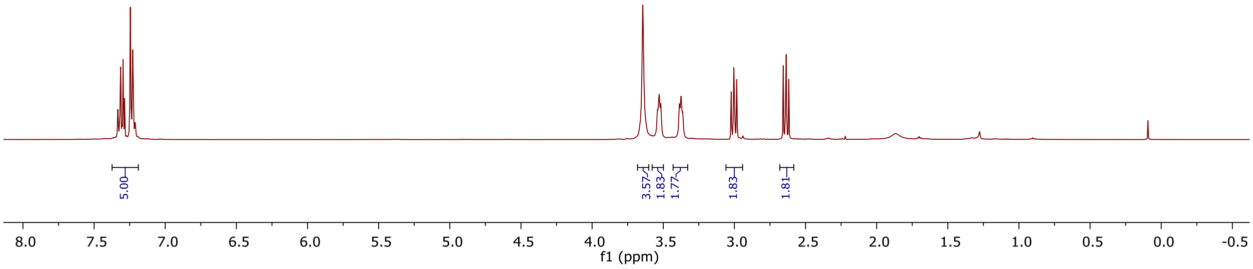

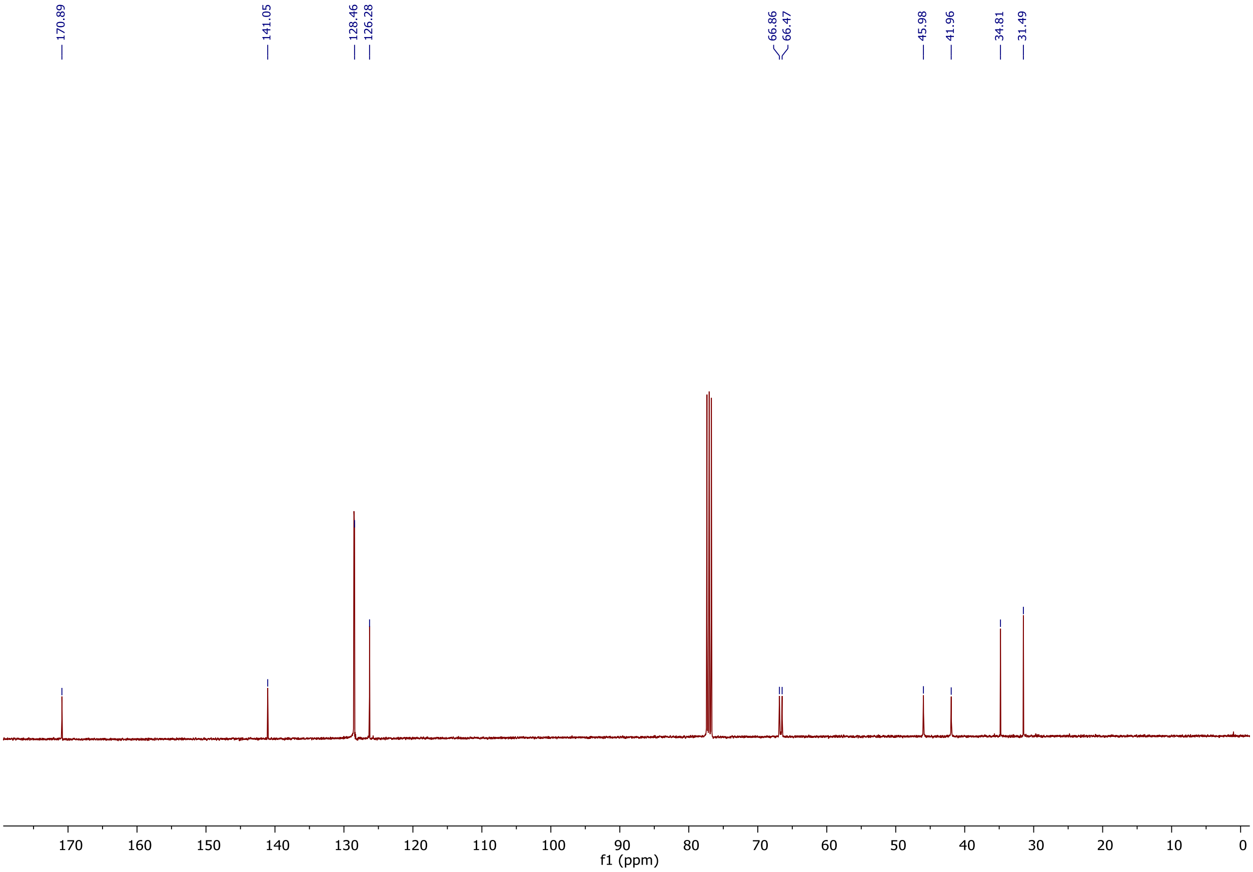

3-(Naphthalen-1-yl)-1-(piperidin-1-yl)propan-1-one (23)

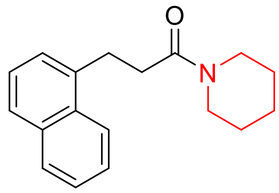

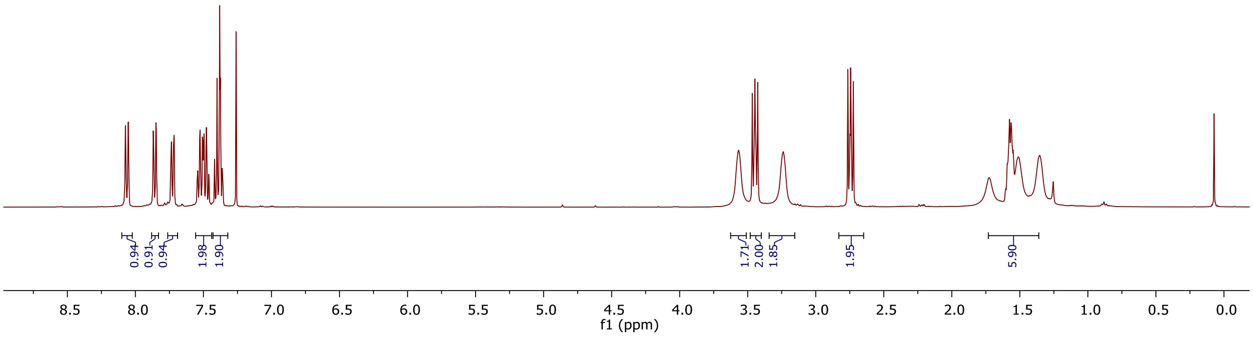

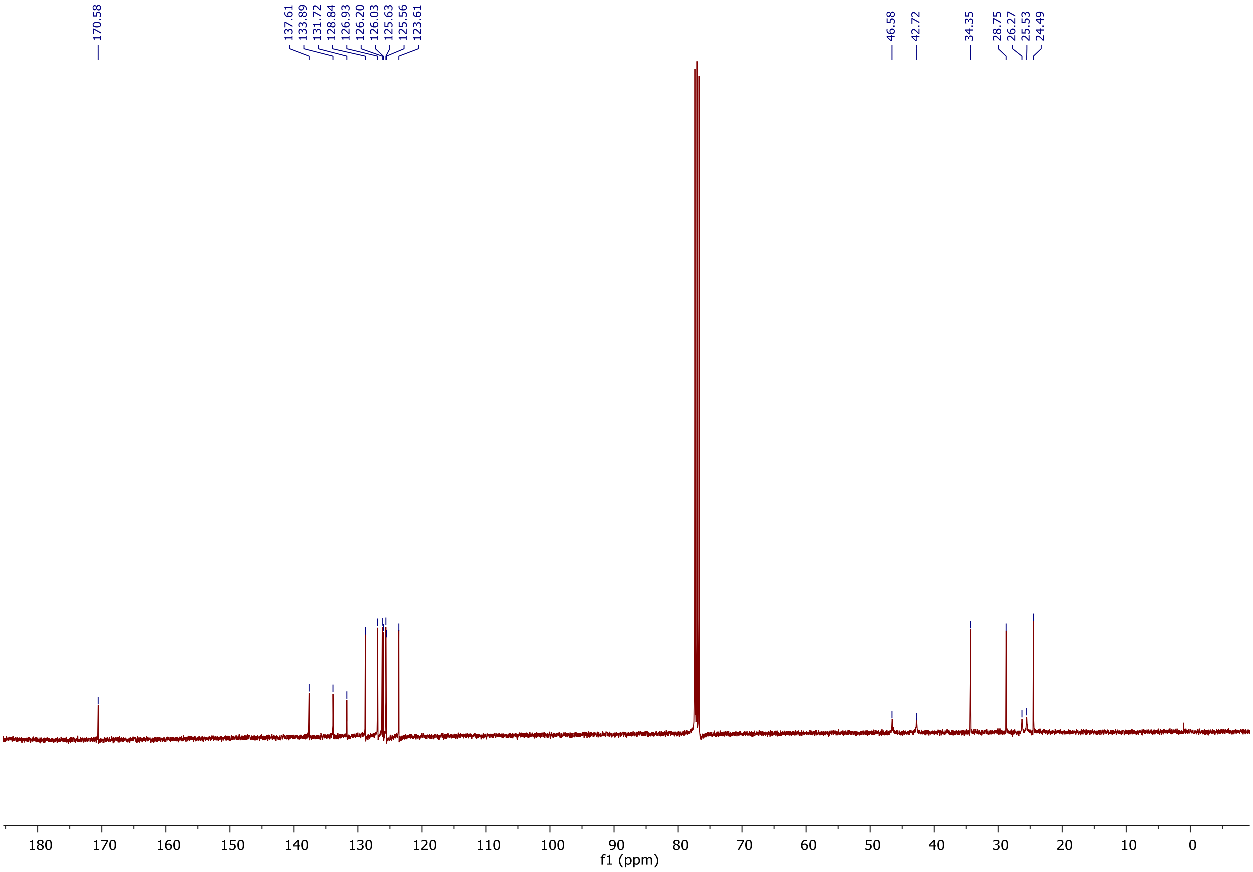

1-Morpholino-3-(naphthalen-1-yl)propan-1-one (24)

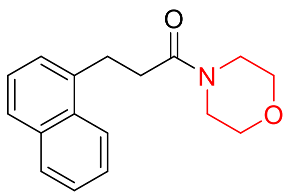

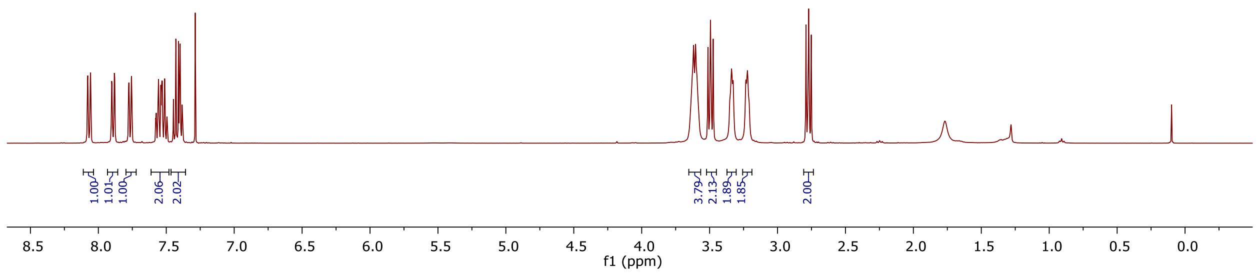

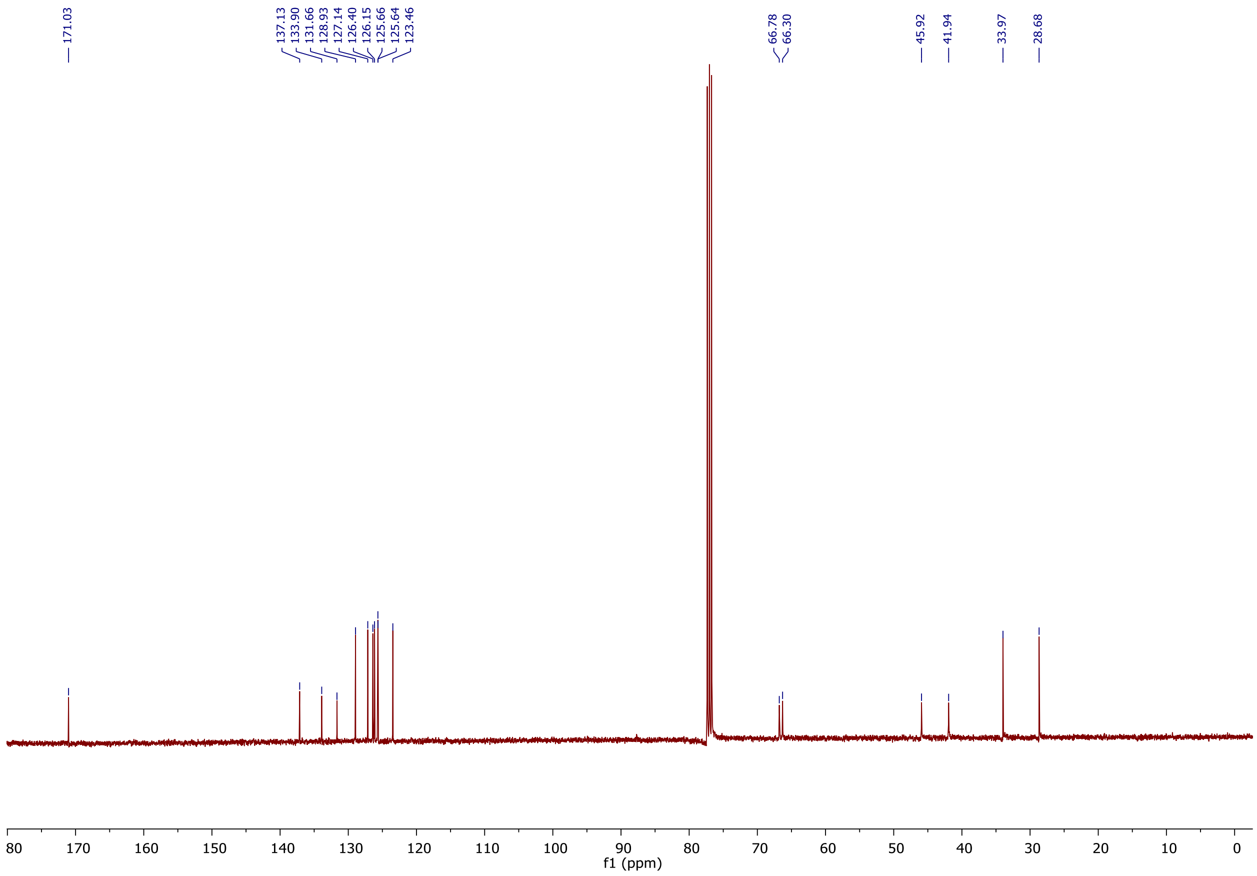

3-(4-Methoxyphenyl)-1-(piperidin-1-yl)propan-1-one (25)

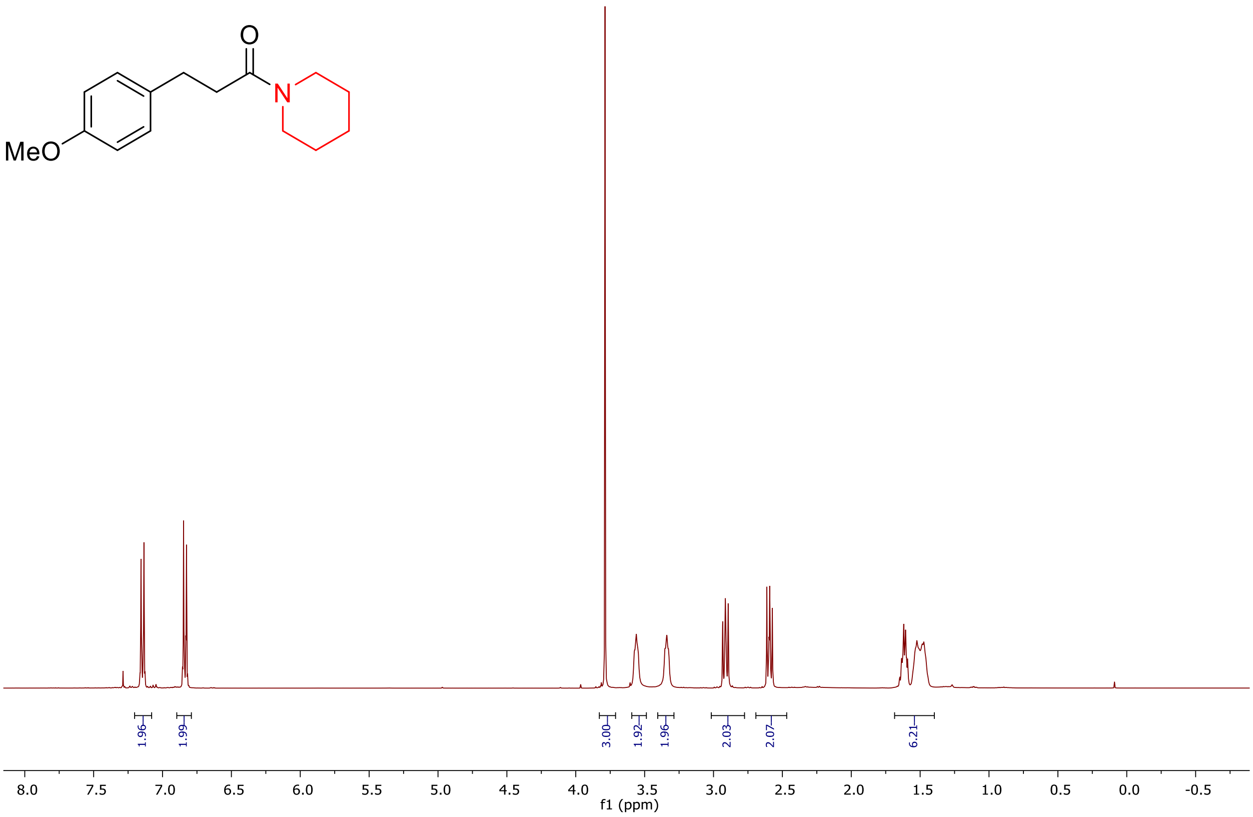

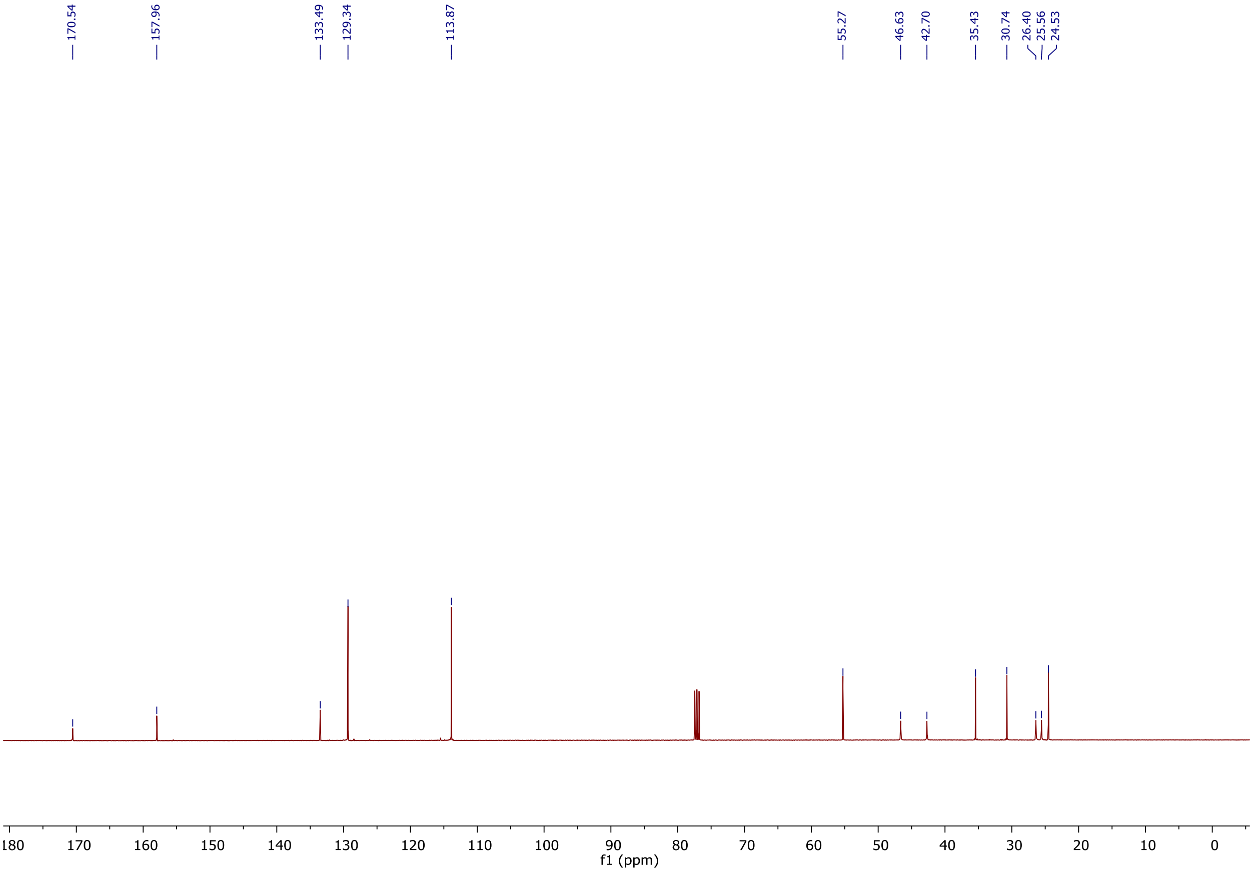

3-(4-Methylphenyl)-1-morpholinopropan-1-one (26)

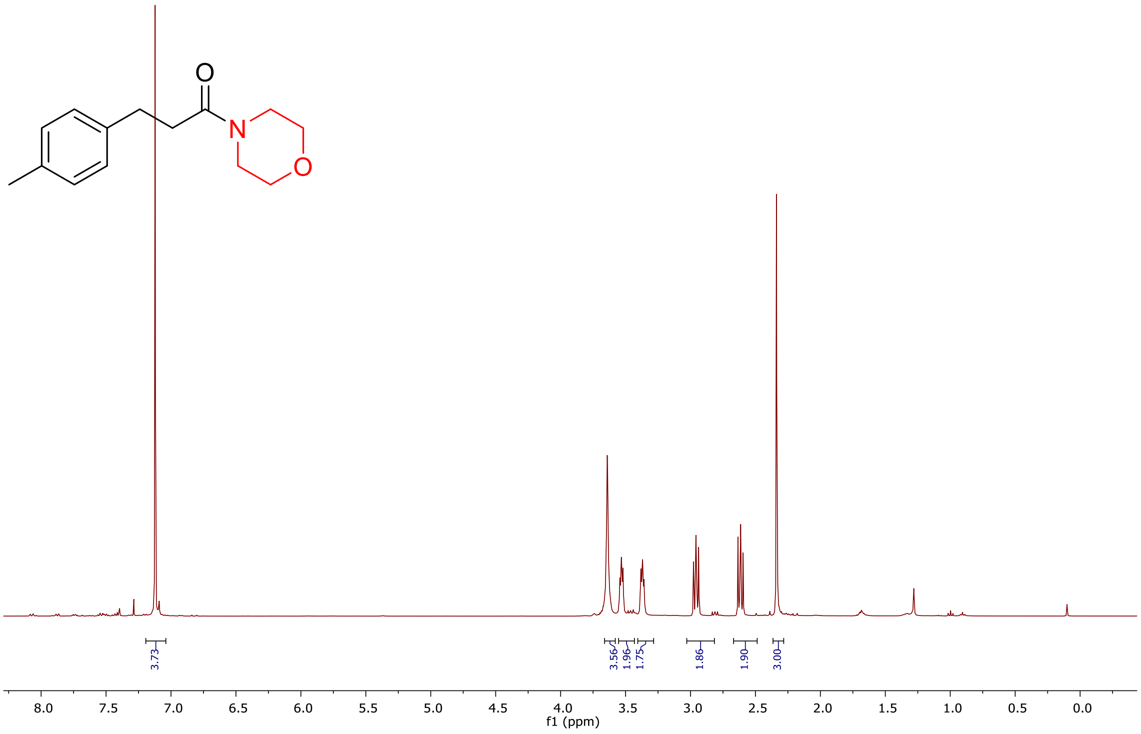

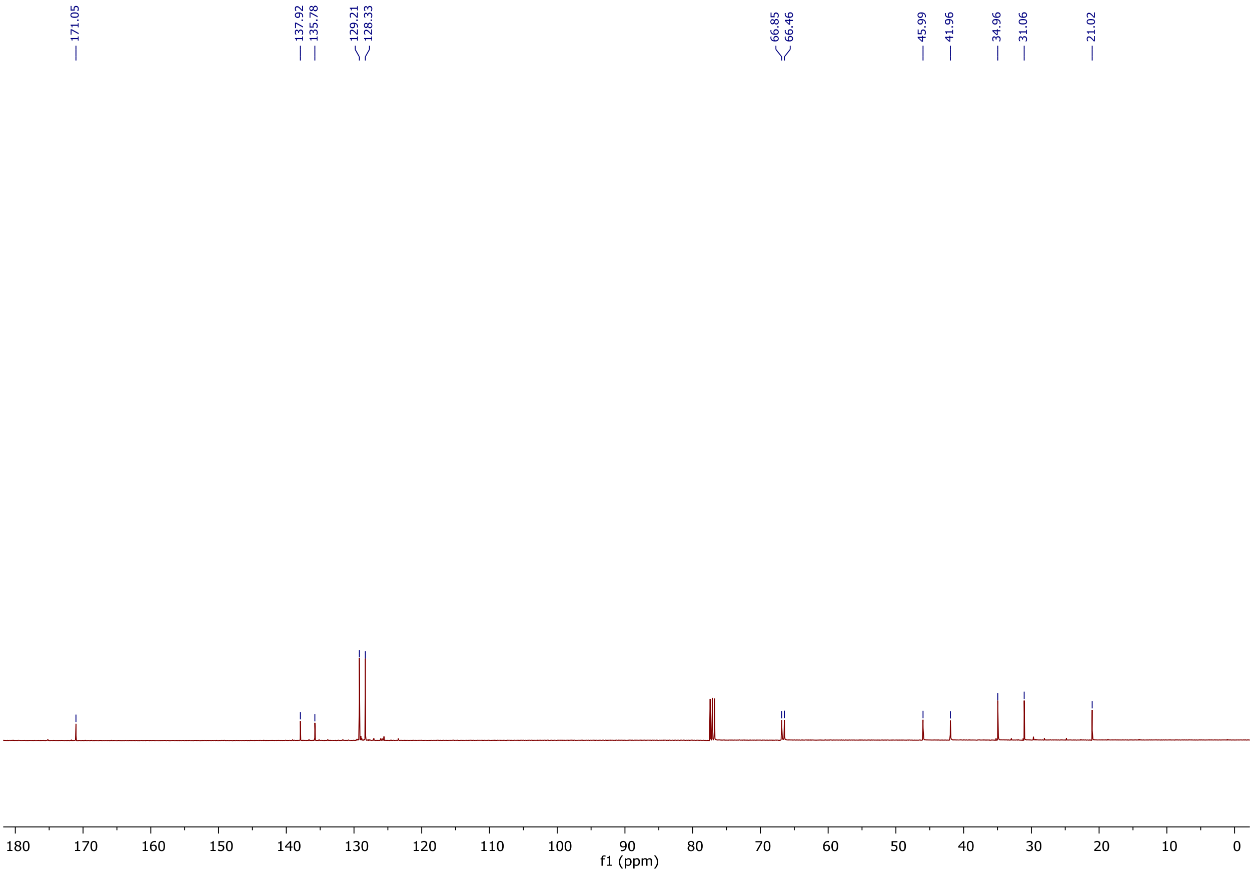

N-Methyl 3-phenylpropanamide (27)

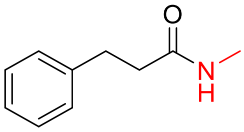

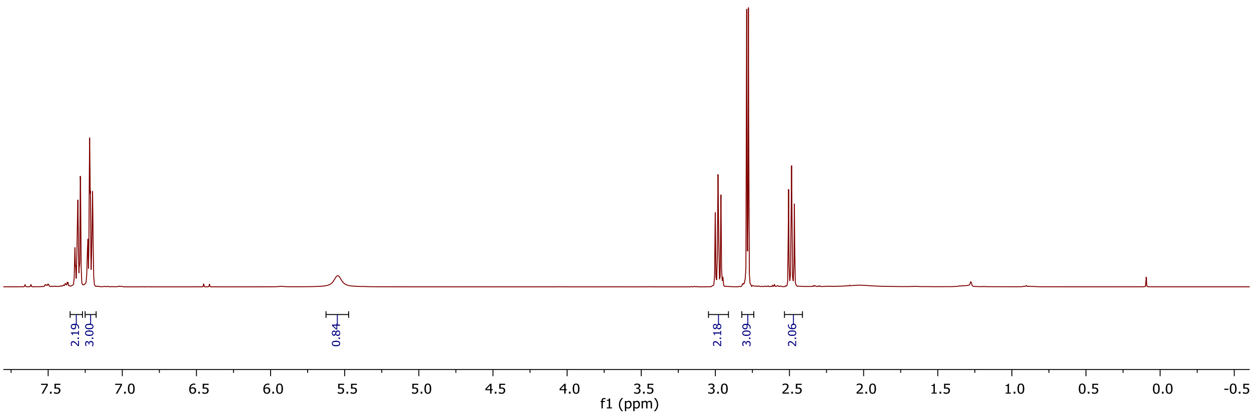

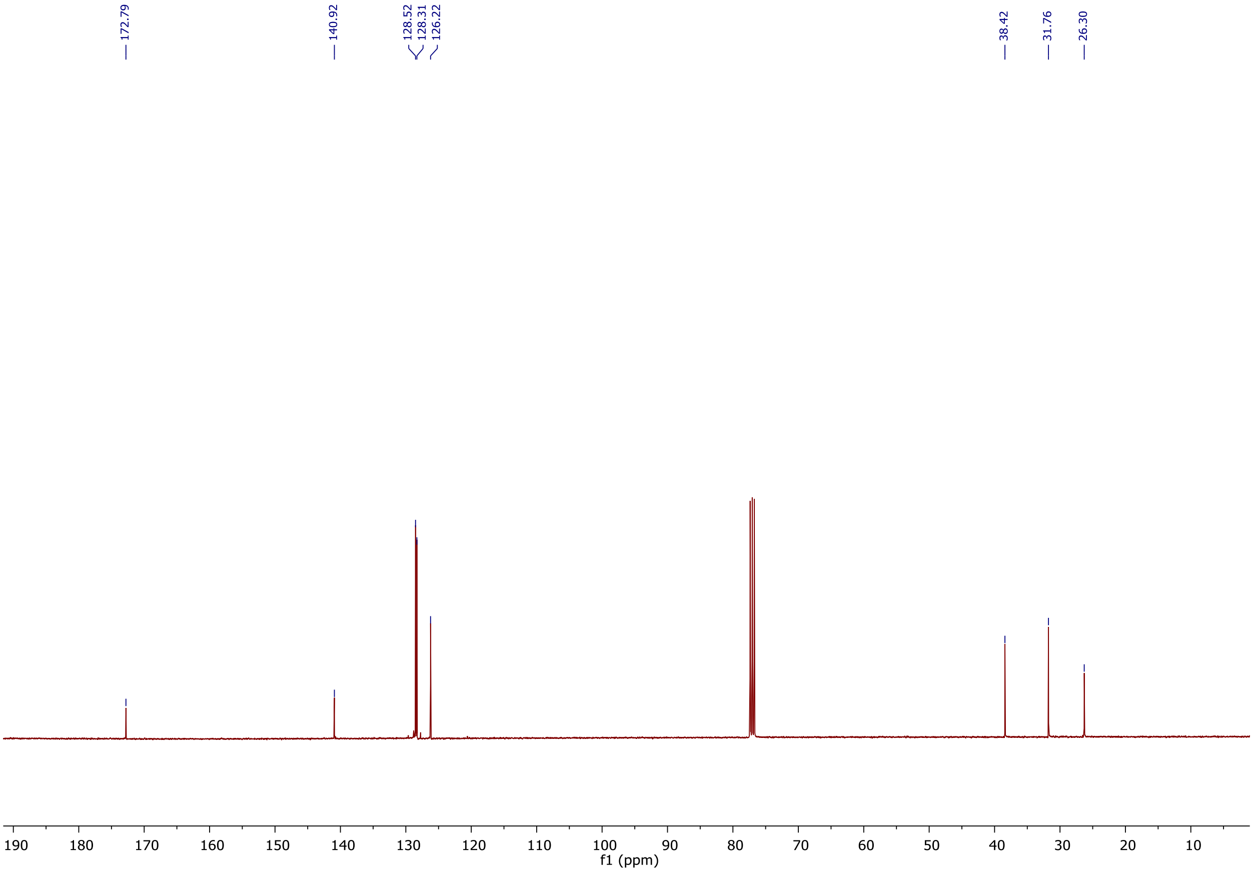

***N*-Methyl 3-(4-(benzyloxy)phenyl)propanamide (28)**

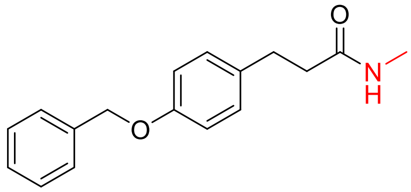

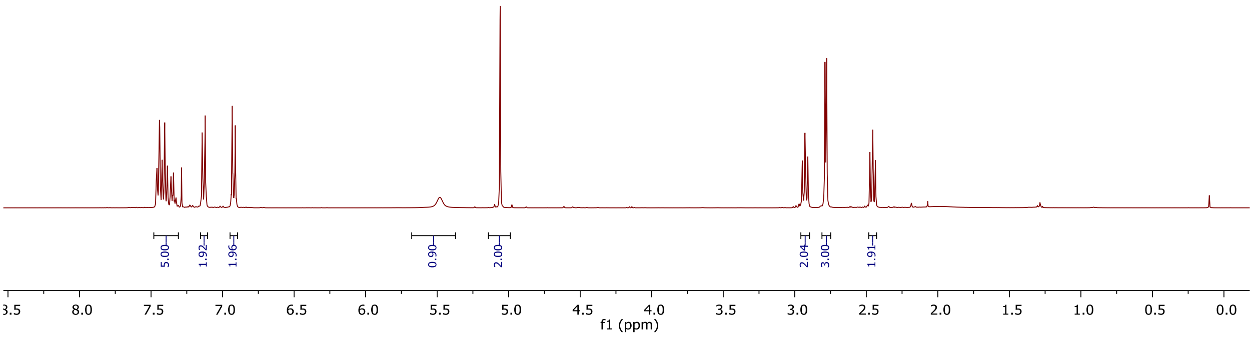

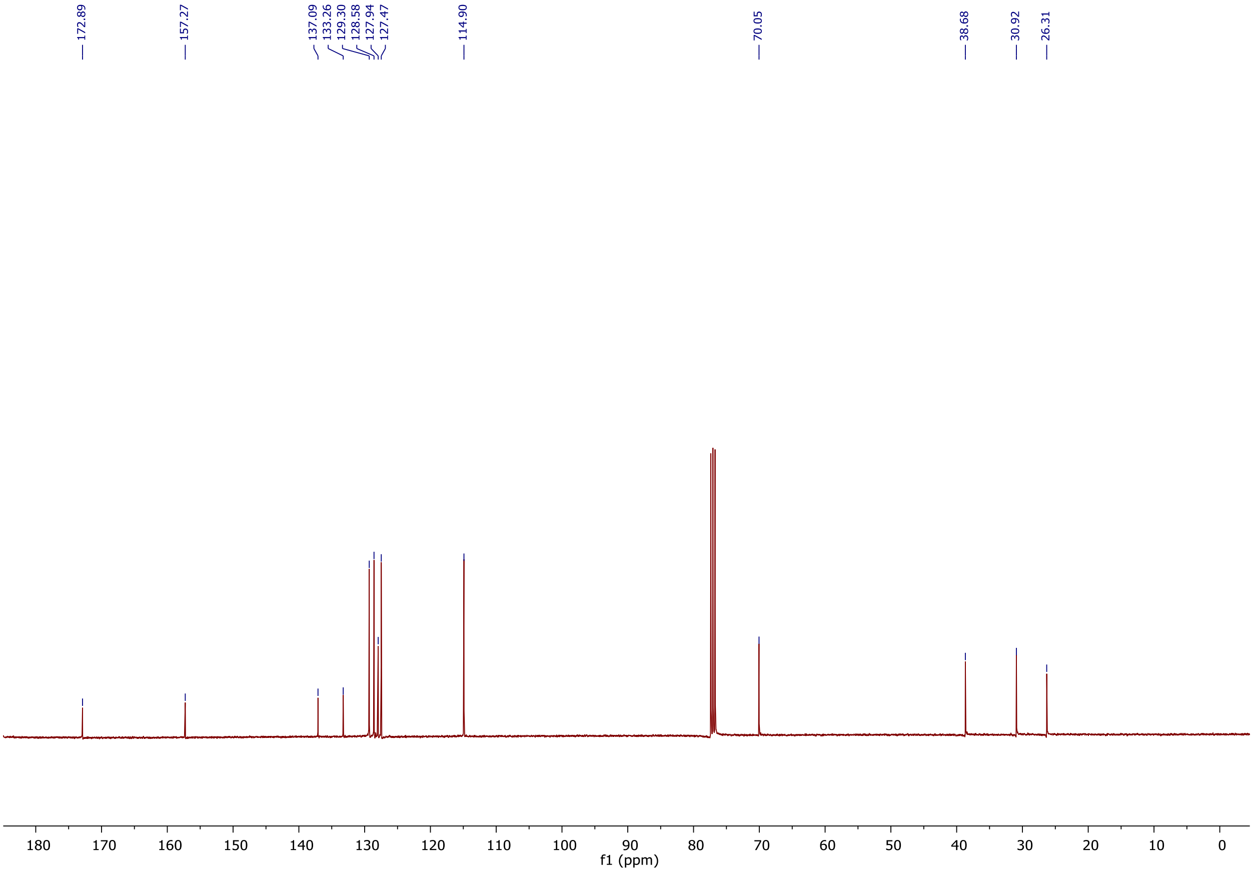

N-Methyl 3-(naphthalen-1-yl)propanamide (29)

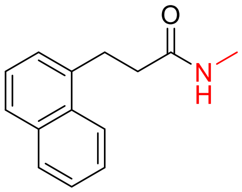

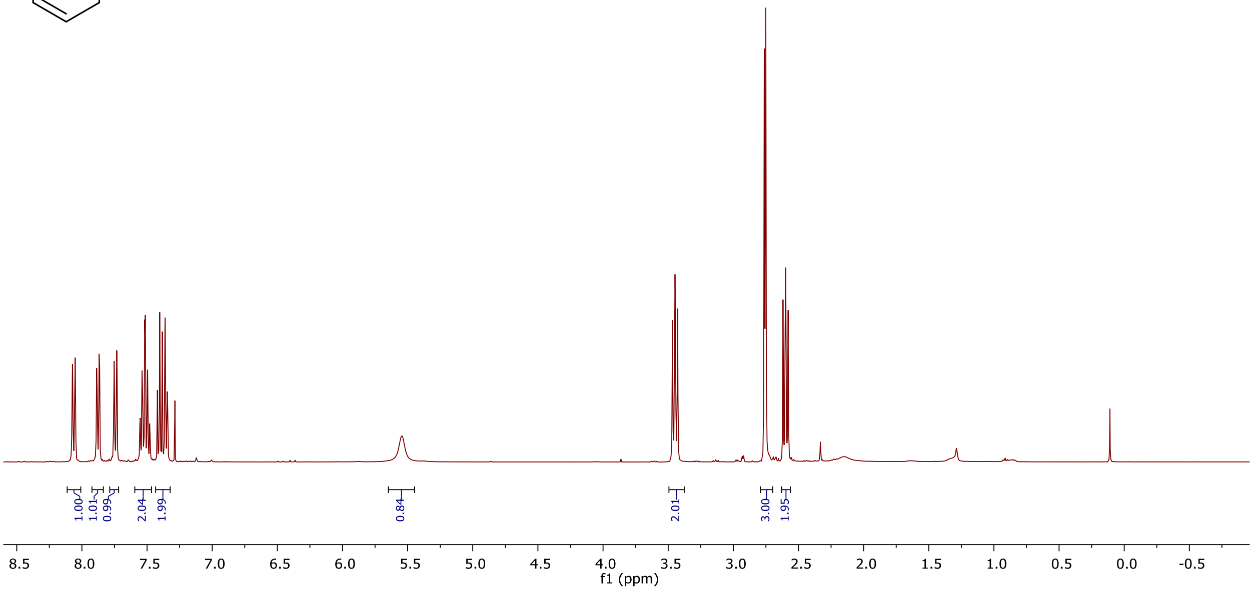

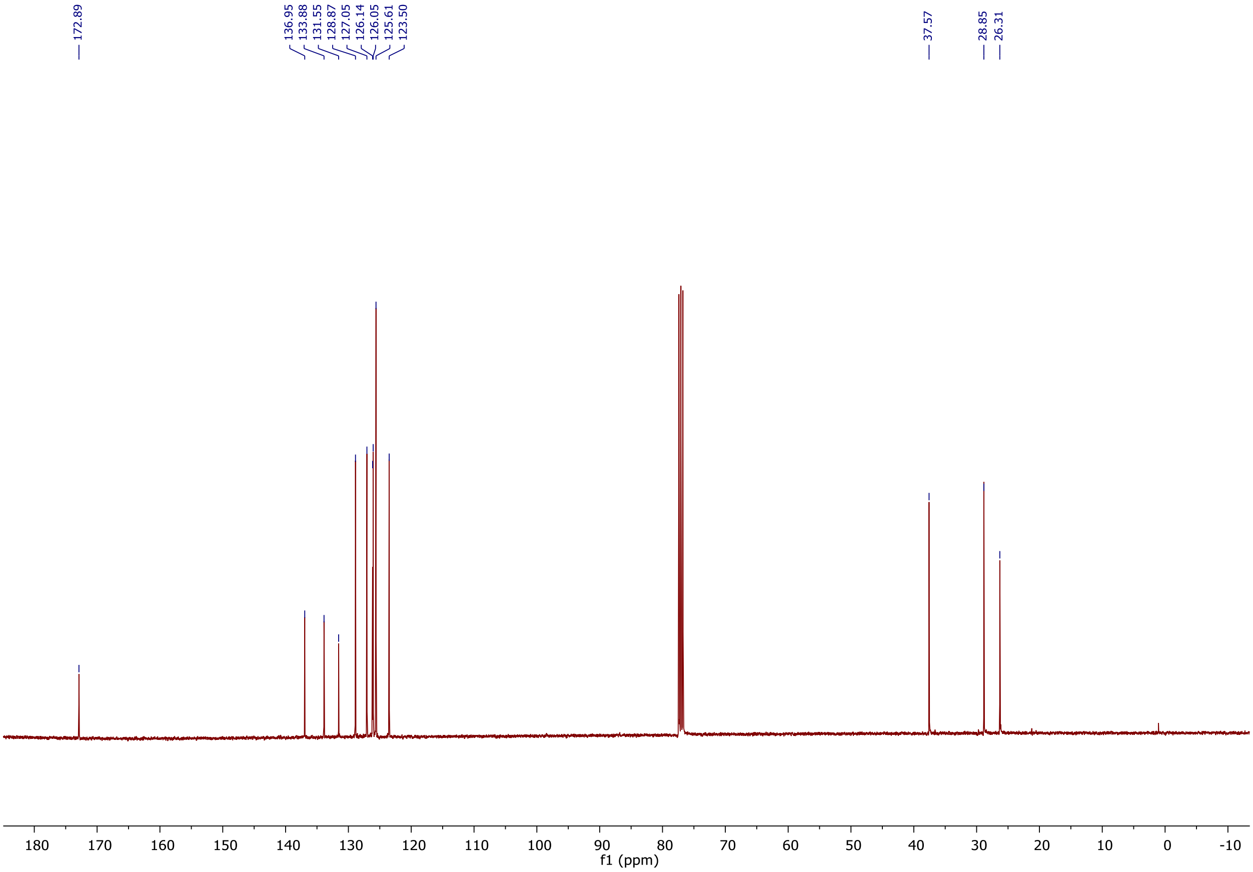

3-Benzyl-1-methylpiperidin-2-one (30)

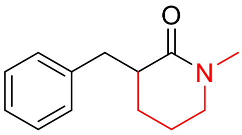

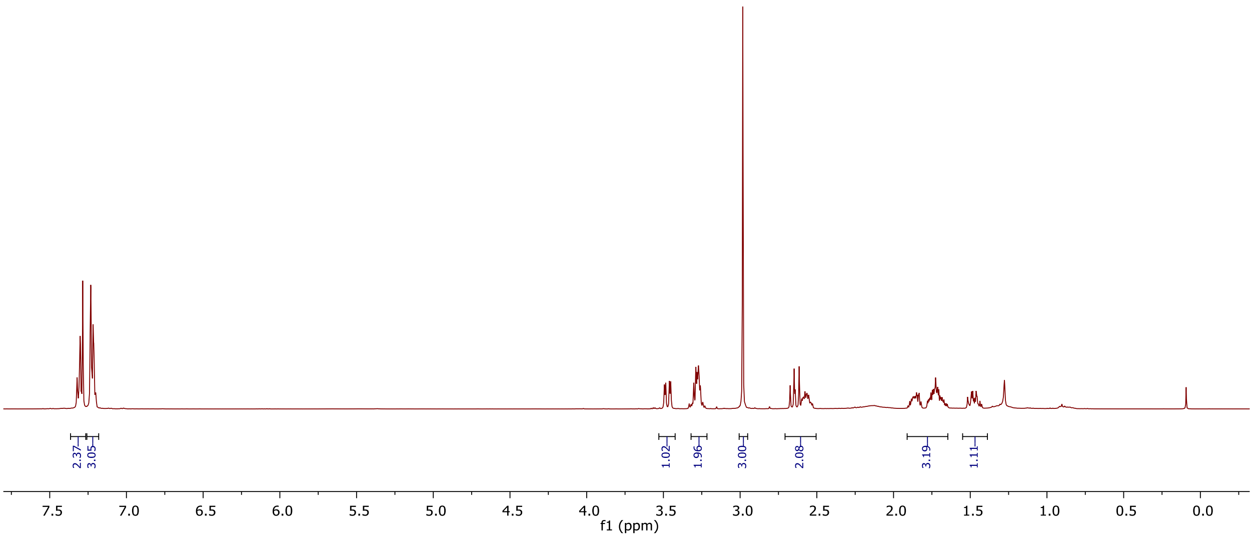

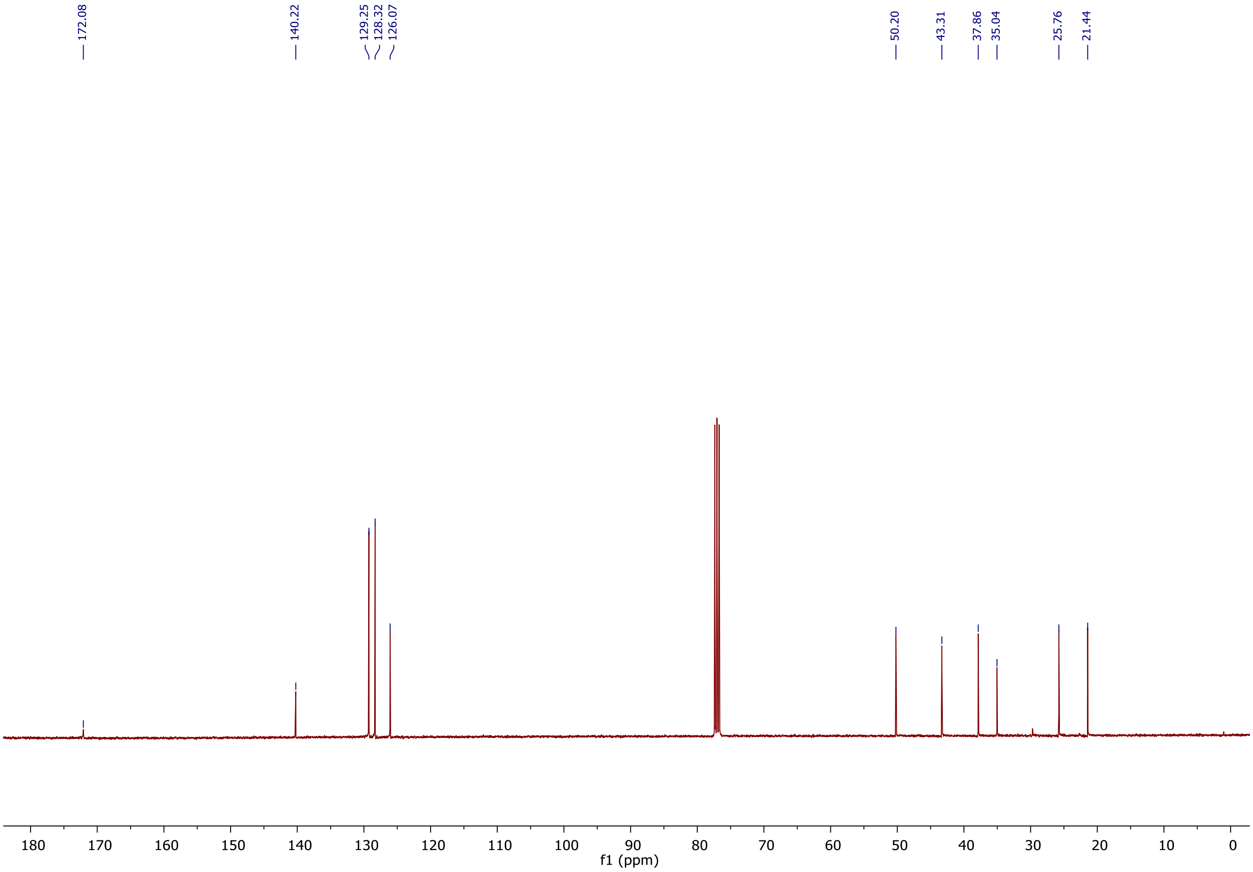

Supplement: SC-011-D0SC02948C-s001 [file SC-011-D0SC02948C-s001.pdf]
